# Estimating prevalence and modelling correlates of HIV test positivity among female sex workers, men who have sex with men, people who inject drugs, transgender people and prison inmates in Sierra Leone, 2021

**DOI:** 10.1186/s12981-023-00566-4

**Published:** 2023-09-27

**Authors:** Duah Dwomoh, Issata Wurie, Yvonne Harding, Kojo Mensah Sedzro, Joseph Kandeh, Henry Tagoe, Christabel Addo, Daniel Kojo Arhinful, Abdul Rahman Cherinoh Sessay, James Lahai Kamara, Kemoh Mansaray, William Kwabena Ampofo

**Affiliations:** 1 Department of Biostatistics, School of Public Health, College of Health Sciences, University of Ghana, Accra, Ghana; 2 Health and Education Quality Systems Strengthening, Freetown, Sierra Leone; 3 Department of Epidemiology, College of Health Sciences, Noguchi Memorial Institute for Medical Research, University of Ghana, Accra, Ghana; 4 John Snow Resarch & Training Institute Inc, Accra, Ghana; 5 National HIV/AIDS Secretariat, Freetown, Sierra Leone; 6 Department of Virology, College of Health Sciences, Noguchi Memorial Institute for Medical Research, University of Ghana, Accra, Ghana

**Keywords:** HIV/AIDS, Transgender people, Men who have sex with men, People who inject drugs, Female sex workers, Prison inmates

## Abstract

Key populations (KPs) are particularly vulnerable to HIV infection and efforts to prevent HIV infections among KPs have been less successful, largely due to existing laws and legislation that classify the groups as illegal. Understanding the HIV infection pathway and the burden of HIV infection among Female Sex Workers (FSWs), Transgender people (TG), Men who have sex with Men (MSM), People who Inject Drugs (PWID), and Prison Inmates (PIs) is critical to combatting the HIV epidemic globally. This study aims to estimate HIV prevalence and model the risk factors of HIV positivity rate among the aforementioned KPs in Sierra Leone. This study used Time Location Sampling, Respondent Driven Sampling (RDS), and Conventional cluster Sampling designs to generate a representative sample of FSWs, MSM, TG, PI, and PWID. HIV prevalence and the corresponding 95% confidence intervals among each KP were estimated by adjusting for sampling weight using the logit-transformed confidence intervals. To determine correlates of HIV test positivity among KPs, a multivariable modified Poisson regression model that adjusts for RDS survey weights was used and sensitivity analysis was conducted using a multivariable logistic regression model with cluster robust standard errors. The prevalence of HIV among FSWs in the six regional headquarter towns was estimated to be 11.8% (95% CI: 7.9–17.1); MSM was 3.4% [95% CI: 1.9–5.8]; TGs was 4.2% (95% CI: 2.9–6.1); PWIDs was 4.2% (95% CI: 2.7–6.4) and PI was 3.7% (95% CI: 1.4–9.6). The correlates of HIV test positivity among KPs and PIs include HIV-related knowledge, marital status, district, income, age and sex of KP, level of education, alcohol intake, injecting drugs, and use of lubricants. HIV prevalence is relatively high among FSWs, MSMs, PWID, and TGs as compared to the previous estimate of the general population. There is a need to scale up and strengthen evidence-based HIV prevention interventions such Pre-Exposure Prophylaxis and needle and syringe exchange programmes targeting KPs, including prison inmates. Government must scale up both non-clinical and clinical routine HIV and STI testing and counseling services at the correctional center and drop-in centers for KPs screening/testing, and ensure that services are responsive to the needs of KP.

**Supplementary Information:**

The online version contains supplementary material available at 10.1186/s12981-023-00566-4.

## Introduction

The UNAIDS formulated and implemented the UNAIDS 2016–2021 Strategy and the Global AIDS Strategy 2021–2026 to mitigate the impact of the HIV pandemic, reduce the spread of HIV and improve treatment outcomes and quality of life among persons living with HIV [1]. The strategy emphasized the need to enhance access to HIV services among key populations (KPs) (Female sex workers (FSW), Men who have Sex with Men (MSM), Transgender people (TG), and People Who Inject Drugs (PWID)) as a more viable channel to achieving this goal. The UNAIDS also set targets to reduce the number of people newly acquiring HIV and of AIDS-induced deaths to fewer than 500 000 per year by 2020 and fewer than 200 000 by 2030 respectively [1]. KPs and Prison Inmates (PI)) in sub-Saharan Africa (SSA) are at greater risk of HIV infection [2–5]. Trends analysis of the HIV epidemic shows that SSA possesses the highest burden of HIV and approximately 8% of new HIV infections globally and with about 20% infections outside SSA are among PWID [6, 7]. The HIV epidemic in Sierra Leone is considered mixed, generalized, and heterogeneous but concentrated in KPs with HIV prevalence in the general population estimated as 1.6% [8]. The epidemic affects different population sub-groups and all sectors through multiple and diverse transmission dynamics. The prevalence among the general population appears low, it has however shown an almost 100% increase, from 0.9% to 2002 to 1.7% in 2020 and the total number of people who have died from AIDS-related causes per 100 000 population was approximately 33 [9]. Several factors may have contributed to the observed trend including the increasing activities of key populations (MSM, FSWs, Transgender, PWID) who may be heterosexual and are at a higher risk of spreading infection.

From the national spectrum data, an estimated 78, 667 people are living with HIV (PLHIV) in Sierra Leone. Although the prevalence of HIV among the general population appears low in Sierra Leone, some populations are at higher risk of contracting and transmitting HIV. These include FSWs, MSM, TG, PWID, and PIs. Although these KPs are at high risk for HIV infection globally, they are socially marginalized and experience higher levels of stigma. In addition, epidemiologic and HIV prevention service data for FSW, MSM, PWID, and TG persons remain sparse [10]. This makes it difficult to track them in HIV program registers and impedes efforts to assess the effectiveness of HIV and other STI-related services.

Data on HIV infection among these populations who are at a higher risk for HIV infection are limited, although the existence of vulnerable groups and high-risk behaviors in Sierra Leone has been documented [8]. This study aims to estimate the prevalence of HIV and identify HIV-related risk factors among KPs to serve as a benchmark for assessing the progress of reaching 95-95-95 UNAIDS targets in Sierra Leone.

## Methods

### Study population and sampling

The study was conducted in the six regional headquarter towns of Sierra Leone: Bombali, Port Loko, Kenema, Kono, Bo, and the Western region (Western Area Urban, Western Area Rural) between 15th April 2021 to 31st December 2021.

The study involved FSWs, MSM, TG, PWID, and PIs in Sierra Leone. In this study, FSW were defined as females aged 18 years or older, who exchanged sex for money or favor as a source of income in the last 6 months. MSM were defined as any male 18 years and above who had engaged in insertive or receptive sexual intercourse (oral, anal) with other men at least once in the 6 months preceding the survey. PWID: any person, male or female, aged 18 years and above who had injected narcotic/illicit drugs recreationally at least once in the past 6 months preceding the survey period. TG: a person who had a gender identity that is different from his or her sex at birth. A TG may be male to female (female appearance) or female to male (male appearance) and described as ‘he’ or ‘she’ according to their gender identity, i.e. the gender that they were presenting, not their sex at birth. PI: Male and females in detention at correctional service centers in Sierra Leone during the time of the survey. All respondents must be residents in Sierra Leone for at least 12 months and provided either written or verbal informed consent before they were recruited into the study.

### Sampling design

**Fig. 1:**
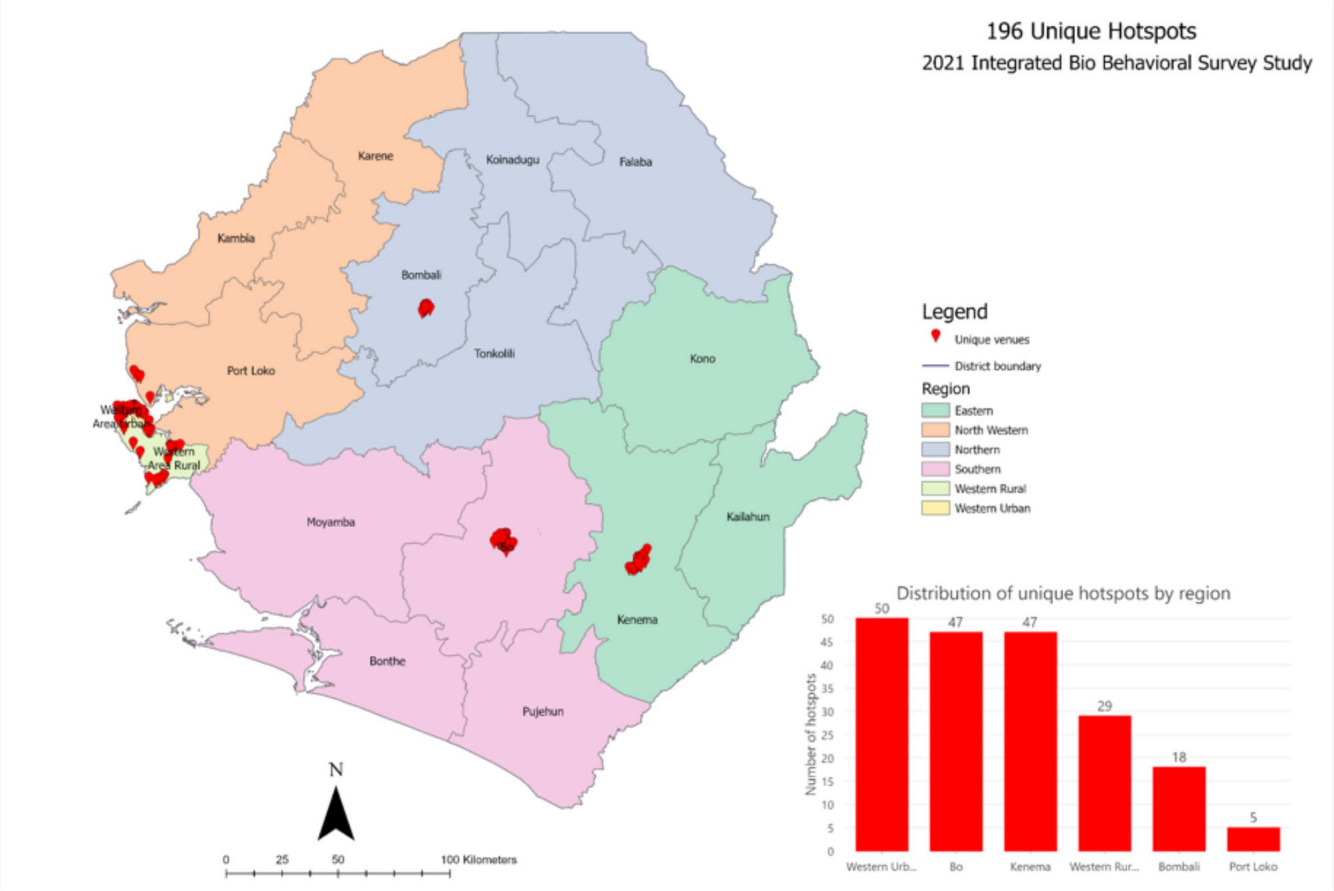
Distribution of Hotspots verified across 6 Regions in Sierra Leone

Time Location Sampling (TLS) method was used to generate a representative sample of FSW in all regions. TLS involves documenting the specific place of availability of FSWs waiting for their client, the days, and the time to reach them. It has previously been described as optimal for hard-to-reach populations [11]. A total of 196 unique FSW hotspots were identified based on the extensive literature review of previous key population studies, the FSW mapping exercise conducted in Sierra Leone for the current study, information from NGOs, CBOs that provide services to these KPs, hotspots observation, and key informant interviews on-site before the Integrated Bio-behavioral Surveillance Survey (IBSS) (Fig. 1).

**Fig. 2:**
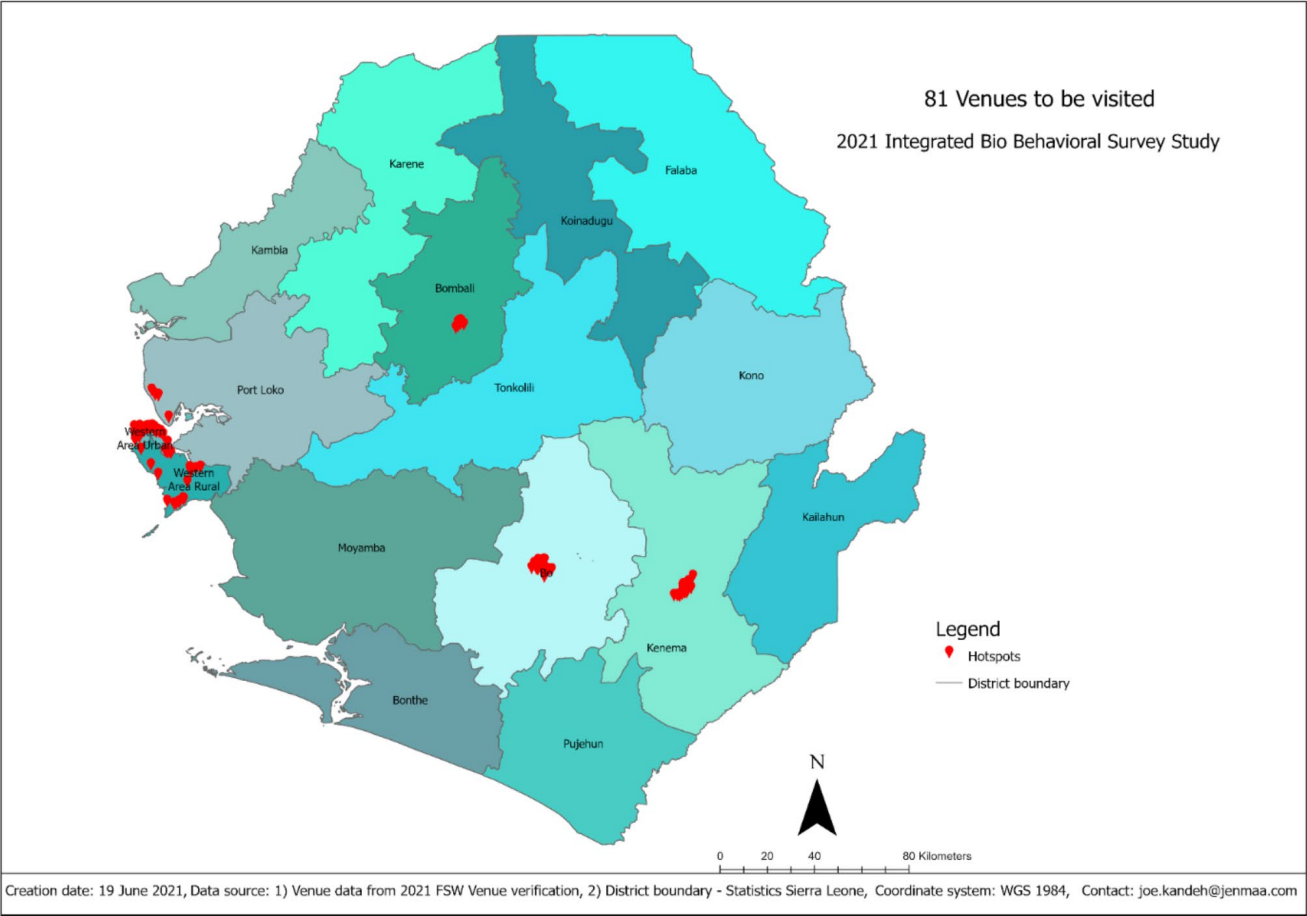
Distribution of 81 Hotspot venues

We constructed a sampling frame for each time-location of venues for FSWs across the six regions. The variables included in the sampling frame are as follows: location or venue ID, location/venue name, time, and the estimated measure of size (EMoS), that is the number of FSWs who meet the approach criteria at a specific venue at a specific time, minimum and maximum EMoS. The average number of FSWs per time-location venue was 20 (minimum = 3, maximum = 400) from 692 time-specific-location venues (hotspots) for the FSWs across the six regions. The estimated number of clusters (hotspots) visited during the study was 81 [the sample size for FSW (1627) / Average EMoS (20)] (Fig. 2).

The interviews at the hot spots were typically conducted between 4 p.m. and 11 pm on the selected day of the week. All FSWs that met the inclusion criteria and consented to the study were interviewed at specified locations around the hotspots.

The Respondent Driven Sampling (RDS) was used to sample PWID, MSM, and TG populations, whose locations could not be easily mapped, and no complete list of potential KPs exist, but members of KPs are socially networked with one another, and individuals can identify each other as part of the KP group and will be willing to recruit others from the network. RDS is based on the principles of chain-referral sampling and combines snowball sampling (in which participants recruit other participants) with a mathematical model to compensate for the fact that participation is not random. RDS relies on respondents at each wave to select the next wave of participants. RDS begins with the selection of seeds (specific survey participants recruited to enable access to the target group in RDS) and these seeds were identified during the formative evaluation phase of the study with the help of stakeholders (CBOs, and CSOs that work with KPs). The seed is one of the KPs who is well-connected within their social networks (among their peers), well-regarded by their peers, supportive of the survey’s goals, in separate social networks, and not a member of CBO or NGO (to prevent oversampling of CBO / NGO member networks). The seed was given 3 recruitment coupons and instructed to give these coupons to persons in their social circle who are also KPs to facilitate long chains which may help the recruitment of diverse social networks. This process continued until the target sample sizes for each KP were achieved. RDS is faster and less expensive than other sampling methods such as time location sampling and the conventional cluster sampling approach [12].

For prison inmates, a conventional cluster sampling approach was used. A sampling frame was developed based on the list of prisoners and their respective cell numbers provided by the prison officers and a multi-stage stratified cluster simple random sampling technique was to generate representative samples for the prisoners across the six-regional quarter towns in Sierra Leone. For each prison, a random sample of cells were selected and all inmates in that cell who consented to be part of the study were recruited.

### Sample size calculation

To determine the minimum sample size needed for all respondents regardless of HIV status, adjusted for design effect and non-response rate, we used the method of sample size estimation proposed by Fleiss and Newcombe et al., [13, 14] that account for expected proportion of HIV-positives with viral load suppression. The sample sizes required for the RDS in estimating the prevalence of FSW, MSM, TG, and PWIDs were obtained as follows:

$$n=DEFF\times \frac{{Z}_{1-\frac{\alpha }{2}}^{2}{\mu }_{P}(1-{\mu }_{P})}{{e}^{2}P(1-NR)}\dots \left(1\right)$$

where $n$ is the required sample size regardless of the HIV status of the key population, is ${Z}_{1-\frac{\alpha }{2}}=1.96$ is the standard normal variate at $\alpha =5\%$ and ${\mu }_{P}=26\%$is the proportion of viral load suppression (VLS) in the HIV-positive population [9], $P$ is the assumed HIV prevalence in the survey population and $e=12\%$ is the margin error associated with the point estimates. $NR=5\%$ is the assumed non-response rate. This study used a design effect (DEFF) of 2. The UNAIDS 2019 Factsheet for Sierra Leone showed that HIV prevalence among FSW was 6.7%, MSM was 14%, PWID was 8.5%, Transgender people was 15.3%, and \ Inmates (8.7%) [9]. The total sample size needed based on the VLS prevalence and the prevalence of HIV among key populations adjusted for the design effect and non-response rate was 5150, distributed as follows: FSW=1627, MSM=779, TG=712, PWID=1282, Prison Inmates=750.

### Measures

The primary outcome measure of interest was the HIV status of each KP. This is a binary outcome measure coded 1 if KP tested positive and 0 otherwise. The exposures of interest included sociodemographic factors (age, education, marital status, religion, etc.), sexual and behavioral risk factors (condom and lubricant use, injecting drugs, hiv testing history, sexual history (number of sexual partners), intervention coverage (benefitted from HIV prevention interventions including condom distribution, pre- and post-exposure prophylaxis etc.), and HIV-related knowledge (can people reduce their chance of getting HIV by having just one uninfected sex partner who has no other sex partners?, can people reduce their chance of getting HIV by using a condom every time they have sex? can a person get HIV by sharing food with someone who is infected? etc.). Details of all the variables studied can be found in supplementary Tables 1–55.

### HIV testing

Counsellor and Laboratory team perform daily test kit validation using known positive and negative controls to check the systems including the tester performance and reagent integrity.

HIV testing was done on site using the RDT serial algorithm by the counsellors who are trained in phlebotomy and field testing given their experience in national testing, treatment and care program. Finger-prick were used to collect the blood droplet and test carried out as per protocol.

The testing of all the plasma and DBS samples were undertaken in a 4-star SLIPTA Certified Laboratory with 24 hour energy capacity. All assay were carried out by selected members of the national quality assurance (QA) team lead by the national Public Health Manager. Test results were recorded and transferred into the log book and later to the survey electronic tools to confirm reactive and QA negative samples results obtained in the field. Laboratory testing for confirmatory and quality assurance was performed on all reactive samples and 10% negative samples to capture true negative. The assays was as per Confirmatory Testing Algorithm of 4th Generation Enzyme Linked Immunosorbent Assay (ELISA) and using Nucleic Acid Test (NAT)– on Gene Xpert and where samples have been compromised they were subjected to Reverse Trancriptase Polymerase Chain Reaction on ROCHE platform. As part of the confirmatory algorithm, serological testing for HIV antibodies using validated ELISA kits were perform on all viable samples. Criteria set by the manufacturers for valid results were adapted to formulate approved Standard Operating procedures (SOP). High level skilled laboratory team trained in both serological and molecular assay including conventional and automated Reverse Transcriptase - Polymerase Chain Reaction (RT-PCR) were used.

The selected HIV test kits were as follows:

**Test 1**: Screening: Biorex Anti HIV 1 and 2( UK); is a rapid, qualitative screening, in vitro diagnostic test, which detects all known subtypes of HIV with a simple one-step procedure for serum/plasma or a two-step procedure for whole blood or plasma.

**Test 2**: RDT retesting : Alere (Abbott) Determine™ HIV-1/2 test kit is a rapid, qualitative screening, in vitro diagnostic test, which detects all known subtypes of HIV with a simple one-step procedure for serum/plasma or a two-step procedure for whole blood.

**Test 3**: Tie breaker: Trinity Biotech Uni-Gold™ HIV test is a single reagent assay for the detection of antibodies to human immunodeficiency virus types 1 and 2 in serum, plasma or whole blood. All the HIV results were entered into the study excel data base. All samples reactive for Test-1 were retested and any inconclusive results were resolved with the third test kit Uni-gold.

### Data management

Data for TG, MSM, and PWID were captured using an excel coupon manager that tracks coupons distributed over the study period and the Census and Survey Processing System (CSPro) electronic data capture which captures responses from the actual survey. We synchronized the laboratory databases that had records on the HIV status of the study participants with the data from the coupon manager.

### Analysis

The prevalence of HIV and the corresponding 95% confidence interval for each KP was determined using the logit-transformed confidence intervals. Variable selection into the multivariable models were based on statistical significance (p < 0.05) during the bivariate analysis using univariate regression model. We further quantify the effect of the aforementioned exposures on HIV prevalence using a modified Poisson regression model that reports adjusted prevalence ratio and a sensitivity analysis was conducted with the multivariable binary logistic regression model with a robust standard error that reports adjusted and odds ratio and their corresponding 95% confidence intervals. Poisson regression model with least absolute shrinkage and selection operator and Firth Logit model were used to address the problem from small sample bias of the maximum likelihood estimation assoaciated with conventional logistic regression model. All statistical analyses adjusted for RDS design weights and a p-value of less than 0.05 was considered statistically significant. The specialized analyses within RDS-A were used to adjust for social network size and homophily within networks. Network size for KPs was determined by the following set of questions: “How many PWID/Transgender/MSMs in the study area: of these, how many do you know by name and they know yours?”; “Of those PWID/Transgender/MSMs, about how many of them would you say is 18 years of age and older?”; “Of those PWID/Transgender/MSMs how many would be willing to participate in the study?” The answer to the last question was used as the network size question. The RDSAT produced survey individualized weights using Heckathorn’s original estimator. The data along with the individual RDS-A generated weights were exported into Stata version 17.0 (StataCorp, College Station, Texas, US) for bivariate and multivariable regression analyses. Geographic information system data and mapping of hotspots were analyzed using Arc GIS software.

### Ethical approval

Ethical approval was obtained from the Sierra Leone Ethical Review Committee. All KPs provided informed consent. The unique study identifier for each KP obtained from the biometric device de-identified the study data that were collected and analyzed.

## Results

### Characteristics of the key populations

The average response rate among the FSWs was 88.0%, TGs = 72.0%, MSM = 72%, PWID = 90.1% and Prison Inmates = 60.8%. A total of 1430 FSWs were enrolled from 196 hotspots for the IBSS. Of the 1,430 FSWs, the average age was 24.5 years (SD = 5.5, youngest = 18 years, oldest = 53 years). A total of 562 MSMs were studied. The average age of the MSMs was 24.3 years with the youngest and oldest being 18 years and 49 years respectively. We studied 561 TGs with an average age of 22.5 years (youngest = 18 years; oldest = 52 years). A total of 1,155 PWID were interviewed with an average of 26 years (youngest = 18 years, oldest = 71 years). In all, 456 prisoners were enrolled in the study from eight correction centers in Sierra Leone. The average age of the prisoners was 32.4 years with the youngest and oldest prisoners being 18 and 74 years old respectively. About nine in every ten (n = 390, 85.5%) prison inmates interviewed were males. The detailed distribution of the sociodemographic characteristics, behavioural and sexual risk behaviour, intervention coverage, etc., of the study participants can be found in Supplementary Tables 1–10. In addition, all the bivariate analysis of factors associated with HIV can be found in Supplementary Tables 11–55. The subsequent analyses present the results from the multivariable regression models.

### Prevalence of HIV among FSW, MSM, TG, PWID, and prison inmates

HIV prevalence among FSWs in the six regional headquarter towns was estimated to be 11.8% (95% CI: 7.9–17.1); MSM was 3.4% [95% CI: 1.9–5.8]; TGs was 4.2% (95% CI: 2.9–6.1); PWIDs was 4.2% (95% CI: 2.7–6.4) and PI was 3.7% (95% CI: 1.4–9.6). The prevalence of HIV stratified by the sociodemographic characteristics of the study participants and factors that independently influence the prevalence of HIV can be found in Supplementary Tables 11–55.

### Factors associated with HIV among FSW in Sierra Leone

The results in Table 1 showed that marital status, the district where the FSW live and operate, monthly income, HIV and AIDS related knowledge, and injecting drugs are associated with higher HIV prevalence among FSW in Sierra Leone. The prevalence of HIV among FSWs that live/work in Western urban areas was approximately 4 times (adjusted prevalence ratio; aPR = 3.6, 95% CI: 1.7–7.5, p < 0.05) the prevalence of HIV among those that live/work in Bombali. The prevalence of HIV among FSWs who have ever or are currently being in a relationship was approximately 2 times the prevalence of HIV among FSWs who have never been married or cohabiting ( aPR = 1.8, 95% CI:1.4–2.3, p < 0.05). The prevalence among FSWs who earn on average 50 USD or higher a month was twice the prevalence of HIV among FSWs who earn less than 50 USD (aPR = 1.8; 95% CI: 1.2–2.7; p < 0.05). HIV prevalence was lower among FSWs that provided correct responses to the following questions: Can people reduce their chance of getting HIV by having just one uninfected sex partner who has no other sex partners and whether people can get HIV from mosquito bites.

**Table 1:** Factors associated with HIV among FSW in Sierra Leone

Factors	Adjusted prevalence ratio from Modified Poisson Regression Model (N = 1430)	Adjusted Odds ratio from the binary logistic regression model (N = 1430)
	aPR [95% CI]	aOR [95% CI]
**Educational level**		
None	1	
Primary	1.51[0.95–2.39]	1.70 [0.95–3.05]*
JSS	0.85 [0.51–1.43]	0.84[0.44–1.59]
SSS/ Technical Vocational	0.72 [0.39–1.35]	0.68[0.32–1.45]
Higher	1.01 [0.12–8.37]	0.99[0.08–12.60]
**Marital status**		
Never married/cohabited	1	
Others (Married, divorced, widowed)	1.76 [1.36–2.28]***	2.05[1.50–2.78]***
**District**		
Bombali	1	1
Port Loko	2.36 [0.55–10.12]	2.54 [0.49–13.25]
Kenema	1.93 [0.84–4.42]	1.98 [0.80–4.88]
Bo	2.45 [0.81–7.48]	2.63 [0.73–9.46]
Western Urban	3.57 [1.70–7.53]***	4.27 [1.90–9.58]***
Western Rural	0.10 [0.38–2.62]	0.96 [0.34–2.72]
**Household income categorized**		
< 50 USD	1	1
50 USD or more	1.83 [1.24–2.70]***	2.12 [1.28–3.51]***
**Age first receive money for sex**		
Below 18	1	1
18 + years	1.23 [0.79–1.92]	1.30 [0.77–2.18]
**Can people reduce their chance of getting HIV by having just one uninfected sex partner who has no other sex partners?**		
Wrong response	1	1
Correct response	0.64 [0.50–0.82]***	0.57 [0.41–0.79]***
**Can people get HIV from mosquito bites?**		
Wrong response	1	1
Correct response	0.70 [0.51–0.96]**	0.63 [0.43–0.93]**
**Number of clients paying partners in the last 3 months**		
1–5	1	1
6–10	0.88 [0.47–1.62]	0.84 [0.39–1.83]
11–19	0.62 [0.35–1.10]	0.54 [0.29–1.03]
20 or more	0.85 [0.56–1.30]	0.80 [0.46–1.38]
**Inject drugs in the past 3 months**		
No	1	1
Yes	1.66 [0.98–2.83]	2.12 [1.01–4.46]**

Abbrevaiation: FSW: Female Sex Workers, aPR: adjusted Prevalence Ratio, aOR: adjusted odds ratio; CI: Confidence interval. P-value notation: ***p < 0.001, **p < 0.01, *p < 0.05.

### Multivariable analysis of factors associated with HIV among MSM

Table 2 shows the multivariable analysis of factors associated with higher HIV prevalence among MSM in the six regional head-quarter towns. The age of the MSM was the only variable that was found to be statistically significant after fitting the multivariable model. The prevalence of HIV among the MSM aged 30 years was approximately 5 times as high as the prevalence of HIV among MSM aged 18–24 years (aPR = 4.6, 95% CI: 1.4–15.2; p < 0.05).

**Table 2:** Multivariable analysis of factors associated with HIV among MSM

Sociodemographic characteristics	Adjusted prevalence ratio from double selection Lasso Poisson Regression Model adjusting for sampling weight from RDS (N = 562)	Adjusted Odds ratio from Firth Penalized maximum likelihood logistic regression model adjusting for sampling weight from RDS (N = 562)
	aPR [95% CI]	aOR [95% CI]
**Age in years**		
18–24 years	1	1
25–29 years	3.61 [0.99–13.11]*	3.48 [0.90-13.52]
30+	4.57 [1.37–15.24]*	4.62 [1.04–20.38]*
**Income**		
Less than 500,000 Leone	1	1
500,000 Leone or higher	1.41 [0.53–3.76]	1.42 [0.48–4.22]
**Employment status**		
Employed	1	1
Unemployed	0.56 [0.22–1.45]	0.57 [0.18–1.76]
**Marital status**		
Married/divorced etc.	1	1
Single	0.57 [0.23–1.39]	0.53 [0.17–1.61]
**Religion**		
Christian	1	1
Moslem	0.46 [0.15–1.44]	0.46 [0.15–1.39]
**Province**		
Northern	1	1
Eastern	0.92 [0.06-15.00]	0.94 [0.09–9.48]
Southern	2.71 [0.16–45.85]	2.72 [0.25–29.45]
Western	6.91 [0.91–52.40]	5.44 [0.92–32.18]
**Ever had vaginal sex**		
Yes	1	1
No	0.41 [0.09–1.86]	0.46 [0.11–1.98]

Abbreviations: MSM: Men who have sex with men, aPR:Adjusted Prevalence Ratio, aOR:Adjusted odds ratio; CI: Confidence interval. P-value notation:***p < 0.001, **p < 0.01, *p < 0.05.

### Factors associated with HIV among TGs in Sierra Leone: a multivariable regression analysis

The results showed that parity, ethnicity, alcohol, lubricant use, and HIV and AIDs related knowledge are associated with higher HIV prevalence among TGs in Sierra Leone (Table 3). The prevalence of HIV among TG that had 3 or more children was approximately 8 times the prevalence of HIV among those that had no children (adjusted prevalence ratio; aPR = 7.5, 95% CI: 3.2–17.6, p < 0.05). The prevalence of HIV among TGs who were from the Temne tribe was approximately 4 times the prevalence of HIV among TGs who were from the Mende tribe ( aPR = 3.8, 95% CI:1.6–9.2, p < 0.05). The prevalence of HIV among TGs who drank alcohol increased by approximately 2.9 compared to TGs that never drank alcohol (aPR = 2.86; 95% CI: 0.41–5.56; p < 0.05). The HIV prevalence was lower among TGs that provided correct responses to the following question: can a healthy-looking person have HIV (aPR = 0.24; 95% CI: 0.11–0.56; p < 0.05).

**Table 3:** Factors associated with HIV among transgender people in Sierra Leone

	Adjusted prevalence ratio from Modified Poisson Regression Model (N = 561)	Adjusted Odds ratio from the binary logistic regression model (N = 561)
Characteristics	aPR [95% CI]	aOR [95% CI]
**Monthly income (Leone)**		
Less than 500,000	1	1
5,000,000 or higher	1.62 [0.76–3.44]	2.00 [0.75–5.33]
**Parity**		
No child	1	1
1–2 children	2.06 [0.93–4.56]	2.30 [0.88–6.03]
3 or more	7.54 [3.24–17.55]***	13.72 [4.02–46.89]***
**Ethnicity**		
Mende	1	1
Temne	3.80 [1.58–9.17]**	4.08 [1.28–13.05]*
others	4.92 [2.01–12.05]***	5.75 [1.64–20.11]**
**Alcohol use**		
Never	1	1
Ever taken	2.86 [1.41–5.56]**	3.57 [1.49–8.33]**
**Lubricant use**		
Yes	1	1
No	0.14 [0.03–0.61]*	0.10 [0.02–0.51]**
**Microbicide use**		
Yes	1	1
No	0.62 [0.31–1.26]	0.59 [0.23–1.50]
**Can a healthy-looking person have HIV?**		
Inadequate knowledge	1	1
Adequate knowledge	0.24 [0.11–0.56]**	0.17 [0.05–0.56]**

Abbrevaiation: TG: Transgender, aPR: adjusted Prevalence Ratio, aOR: adjusted odds ratio; CI: Confidence interval. P-value notation: ***p < 0.001, **p < 0.01, *p < 0.05.

### Multivariable analysis of factors associated with HIV among PWID

The results from the multivariable Poisson with double selection least absolute shrinkage and selection operator and the binary logistic firth penalized maximum likelihood regression showed that HIV prevalence was higher among females compared to male PWIDs. The prevalence of HIV among female PWIDs was approximately 5 times as high as the prevalence of HIV among males (aPR = 5.4; 95% CI:2.9–9.8). The detailed multivariable analysis of factors associated with HIV among PWIDs can be found in Table 4.

**Table 4:** Multivariable analysis of factors associated with HIV among PWID

Sociodemographic characteristics	Adjusted prevalence ratio from double selection Lasso Poisson Regression Model adjusting for sampling weight from RDS(N = 1155) aPR [95% CI]	Adjusted Odds ratio from Firth Penalized maximum likelihood logistic regression model adjusting for sampling weight from RDS (N = 1155) aOR [95% CI]
**Sex at birth**		
Male	1	1
Female	5.35 [2.92–9.79]***	6.30 [3.04–13.03]***
**Education level**		
None/primary	1	1
JHS	0.92 [0.45–1.88]	0.91 [0.40–2.12]
SHS/Tech	0.40 [0.18–0.89]*	0.38 [0.15–0.97]*
Higher	1.15 [0.38–3.53]	1.22 [0.36–4.20]
**Province**		
Northern	1	1
Eastern	0.57 [0.23–1.38]	0.55 [0.21–1.43]
Southern	0.16 [0.02–1.30]	0.22 [0.04–1.20]
Western	0.67 [0.34–1.32]	0.65 [0.30–1.44]
**Can people get HIV from mosquito bites?**		
Inadequate knowledge	1	1
Adequate knowledge	0.59 [0.33–1.05]	0.56 [0.28–1.13]

Abbreviation: PWID: People who inject drugs, aPR: Adjusted Prevalence Ratio, aOR: Adjusted odds ratio; CI: Confidence interval. P-value notation: ***p < 0.001, **p < 0.01, *p < 0.05.

### Factors associated with HIV among Prisoners in Sierra Leone

The results showed that the sex of prisoners, educational level, marital status, age, and HIV and AIDs related knowledge, were associated with higher HIV prevalence among prisoners in Sierra Leone (Table 5). The prevalence of HIV among female prisoners was approximately 6 times as high as the prevalence of HIV among male prisoners (adjusted prevalence ratio; aPR = 5.51, 95% CI: 4.2–7.22, p < 0.05). The prevalence of HIV among prisoners who have ever been married or currently been in a relationship was approximately 3 times the prevalence of HIV among prisoners who have never been married or cohabiting (aPR = 2.9, 95% CI:2.1–4.0, p < 0.05). HIV prevalence among prisoners with high HIV-related knowledge was 10 times as high as the prevalence of HIV among prisoners with low HIV-related knowledge (aPR = 9.8, 95% CI:7.2–13.4). Prisoners aged 40 years and above had about 3 times higher prevalence of HIV compared to those aged 18-24years old (aPR = 2.8, 95%CI:1.0- 7.3). Prisoners with secondary level education or higher had about 2 times higher prevalence of HIV compared to those without any formal education (aPR = 2.1, 95% CI: 1.4–2.9; Table 5).

**Table 5:** Factors associated with HIV among Prisoners in Sierra Leone

Factors	Adjusted prevalence ratio from Modified Poisson Regression Model (N = 456)	Adjusted Odds ratio from the binary logistic regression model (N = 456)
	aPR [95% CI]	aOR [95% CI]
**Sex at birth**		
Male	1	1
Female	5.51[4.2–7.22]*	7.85[5.62–10.98]*
**Educational level**		
None	1	1
Primary	2.15[0.65–7.1]	2.31[0.57–9.26]
JSS	2.07[0.75–5.67]	2.24[0.76–6.62]
SSS/ Technical Vocational/ higher	2.05[1.43–2.95]*	2.54[1.64–3.94]*
**Ever married/Cohabited**		
Yes	1	1
No	2.88[2.08–4.00]*	3.52[2.26–5.49]*
**Age in years**		
17-24years	1	1
25-29years	0.18[0.05–0.63]	0.15[0.05–0.49]
30-39years	1[0.23–4.31]	0.98[0.17–5.7]
40 + years	2.75[1.04–7.27]*	3.48[1.07–11.32]*
**HIV Knowledge Level**		
Low (0–6/13)	1	1
Moderate (7–10/13)	2.73[1.13–6.62]*	3.08[1.23–7.72]*
High(11–13/13)	9.79[7.17–13.37]*	16.77[8.48–33.16]*

## Discussions

This study determines the prevalence of HIV and associated risk factors among four key populations and prison inmates in Sierra Leone. The HIV prevalence was high among KPs compared to the general population of Sierra Leone which is similar to findings from Djomand et al., 2013 [15] which showed that the HIV epidemic is highly prevalent among KPs including FSW and men who sell or trade sex, MSM, PWID, TG women who have sex with men, prisoners and detainees in Africa. It also affirms the findings of the UNAIDS report in 2021 that showed that gay men and other MSM have about 25 times greater risk than heterosexual men, FSWs have 26 times greater risk than women in the general population, TG women had 34 times greater risk than other adults and PWIDs have 35 times greater risk than people who do not inject drugs [16]. In West Africa, KPs are usually involved in high-risk practices that lead to higher HIV infection and other sexually transmitted diseases [17]. Available evidence shows that HIV prevalence fluctuates across and within countries for both MSM and FSWs and may be five to ten times as high as that of the general population. For instance, across the West African Sub-region, HIV prevalence varied from 15.9% in The Gambia to 68% in Benin among FSWs, whereas it ranged from 9.8% in The Gambia to 34.9% in Nigeria for MSM [8]. The prevalence was found to be disproportionately high among FSWs compared to the other KPs (MSM, TG, PWID) and PI. The HIV prevalence among FSW documented in the 2021 National AIDS Secretariat report is approximately 7 times higher than the general population prevalence rate (1.6%) [8]. In addition, an increase in prevalence was observed among FSWs from 8.5% to 2010 to 11.8% in 2021 highlighting the urgent need to increase HIV prevention education and access to HIV testing and counseling as part of the mitigation strategies. Among all the KPs that were studied, it was observed that women carry the highest burden of HIV similar to what has been reported elsewhere which shows that women have a higher HIV prevalence and incidence than men [18]. However, the estimated prevalence of HIV among FSWs in Sierra Leone was found to be lower compared to some countries in East Africa [19, 20] but higher compared to some West African countries [21]. Compared to other West-African countries (Ghana, Nigeria), the prevalence of HIV among MSM was lower [22, 23]. The HIV prevalence among Prisoners is higher compared to Ghana [24]. Several factors may contribute to the higher prevalence of HIV among KPs and PIs. Evidence from the medical literature shows that KPs continue to be marginalized and criminalized for their gender identities and expression, sexual orientation, and livelihoods and these have a negative effect on HIV outcomes as they are unable to seek care and proper guidelines geared toward reducing HIV infection and improving treatment outcomes [25]. This differential access to care for KPs has been advocated by the urgent need for research to understand factors impacting adherence to and retention in care among HIV-positive youth and adolescents from KPs [26].

On the contrary, the adoption of laws that prohibit nondiscrimination promotes the existence of human rights institutions and responds to gender-based violence (GBV) correlates with better HIV outcomes [25]. GBV has been shown to increase the probability of acquiring HIV infection for women and girls, and among women living with HIV, it can lead to reduced access and adherence to treatment [27, 28]. In addition, women sometimes have a harder time navigating safe sex practices, and women are more susceptible to acquisition of HIV through sexual behaviors. For instance, among PWID who are women, many of the infections may be a result of having sex with their injecting partners or paying for drugs through sex acts or other risky practices.

KPs are usually denied important HIV prevention services and for PWIDs proper injection practices are largely ignored. The coverage of prevention programs for gay men and other MSM is still low, even in many high-income countries. Coverage of prevention programs for TG people is woefully inadequate in all but a handful of countries. Coverage of prevention programs among FSWs in Western, Eastern, and Southern Africa is still low [25]. People in prisons and other closed settings are often not provided HIV services, despite the relative ease of reaching them [25].

The correlates of HIV test positivity among KPs and PIs found in this study include HIV-related knowledge, marital status, district, income, age and sex of KP, level of education, alcohol intake, injecting drugs, and use of lubricants. This finding is similar to what has been previously reported in the medical literature [29–33]. Misusing alcohol or abusing drugs can impair judgment of an individual, leading a person to engage in unsafe sexual behaviors which is associated with higher risk of HIV infection.

Although standard statistical methods and more rigorous sampling designs were used to generate a representative sample for the KPs, our study has limitations. It is important to note that certain groups of KPs may be omitted from the study. For instance, FSWs that do not visit specific venues but operate through social media may not be included in the study. Similarly, age limit of 18 years as inclusion criteria, excluded large cohort of less than 16 years who are engaged in sex work.

The RDS design used sampling probability based on individual network size and peer-referral chain system, it is possible that certain groups of the target population may be under or over-represented. Although the seeds were carefully selected so that samples will represent a diverse network of the population, the RDS design may fail to capture these diverse networks of MSM, PWID, and TG population and the sample may not be entirely representative. Some inmates that were randomly selected to participate in the study were released before the survey. Given that females who inject drugs do not form strong network ties with their peers and have small, closed networks (heterophyllous) with males (boyfriends, husbands, or close male partners) who inject drugs it was difficult to conduct a comprehensive gender-based analysis. This is a crossectional study and causality cannot be inferred. Caution must be applied in interpreting the results especially when self-reported data were collected for some of the questions in the analysis and social desirability around certain behaviors could influenced the results.

## Conclusion

HIV prevalence among KPs was found to be high and interventions geared towards increasing access and uptake of HIV and AIDS-related services among KPs, should be Government priority. Government and non-governmental agencies must scale up both non-clinical and clinical routine HIV and STI testing services at the correctional centre and drop-in centers for KPs screening/testing and ensure that services are responsive to the needs of KP. We must provide specific training to CSOs, community and faith-based organizations, health care providers and other service providers to ensure a more friendly and supportive environment to encourage KPs to seek services when necessary.

### Electronic supplementary material

Below is the link to the electronic supplementary material.

Supplementary Material 1

## Data Availability

The data that support the findings of this study are available on request from the corresponding author. The data are not publicly available due to privacy or ethical restrictions.
