# Supplementary material for: Estimating prevalence and modelling correlates of HIV test positivity among female sex workers, men who have sex with men, people who inject drugs, transgender people and prison inmates in Sierra Leone, 2021
Source: AIDS Res Ther. 2023 Sep 27;20:70. doi: 10.1186/s12981-023-00566-4 (PMC10537076; doi:10.1186/s12981-023-00566-4)
Supplement: Supplementary file 1 — Supplementary Material 1 [file 12981_2023_566_MOESM1_ESM.docx]

**Estimating prevalence and modeling correlates of HIV test positivity among Female Sex Workers, Men who have Sex with Men, People who Inject Drugs, Transgender and Prison Inmates in Sierra Leone: Evidence from Integrated Bio-behavioral Surveillance Survey**

Duah Dwomoh^1^, Issata Wurie^2^ , Yvonne Harding^2^, Kojo Mensah Sedzro^3^, Joseph Kandeh^2^, Henry Tagoe^4^, Christabel Addo^3^, Daniel Kojo Arhinful^3^, Abdul Rahman Cherinoh Sessay^5^, James Lahai Kamara^5^, Kemoh Mansaray^5^, William Kwabena Ampofo^6^

**Supplementary** Table 1: Characteristics of the Female Sex Workers in Sierra Leone

| **Characteristics of FSWs in Sierra Leone** |  |  |
| --- | --- | --- |
| **Nationality** | Freq | Percent |
| Sierra Leone | 1411 | 98.67 |
| Liberia | 9 | 0.63 |
| Guinea | 7 | 0.49 |
| Other countries | 3 | 0.21 |
| **Mean age (SD)** | 24.89 (5.53) |  |
| **Age in years** |  |  |
| 18-24 years | 774 | 54.13 |
| 25-29 years | 380 | 26.57 |
| 30-39 years | 253 | 17.69 |
| 40+ years | 23 | 1.61 |
| **Ever attended school** |  |  |
| Yes | 1039 | 72.66 |
| No | 391 | 27.34 |
| **Highest level of education** |  |  |
| Primary | 204 | 19.63 |
| JSS | 452 | 43.50 |
| SSS/ Technical Vocational | 369 | 35.51 |
| Higher | 14 | 1.35 |
| **Currently, a student or enrolled as a learner** |  |  |
| Yes | 218 | 20.98 |
| No | 821 | 79.02 |
| **Current employment status** |  |  |
| Employed Full-Time | 406 | 28.39 |
| Employed Part-Time | 231 | 16.15 |
| Full-Time Student | 63 | 4.41 |
| Retired | 5 | 0.35 |
| Unemployed | 657 | 45.94 |
| Other | 58 | 4.06 |
| Don’t Know | 6 | 0.42 |
| No Response | 4 | 0.28 |
| **Median household income (LQ, UP)** | 200000 [600, 500000] |  |
| **Household income categorized** |  |  |
| **Less tha 500000 Leone** | 720 | 50.35 |
| 50000 Leone or higher | 302 | 21.12 |
| Missing | 408 | 28.53 |
| **Ever been married or Cohabited with a man** |  |  |
| Yes | 369 | 25.80 |
| No | 1061 | 74.20 |
| **Marital status at the moment** |  |  |
| Currently Married | 16 | 4.34 |
| Separated | 130 | 35.23 |
| Divorced | 32 | 8.67 |
| Widowed | 19 | 5.15 |
| Cohabitating | 97 | 26.29 |
| Single | 75 | 20.33 |
| **Religion** |  |  |
| Christian | 566 | 39.58 |
| Moslem | 859 | 60.07 |
| Traditional | 2 | 0.14 |
| No Religion | 3 | 0.21 |
| **Regions** |  |  |
| Northern | 448 | 31.33 |
| Eastern | 228 | 15.94 |
| Southern | 204 | 14.27 |
| Western | 550 | 38.46 |
| **District** |  |  |
| Bombali | 223 | 15.59 |
| Port Loko | 225 | 15.73 |
| Kenema | 228 | 15.94 |
| Bo | 204 | 14.27 |
| Western Urban | 388 | 27.13 |
| Western Rural | 162 | 11.33 |
| **Town/City name** |  |  |
| Makeni | 223 | 15.59 |
| Lingui | 225 | 15.73 |
| Kenema | 228 | 15.94 |
| Bo | 204 | 14.27 |
| Freetown | 388 | 27.13 |
| Waterloo | 63 | 4.41 |
| Grafton | 99 | 6.92 |
| **Ethnic affiliation** |  |  |
| Mende | 495 | 34.62 |
| Temne | 453 | 31.68 |
| Fula | 46 | 3.22 |
| Limba | 137 | 9.58 |
| Mandingo | 85 | 5.94 |
| Kono | 30 | 2.10 |
| Krio | 45 | 3.15 |
| Kuranko | 13 | 0.91 |
| Other (Specify) | 126 | 8.81 |
| **Type of venue** |  |  |
| Bar | 483 | 33.78 |
| Brothels | 221 | 15.45 |
| Night Club | 34 | 2.38 |
| Drinking Joint | 111 | 7.76 |
| Hotel | 30 | 2.10 |
| Private Home Parties | 40 | 2.80 |
| Resthouse | 272 | 19.02 |
| Rest Stop | 2 | 0.14 |
| Street | 28 | 1.96 |
| Other | 209 | 14.62 |
| **Record type of respondent** |  |  |
| Roamer | 826 | 57.76 |
| Seater | 604 | 42.24 |

Note: Standard Deviation abbreviated as SD, LQ=Lower Quartile, UP: Upper Quartile, JHS: Junior High School, SHS: Senior High School

**Supplementary Table 2: Sociodemographic characteristics of MSM in the six regional headquarter towns**

| **Sociodemographic characteristics** | **Frequency** | **Percent** |
| --- | --- | --- |
| **Age in years** |  |  |
| 17-24 years | 348 | 62.03 |
| 25-29 years | 128 | 22.82 |
| 30+ | 85 | 15.15 |
| **Education level** |  |  |
| None/primary | 49 | 8.73 |
| JHS | 68 | 12.12 |
| SHS/Tech | 391 | 69.70 |
| Higher | 53 | 9.45 |
| **Income** |  |  |
| **Less than 500000 Leone** | 407 | 76.36 |
| 50000 Leone or higher | 126 | 23.64 |
| **Employment status** |  |  |
| Employed | 181 | 32.32 |
| Unemployed | 379 | 67.68 |
| **Marital status** |  |  |
| Married/divorced etc | 97 | 17.29 |
| Single | 464 | 82.71 |
| **Religion** |  |  |
| Christian | 196 | 35.00 |
| Moslem | 364 | 65.00 |
| **Ethnicity** |  |  |
| Mende | 155 | 27.63 |
| Temne | 166 | 29.59 |
| Others | 240 | 42.78 |
| **District** |  |  |
| Bombali District | 89 | 15.84 |
| Port Loko | 64 | 11.39 |
| Kenema | 84 | 14.95 |
| Kono | 68 | 12.10 |
| Bo | 75 | 13.35 |
| Western Area Urban | 132 | 23.49 |
| Wester Area Rural | 50 | 8.90 |
| **Province** |  |  |
| Northern | 153 | 27.22 |
| Eastern | 152 | 27.05 |
| Southern | 75 | 13.35 |
| Western | 182 | 32.38 |

JHS: Junior High School, SHS: Senior High School

**Supplementary Table 3: Socio-demographic characteristics of the transgender population in Sierra Leone**

| **Socio-demographic Factors** | **Frequency** | **Percent** |
| --- | --- | --- |
| **Age in years** |  |  |
| 18-24 years | 421 | 75.04 |
| 25 -29 | 96 | 17.11 |
| 30 or more | 44 | 7.84 |
| **Sex at birth** |  |  |
| Male | 532 | 94.66 |
| Female | 30 | 5.34 |
| **Employment Status** |  |  |
| Employed | 118 | 22.87 |
| Unemployed | 398 | 77.13 |
| **Marital Status** |  |  |
| Never married | 542 | 96.44 |
| Other | 20 | 3.56 |
| **Level of Education** |  |  |
| No education | 46 | 8.19 |
| Primary | 22 | 3.19 |
| Secondary | 382 | 67.97 |
| Higher | 112 | 19.93 |
| **Place of sleep** |  |  |
| Yes | 545 | 96.98 |
| No | 17 | 3.02 |
| **Income** |  |  |
| less than 500000 \ | 455 | 80.96 |
| 5000000+ | 107 | 19.04 |
| **Number of parity** |  |  |
| No child | 379 | 70.19 |
| 1-2 children | 115 | 21.30 |
| 3 or more | 46 | 8.52 |
| **Ethnicity** |  |  |
| Mende | 203 | 36.12 |
| Temme | 168 | 29.89 |
| Others | 191 | 33.99 |
| **Religion** |  |  |
| Muslim | 204 | 36.43 |
| Christian | 356 | 63.57 |
| **Regions** |  |  |
| Northern | 141 | 25.09 |
| Eastern | 118 | 21.00 |
| Southern | 93 | 16.55 |
| Western | 210 | 37.37 |
| **District** |  |  |
| Bombali | 88 | 15.66 |
| Port Loko | 53 | 9.43 |
| Kenema | 96 | 17.08 |
| Kono | 22 | 3.91 |
| Bo | 93 | 16.55 |
| Western Area Urban | 145 | 25.80 |
| Western Area Rural | 65 | 11.57 |

**Supplementary Table 4: Sociodemographic characteristics of PWID in the six regional headquarter towns**

| **Sociodemographic characteristics** | **Frequency** | **Percent** |
| --- | --- | --- |
| **Age in years** |  |  |
| 18-24 years | 560 | 48.53 |
| 25-29 years | 337 | 29.20 |
| 30+ | 257 | 22.27 |
| **Sex at birth** |  |  |
| Male | 1070 | 92.72 |
| Female | 84 | 7.28 |
| **Education level** |  |  |
| None/primary | 193 | 16.74 |
| JHS | 254 | 22.03 |
| SHS/Tech | 626 | 54.29 |
| Higher | 80 | 6.94 |
| **Income** |  |  |
| <500000 | 385 | 72.78 |
| 500000+ | 144 | 27.22 |
| **Employment status** |  |  |
| Employed | 543 | 47.14 |
| Unemployed | 609 | 52.86 |
| **Marital status** |  |  |
| Married/divorced etc | 432 | 37.44 |
| Single | 722 | 62.56 |
| **Religion** |  |  |
| Christian | 366 | 31.72 |
| Moslem | 788 | 68.28 |
| **Ethnicity** |  |  |
| Mende | 307 | 26.63 |
| Temne | 398 | 34.52 |
| Others | 448 | 38.86 |
| **District** |  |  |
| Bombali District | 159 | 13.77 |
| Port Loko | 172 | 14.89 |
| Kenema | 150 | 12.99 |
| Kono | 133 | 11.52 |
| Bo | 158 | 13.68 |
| Western Area Urban | 273 | 23.64 |
| Wester Area Rural | 110 | 9.52 |
| **Province** |  |  |
| Northern | 331 | 28.66 |
| Eastern | 283 | 24.50 |
| Southern | 158 | 13.68 |
| Western | 383 | 33.16 |

JHS: Junior High School, SHS: Senior High School

**Supplementary Table 5: Characteristics of Prisoners in Sierra Leone**

|  | **Frequency** | **Percent** |
| --- | --- | --- |
| **Nationality** |  |  |
| Sierra Leone | 441 | 96.71 |
| Liberia | 4 | 0.88 |
| Guinea | 9 | 1.97 |
| Other countries | 2 | 0.44 |
| **Age in years** |  |  |
| **Mean (SD)** | 32.39 (10.72) |  |
| 18-24years | 108 | 23.68 |
| 25-29years | 126 | 27.63 |
| 30-39years | 127 | 27.85 |
| 40+years | 95 | 20.83 |
| **Sex at birth** |  |  |
| Male | 390 | 85.53 |
| Female | 66 | 14.47 |
| **Current gender** |  |  |
| Male | 391 | 85.75 |
| Female | 65 | 14.25 |
| **Ever attended school** |  |  |
| Yes | 334 | 73.25 |
| No | 122 | 26.75 |
| **Highest level of education** |  |  |
| Primary | 91 | 27.25 |
| JSS | 115 | 34.43 |
| SSS/ Technical Vocational | 109 | 32.63 |
| Higher | 19 | 5.69 |
| **Ever married/ Cohabited** |  |  |
| Yes | 293 | 64.25 |
| No | 163 | 35.75 |
| **What is your marital status at the moment?** |  |  |
| Currently Married | 96 | 32.76 |
| Separated | 110 | 37.54 |
| Divorced | 26 | 8.87 |
| Widowed | 17 | 5.8 |
| Cohabitating | 15 | 5.12 |
| Single | 25 | 8.53 |
| No Response | 4 | 1.37 |
| **Religion** |  |  |
| Christian | 180 | 39.47 |
| Moslem | 275 | 60.31 |
| No Religion | 1 | 0.22 |
| **Province** |  |  |
| Northern Province | 40 | 8.77 |
| Eastern Province | 68 | 14.91 |
| Southern Province | 23 | 5.04 |
| Western Area | 325 | 71.27 |
| **District** |  |  |
| Bombali District | 22 | 4.82 |
| Port Loko | 18 | 3.95 |
| Kenema | 37 | 8.11 |
| Kono | 31 | 6.8 |
| Bo | 23 | 5.04 |
| Western Area Urban | 294 | 64.47 |
| Western Area Rural | 31 | 6.8 |
| **Town name** |  |  |
| Makeni | 22 | 4.82 |
| Port Loko | 18 | 3.95 |
| Kenema | 37 | 8.11 |
| Koidu | 31 | 6.8 |
| Bo | 23 | 5.04 |
| Freetown | 294 | 64.47 |
| Waterloo | 31 | 6.8 |
| **Venue** |  |  |
| Makeni Male Correctional Center | 22 | 4.82 |
| Port Loko Correctional Center | 18 | 3.95 |
| Kenema Male Correctional Center | 37 | 8.11 |
| Sefadu Correctional Center | 31 | 6.8 |
| Bo Correctional Center | 23 | 5.04 |
| Freetown Male Correctional Center | 234 | 51.32 |
| Waterloo Simulation Correctional Center | 31 | 6.8 |
| Freetown Female Correctional Center | 60 | 13.16 |
| **Type of respondent** |  |  |
| Remand Prisoners | 60 | 13.16 |
| Convicted Prisoners | 358 | 78.51 |
| Other | 38 | 8.33 |

Abbreviation: SD: Standard deviation

**Supplementary Table 6: HIV prevalence among female sex workers by sociodemographic characteristics**

|  | HIV status | Unadjusted prevalence ratio from Modified Poisson Regression Model | Unadjusted Odds ratio from the binary logistic regression model |
| --- | --- | --- | --- |
| **Sociodemographic** | HIV+ (%) [95% CI] | uPR [95% CI] | uOR [95% CI] |
| **Age in years** |  |  |  |
| 18-24 years | 10.47 [6.60-16.21] | 1 | 1 |
| 25-29 years | 12.89 [8.82-18.48 | 1.23 [0.91-1.66] | 1.27 [0.90-1.77] |
| 30-39 years | 14.62 [8.90-23.09] | 1.40 [0.98-1.99] | 1.47 [0.97-2.21] |
| 40+ years | 4.35 [1.00-16.98] | 0.42 [0.09-1.84] | 0.39 [0.08-1.86] |
| **Highest level of education** |  |  |  |
| None | 11.25 [7.56-16.43] | 1 | 1 |
| Primary | 17.16 [8.47-31.67] | 1.52 [1.03-2.26]* | 1.63 [1.00-2.64]* |
| JSS | 11.73 [8.15-16.59] | 1.04 [0.88-1.23] | 1.05 [0.87-1.26] |
| SSS/ Technical Vocational | 9.49 [6.96-12.81] | 0.84 [0.64-1.10] | 0.83 [0.61-1.12] |
| Higher | 7.14 [0.91-39.31] | 0.63 [0.09-4.29] | 0.61 [0.07-5.41] |
| **Current employment status** |  |  |  |
| Employed | 13.34 [10.91-16.21] | 1 | 1 |
| Unemployed | 10.60 [8.63-12.96] | 0.79 [0.46-1.36] | 0.77 [0.42-1.42] |
| **Household income categorized** |  |  |  |
| < 50 USD | 10.22 [6.78-15.12] | 1 | 1 |
| 50 USD or more | 16.56 [10.86-24.42] | 1.62 [1.17-2.24]** | 1.74 [1.19-2.54]** |
| **Marital status at the moment** |  |  |  |
| Never married or cohabited | 9.90 [6.54-14.70] | 1 | 1 |
| Others | 17.07 [13.57-21.26] | 1.73 [1.36-2.19]*** | 1.87 [1.42-2.48]*** |
| **Religion** |  |  |  |
| Christian | 11.31 [6.89-18.02] | 1 | 1 |
| Moslem | 11.99 [8.37-16.88] | 1.06 [0.81-1.38] | 1.07 [0.79-1.44] |
| **Regions** |  |  |  |
| Northern | 8.26 [5.35-12.55] | 1 | 1 |
| Eastern | 8.33 [5.08-13.39] | 1.01 [0.56-1.83] | 1.01 [0.53-1.93] |
| Southern | 11.76 [5.51-23.37] | 1.42 [0.63-3.21] | 1.48 [0.60-3.68] |
| Western | 16.00 [10.13-24.35] | 1.94 [1.07-3.49]*** | 2.12 [1.08-4.15]*** |
| **District** |  |  |  |
| Bombali | 5.38 [2.99-9.50] | 1 | 1 |
| Port Loko | 11.11 [5.90-19.95] | 2.06 [0.93-4.60] | 2.20 [0.91-5.28] |
| Kenema | 8.33 [5.08-13.39] | 1.55 [0.75-3.21] | 1.60 [0.73-3.48] |
| Bo | 11.76 [5.51-23.37] | 2.19 [0.89-5.35] | 2.34 [0.87-6.30]* |
| Western Urban | 20.88 [17.60-24.58] | 3.88 [2.17-6.92]*** | 4.46 [2.49-8.64]*** |
| Western Rural | 4.32 [2.57-7.17] | 0.80 [00.38-1.69] | 0.79 [0.36-1.74] |
| **Town/City name** |  |  |  |
| Makeni | 5.38 [2.99-9.50] | 1 | 1 |
| Lingui | 11.11 [5.90-19.95] | 2.06[0.93-4.60] | 2.20[10.91-5.28] |
| Kenema | 8.33 [5.08-13.39] | 1.55 [0.75-3.21] | 1.60 [0.73-3.48] |
| Bo | 11.76 [5.51-23.37] | 2.19 [0.89-5.35] | 2.34 [0.87-6.30] |
| Freetown | 20.88 [17.60-24.58] | 3.88 [2.17-6.92]*** | 4.64 [2.49-8.64]*** |
| Waterloo and Grafton | 4.32 [2.57-7.17] | 0.80 [0.38-1.69] | 0.79 [0.36-1.74] |
| **Ethnic affiliation** |  |  |  |
| Mende | 11.52 [7.06-18.22] | 1 | 1 |
| Temne | 10.15 [6.70-15.11] | 0.88 [0.53-1.46] | 0.87 [0.49-1.53] |
| Limba | 10.95 [5.42-20.88] | 0.95 [0.50-1.82] | 0.94 [0.46-1.96] |
| Others (Mandingo, Kono, Krio, Kuranko, etc) | 14.49 [8.74-23.08] | 1.26 [0.87-1.82] | 1.30 [0.85-1.99] |
| **Type of FSW** |  |  |  |
| Roamer | 12.35 [8.12-18.34] | 1 | 1 |
| Seater | 10.93 [6.48-17.84] | 0.88 [0.56-1.41] | 0.87 [0.51-1.47] |

Abbrevaiation: FSW: Female Sex Workers, uPR: Unadjusted Prevalence Ratio, our: Unadjusted odds ratio; CI: Confidence interval. P-value notation: ***p<0.001, **p<0.01, *p<0.05. JHS: Junior High School, SHS: Senior High School

**Supplementary Table 7: HIV prevalence among men who have sex with men by sociodemographic characteristics**

|  | Crude/unadjusted estimate of HIV prevalence | RDS design-based weighted estimated | Unadjusted prevalence ratio from double selection Lasso Poisson Regression Model adjusting for sampling weight from RDS | Unadjusted Odds ratio from Firth Penalized maximum likelihood logistic regression model adjusting for sampling weight from RDS |
| --- | --- | --- | --- | --- |
| Sociodemographic characteristics | HIV+ (%) [95% CI] | HIV+ (%) [95% CI] | uPR [95% CI] | uOR [95% CI] |
| **Age in years** |  |  |  |  |
| 17-24 years | 1.15 [0.43-3.03] | 1.35 [0.42-4.26] | 1 | 1 |
| 25-29 years | 6.25 [3.15-12.02] | 5.94 [2.66-12.73] | 5.44 [1.66-17.77]** | 5.36 [1.68-17.10]** |
| 30+ | 7.06 [3.20-14.86] | 7.63 [2.77-19.35] | 6.14 [1.77-21.30]** | 6.23 [1.83-21.20]** |
| **Education level** |  |  |  |  |
| None/primary | 2.04 [0.29-13.16] | 0.45 [0.06-3.22] | 1 | 1 |
| JHS | 5.88 [2.22-14.68] | 6.89 [2.26-19.13] | 2.88 [0.33-25.05] | 2.24 [0.34-14.72] |
| SHS/Tech | 2.56 [1.38-4.69] | 2.51 [1.15-5.37] | 1.25 [0.16-9.60] | 0.89 [0.16-5.05] |
| Higher | 5.66 [1.83-16.17] | 7.74 [2.25-23.48] | 2.77 [0.30-25.83] | 2.23 [0.32-15.69] |
| **Income** |  |  |  |  |
| **Less tha 500000 Leone** | 1.72 [0.82-3.57] | 1.50 [0.58-3.81] | 1 | 1 |
| 50000 Leone or higher | 6.35 [3.20-12.20] | 6.86 [3.03-14.76] | 3.69 [1.36-9.99]** | 3.80 [1.40-10.36]** |
| **Employment status** |  |  |  |  |
| Employed | 5.52 [2.99-9.97] | 5.53 [2.63-11.23] | 1 | 1 |
| Unemployed | 2.11 [1.06-4.17] | 2.32 [0.99-5.36] | 0.38 [0.15-0.95]* | 0.38 [0.15-0.94]* |
| **Marital status** |  |  |  |  |
| Married/divorced etc | 7.22 [3.47-14.39] | 7.57 [2.91-18.31] | 1 | 1 |
| Single | 2.37 [1.32-4.23] | 2.54 [1.28-5.00] | 0.33 [0.13-0.83]* | 0.30 [0.12-0.78]* |
| **Religion** |  |  |  |  |
| Christian | 6.63 [3.89-11.10] | 7.54 [3.91-14.04] | 1 | 1 |
| Moslem | 1.37 [0.57-3.26] | 1.37 [0.48-3.81] | 0.21 [0.07-0.57]** | 0.20 [0.07-0.56]** |
| **Ethnicity** |  |  |  |  |
| Mende | 1.94 [0.62-5.84] | 0.93 [0.27-3.17] | 1 | 1 |
| Temne | 1.20 [0.30-4.70] | 1.52 [0.23-9.21] | 0.62 [0.11-3.68] | 0.67 [0.13-3.47] |
| Others | 5.42 [3.17-9.11] | 6.30 [3.38-11.46] | 2.80 [0.81-9.67] | 2.59 [0.79-8.58] |
| **District** |  |  |  |  |
| Bombali District | 1.12 [0.16-7.57] | 0.14 [0.02-1.04] | 1 | 1 |
| Port Loko | No observation | No observation | No observation | No observation |
| Kenema | 1.19 [0.17-8.00] | 0.80 [0.11-5.55] | 1.06 [0.07-16.68] | 1.07 [0.11-10.50] |
| Kono | No observation | No observation | No observation | 0.43 [0.02-10.73] |
| Bo | 1.33 [0.19-8.89] | 1.40 [0.20-9.38] | 1.19 [0.08-18.67] | 1.19 [0.12-11.73] |
| Western Area Urban | 11.36 [6.96-18.01] | 11.98 [6.63-20.68] | 10.11 [1.36-75.20]* | 7.77 [1.41-42.62]* |
| Wester Area Rural | No observation | No observation | No observation | 0.58 [0.02-14.54] |
| **Province** |  |  |  |  |
| Northern | 0.65 [0.09-4.51] | 0.08 [0.01-0.60] | 1 | 1 |
| Eastern | 0.66 [0.09-4.54] | 0.47 [0.07-3.29] | 1.01 [0.06-15.99] | 0.99 [0.10-9.63] |
| Southern | 1.33 [0.19-8.89] | 1.40 [0.20-9.38] | 2.04 [0.13-32.25] | 2.00 [0.20-19.65] |
| Western | 8.24 [5.02-13.23] | 9.24 [5.10-16.17] | 12.61 [1.68-94.54]** | 9.26 [1.71-50.22]** |

Abbreviation: MSM: Men who have sex with men, uPR: Unadjusted Prevalence Ratio, our: Unadjusted odds ratio; CI: Confidence interval. P-value notation: ***p<0.001, **p<0.01, *p<0.05. JHS: Junior High School, SHS: Senior High School

**Supplementary Table 8:** HIV prevalence among TGs by sociodemographic characteristics

|  | HIV status | |  |  |
| --- | --- | --- | --- | --- |
| Sociodemographic factors | Unadjusted estimate | RDS design-based weighted estimated | Unadjusted prevalence ratio from Modified Poisson Regression Model adjusting for sampling weight from RDS | Unadjusted Odds ratio from the binary logistic regression model adjusting for sampling weight from RDS |
|  | HIV+ (%) [95% CI] | HIV+ (%) [95% CI] | uPR [95% CI] | uOR [95% CI] |
| Age in years |  |  |  |  |
| 18-24 | 6.65 [4.63-9.47] | 4.14 [2.69-6.32] | 1 | 1 |
| 25-29 | 10.42 [5.69-18.31] | 5.34 [2.37-11.63] | 1.29 [0.52-3.20] | 1.31 [0.50-3.40] |
| 30 or more | 6.82 [2.21-19.15] | 2.67 [0.77-8.84] | 0.65 [0.18-2.36] | 0.64 [0.17-2.42] |
| Sex at birth |  |  |  |  |
| Male | 7.52 [5.56-10.10] | 4.36 [3.01-6.29] | 1 | 1 |
| Female | 3.33 [0.47-20.27] | 1.11 [0.15-7.79] | 0.25 [0.03 – 1.93] | 0.05[0.03-1.91] |
| Employment status |  |  |  |  |
| employed | 13.56[8.47 – 21.01] | 8.97[4.95 -15.71] | 1 | 1 |
| unemployed | 5.78[3.87-8.55] | 3.34[2.05-5.41] | 0.37 [0.18-0.79] | 0.35[0.16-0.79] |
| Marital Status |  |  |  |  |
| never married | 6.83[4.98-9.29] | 4.05[2.75-5.93] | 1 | 1 |
| other | 20.00[7.70-42.84] | 8.72[2.70-24.76] | 2.15[0.65-7.08] | 0.04[0.03-0.06] |
| Level of education |  |  |  |  |
| no education | 4.35[1.09-15.83] | 1.68[0.40-6.77] | 1 | 1 |
| Primary education | 4.55[0.63-26.23] | 1.24[0.16-8.72] | 0.74[0.06-8.59] | 0.73[0.06-8.85 |
| secondary education | 7.07[4.89-10.12] | 4.37[2.79-6.78] | 2.60[0.59-11.54] | 2.68[0.59-12.21] |
| Higher education | 9.82[5.52-16.89] | 5.55[2.69-11.12] | 3.31[0.67-16.22] | 3.44[0.67-17.58] |
| Place of sleep |  |  |  |  |
| Yes | 7.16 [ 5.27 - 9.65] | 4.17 [ 2.86 -6.03] | 1 | 1 |
| No | 11.76[ 2.95-36.90] | 5.83[0.97-28.17] | 1.40[0.24-8.24] | 1.42 [0.22-9.35] |
| Monthly income (Leone) |  |  |  |  |
| Less than 500000 | 5.71[ 3.92- 8.27] | 3.15[1.97- 4.99] | 1 | 1 |
| 5000000 or higher | 14.02[8.62- 21.98] | 8.86 [4.86-15.64] | 2.81[1.33- 5.95]*** | 2.99[1.34-6.67]*** |
| Parity |  |  |  |  |
| No child | 4.22 [2.60- 6.79] | 2.73[1.57-4.70] | 1 | 1 |
| 1-2 children | 9.57[5.37- 16.47] | 4.61[2.18-9.48] | 1.69[0.68-4.22] | 1.72[0.66-4.47] |
| 3 or more | 28.26[17.15-42.84] | 17.79[8.95- 32.26] | 6.51[2.79-15.19]*** | 7.71[2.93-20.26]*** |
| Religion |  |  |  |  |
| Christian | 10.78[7.20-15.85] | 5.44[3.32- 8.79] | 1 | 1 |
| Moslem | 5.34[3.43-8.22] | 3.60[2.12-6.07] | 0.66[0.32-1.36] | 0.65[0.31-1.38] |
| Ethnicity |  |  |  |  |
| Mende | 4.43[2.32 -8.31] | 1.93 [0.84- 4.38] | 1 | 1 |
| Temne | 8.93[5.45-14.30] | 5.56 [3.11-9.76] | 2.88[1.05-7.87]** | 2.99 [1.06 -8.44]** |
| others | 8.90[ 5.60-13.86] | 5.82[3.28 -10.13] | 3.01[1.11-8.19]*** | 3.14[1.12-8.82]*** |
| Region |  |  |  |  |
| Northern | 5.67[2.86 -10.95] | 3.59 [1.74 - 7.24] | 1 | 1 |
| Eastern | 2.54[0.82-7.60] | 1.61[0.41 - 6.19] | 0.45[0.10-2.10] | 0.44[0.09-2.13] |
| Southern | 5.38[2.25-12.29] | 2.18[0.83 - 5.56] | 0.61[ 0.19 -1.99] | 0.60[0.18-2.03] |
| Western | 11.90[8.17 - 17.04] | 7.18[4.42 - 11.45] | 2.00[0.85-4.72] | 2.08[0.85-5.11] |
| District |  |  |  |  |
| Bombali | 9.09[4.60 -17.16 | 5.09[2.46-10.23] | 1 | 1 |
| Kenema | 3.13[1.01-9.26] | 1.93[0.48-7.36] | 0.38[0.08-1.77] | 0.37[0.08-1.79] |
| Bo | 5.38[2.25 - 12.29] | 2.18[0.83- 5.56] | 0.43[0.13-1.40] | 0.42[0.12-1.42] |
| Western Area Urban | 8.28[4.75 - 14.02] | 4.31[2.13 -8.53] | 0.85[0.31-2.30] | 0.84[0.29-2.39] |
| Western Area Rural | 20.00[11.97-31.49] | 13.30[6.83-24.29] | 2.61[1.00-6.82]** | 2.86[1.00-8.21]** |

Abbreviation: TG: Transgender, uPR: Unadjusted Prevalence Ratio, our: Unadjusted odds ratio; CI: Confidence interval. P-value notation: ***p<0.001, **p<0.01, *p<0.05.

**Supplementary Table 9: HIV prevalence among PWID by sociodemographic characteristics**

|  | Crude/unadjusted estimate of HIV prevalence | RDS design-based weighted estimated | Unadjusted prevalence ratio from double selection Lasso Poisson Regression Model adjusting for sampling weight from RDS | Unadjusted Odds ratio from Firth Penalized maximum likelihood logistic regression model adjusting for sampling weight from RDS |
| --- | --- | --- | --- | --- |
| **Sociodemographic characteristics** | HIV+ (%) [95% CI] | HIV+ (%) [95% CI] | uPR [95% CI] | uOR [95% CI] |
| **Age in years** |  |  |  |  |
| 18-24 years | 3.39[2.17- 5.26] | 4.72[2.51- 8.70] | 1.00 | 1.00 |
| 25-29 years | 1.19[0.45-3.12] | 1.76[0.56 -5.41] | 0.35[0.12-1.02] | 0.40[0.14- 1.12] |
| 30+ | 5.45[3.25- 8.99] | 5.76[3.06- 10.59] | 1.61 [0.82- 3.15] | 1.74[ 0.86-3.51] |
| **Sex at birth** |  |  |  |  |
| Male | 2.15[1.43 -3.22] | 2.89[1.68- 4.90] | 1.00 | 1.00 |
| Female | 16.67[10.12- 26.21] | 16.91[8.10-31.99] | 7.40[3.89 - 14.08]*** | 8.71[4.32- 17.56]*** |
| **Education level** |  |  |  |  |
| None/primary | 5.70[3.18 - 10.00] | 7.06[3.28-14.53] | 1.00 | 1.00 |
| JHS | 5.12[2.99-8.62] | 6.04[2.86- 12.30] | 0.88[0.40- 1.93] | 0.87[0.39-1.97] |
| SHS/Tech | 1.44 [0.75- 2.74] | 1.56[0.53- 4.49] | 0.26[0.11- 0.62]* | 0.25[0.11-0.61]* |
| Higher | 5.00[1.89 -12.59] | 9.55[3.25- 24.93] | 0.93[0.30 - 2.87] | 0.10[0.32- 3.08] |
| **Income** |  |  |  |  |
| <500000 | 3.64[2.16 - 6.05] | 5.98[3.07- 11.34] | 1.00 | 1.00 |
| 500000+ | 3.47[1.45 - 8.09] | 3.31[1.04 -10.02] | 0.95[0.35-2.61] | 1.12[0.41-3.07] |
| **Employment status** |  |  |  |  |
| Employed | 3.50[2.24- 5.42] | 4.92[2.77- 8.57] | 1.00 |  |
| Unemployed | 2.96[1.87 -4.64] | 3.47[1.74- 6.78] | 0.84[0.45 -1.59] | 0.85[0.44- 1.62] |
| **Marital status** |  |  |  |  |
| Married/divorced etc | 4.40[2.82 -6.79] | 4.85[2.58- 8.93] | 1.00 | 1.00 |
| Single | 2.49 [ 1.58- 3.92] | 3.79[2.07- 6.83] | 0.57[0.30-1.07] | 0.54[0.28- 1.03] |
| **Religion** |  |  |  |  |
| Christian | 3.83[2.28-6.36] | 4.28[2.07- 8.65] | 1.00 | 1.00 |
| Moslem | 2.92[1.95-4.36] | 4.09[2.36-7.01] | 0.76[0.40- 1.47] | 0.74[0.38- 1.44] |
| **Ethnicity** |  |  |  |  |
| Mende | 2.93 [1.53- 5.54] | 2.36[1.03- 5.33] | 1.00 | 1.00 |
| Temne | 2.26[1.18-4.29] | 3.54[1.57- 7.80] | 0.77[0.31- 1.92] | 0 .76[0.30-1.89] |
| Others | 4.24[2.72- 6.56] | 5.90[3.16- 10.74 | 1.45[0.66- 3.16] | 1.42[0.65- 3.13] |
| **District** |  |  |  |  |
| Bombali District | 7.55[4.33-12.83] | 9.61[4.74- 18.51] | 1.00 | 1.00 |
| Port Loko | 1.74[0.56 - 5.27] | 1.49 [0.31-6.89] | 0.30[0.08- 1.09] | 0 .33[0.09-1.14] |
| Kenema | 4.67[2.24 - 9.47] | 5.03[2.00 -12.09] | 0.72[0.29-1.81] | 0.73[0.28-1.90] |
| Bo | 0.63 [0.09- 4.36] | 1.15[0.16-7.73] | 0.09[0.01- 0.68]* | 0.12[0.02-0.66]* |
| Western Area Urban | 4.76[2.78 -8.03] | 5.50 [2.53-11.56] | 0.72[0.33-1.57] | 0.71[0.31- 1.61] |
| Wester Area Rural | 0.91[0.13- 6.18] | 2.81[0.40- 17.33] | 0.15[0.02- 1.18] | 0.20[0.04-1.13] |
| **Province** |  |  |  |  |
| Northern | 4.53[2.75 -7.38] | 7.66[3.88- 14.57] | 1.00 |  |
| Eastern | 2.47[1.18 - 5.10] | 1.91 [0.76- 4.74] | 0.50[0.20- 1.25] | 0 .51[0.21-1.23] |
| Southern | 0.63[0.09-4.36] | 1.15[0.16-7.73] | 0.13[0.02-0.96]* | 0.178[0.03- 0.96]* |
| Western | 3.66[2.18 -6.08] | 4.88[2.37-9.77] | 0.83[0.41- 1.69] | 0.83[0.40-1.72] |

Abbreviation: PWID: People who inject drugs, uPR: Unadjusted Prevalence Ratio, our: Unadjusted odds ratio; CI: Confidence interval. P-value notation: ***p<0.001, **p<0.01, *p<0.05.

**Supplementary Table 10:** Prevalence and sociodemographic determinants of HIV among Prisoners in Sierra Leone

|  | HIV status | Unadjusted prevalence ratio from Modified Poisson Regression Model | Unadjusted Odds ratio from the binary logistic regression model |
| --- | --- | --- | --- |
|  | HIV+ (%) [95% CI] | uPR [95% CI] | uOR [95% CI] |
| **Overall** | 3.73[1.40 – 9.57] |  |  |
| Age |  |  |  |
| 18-24years | 6.48[3.12 - 13] | 1 | 1 |
| 25-29years | 0.79[0.11 - 5.44] | 0.12[0.02 - 1] | 0.12[0.01 - 0.95] |
| 30-39years | 3.15[1.18 - 8.11] | 0.49[0.14 - 1.66] | 0.47[0.13 - 1.65] |
| 40+years | 5.26[2.2 - 12.05] | 0.81[0.26 - 2.56] | 0.8[0.25 - 2.61] |
| **Marital status** |  |  |  |
| Never married/cohabited | 4.49[2.72 - 7.32] | 1 | 1 |
| Others | 1.64[0.41 - 6.34] | 0.37[0.08 - 1.6] | 0.35[0.08 - 1.57] |
| **Highest level of education** |  |  |  |
| None | 1.64[0.41 - 6.34] | 1 | 1 |
| Primary | 4.4[1.66 - 11.16] | 2.68[0.49 - 14.64] | 2.76[0.49 - 15.4] |
| JSS | 3.48[1.31 - 8.92] | 2.12[0.39 - 11.58] | 2.16[0.39 - 12.04] |
| SSS/ Technical Vocational/ higher | 5.47[2.62 - 11.05] | 3.34[0.69 - 16.06] | 3.47[0.71 - 17.05] |
| **Marital status** |  |  |  |
| Never married/cohabited | 3.07[1.6 - 5.81] | 1 | 1 |
| Others | 4.91[2.47 - 9.52] | 1.6[0.62 - 4.14] | 1.63[0.62 - 4.31] |
| Sex at birth |  |  |  |
| Male | 2.31[1.2 - 4.38] | 1 | 1 |
| Female | 12.12[6.17 - 22.45] | 5.25[2.03 - 13.61]* | 5.84[2.17 - 15.74]* |
| **Religion** |  |  |  |
| Christian | 4.44[2.23 - 8.65] | 1 | 1 |
| Moslem | 3.27[1.71 - 6.18] | 0.74[0.28 - 1.91] | 0.73[0.28 - 1.92] |
| **Regions** |  |  |  |
| Northern | 2.5[0.35 - 15.8] | 1 | 1 |
| Eastern | 4.41[1.43 - 12.84] | 1.76[0.18 - 16.97] | 1.8[0.18 - 17.91] |
| Southern | 4.35[0.61 - 25.32] | 1.74[0.11 - 27.8] | 1.77[0.11 - 29.76] |
| Western | 3.69[2.11 - 6.4] | 1.48[0.19 - 11.36] | 1.5[0.19 - 11.81] |
| **District** |  |  |  |
| Bombali | 4.55[0.63 - 26.25] | 1 | 1 |
| Kenema | 8.11[2.63 - 22.37] | 1.78[0.19 - 17.15] | 1.85[0.18 - 19] |
| Bo | 4.35[0.61 - 25.32] | 0.96[0.06 - 15.29] | 0.95[0.06 - 16.27] |
| Western Urban | 4.08[2.33 - 7.06] | 0.9[0.12 - 6.91] | 0.89[0.11 - 7.21] |

AbbreviationuPR: Unadjusted Prevalence Ratio, our: Unadjusted odds ratio; CI: Confidence interval. P-value notation: ***p<0.001, **p<0.01, *p<0.05.

Female Sex Workers (FSW)

Sociodemographic Characteristics of FSW in Sierra Leone

This section presents the characteristics of FSWs in the six regional headquarter towns in Sierra Leone. Overall, 1430 FSWs were enrolled from 81 hotspots with an acceptance rate of 100% for both survey interviews and HIV on and off-site laboratory testing. Of the 1430 women, the average age was 24.5 years (SD =5.5, youngest=16 years, oldest=53 years); 774 (54.1%) were aged 16-24 years and 1039 (72.3%) had ever attended school. Approximately 1061 (74.2%) of the FSW had never been married nor cohabited (Table 1). About half of the FSWs (n = 720, 50.4%) earn less than 500000 Leon (50 USD equivalent) per month, 826 (57.8%) were roamers and 604 (42.2%) were seaters. The Western urban areas had the highest number of FSWs (n=388, 27.1%) with the majority operating in Bars (n=483, 33.8%). The detailed distribution of the sociodemographic characteristics of FSWs can be found in Table 1.

Table 11: Characteristics of the Female Sex Workers in Sierra Leone

| **Characteristics of FSWs in Sierra Leone** |  |  |
| --- | --- | --- |
| **Nationality** | Freq | Percent |
| Sierra Leone | 1411 | 98.67 |
| Liberia | 9 | 0.63 |
| Guinea | 7 | 0.49 |
| Other countries | 3 | 0.21 |
| **Mean age (SD)** | 24.89 (5.53) |  |
| **Age in years** |  |  |
| 18-24 years | 774 | 54.13 |
| 25-29 years | 380 | 26.57 |
| 30-39 years | 253 | 17.69 |
| 40+ years | 23 | 1.61 |
| **Ever attended school** |  |  |
| Yes | 1039 | 72.66 |
| No | 391 | 27.34 |
| **Highest level of education** |  |  |
| Primary | 204 | 19.63 |
| JSS | 452 | 43.50 |
| SSS/ Technical Vocational | 369 | 35.51 |
| Higher | 14 | 1.35 |
| **Currently, a student or enrolled as a learner** |  |  |
| Yes | 218 | 20.98 |
| No | 821 | 79.02 |
| **Current employment status** |  |  |
| Employed Full-Time | 406 | 28.39 |
| Employed Part-Time | 231 | 16.15 |
| Full-Time Student | 63 | 4.41 |
| Retired | 5 | 0.35 |
| Unemployed | 657 | 45.94 |
| Other | 58 | 4.06 |
| Don’t Know | 6 | 0.42 |
| No Response | 4 | 0.28 |
| **Median household income (LQ, UP)** | 200000 [600, 500000] |  |
| **Household income categorized** |  |  |
| **<500000** | 720 | 50.35 |
| 50000+ | 302 | 21.12 |
| Missing | 408 | 28.53 |
| **Ever been married or Cohabited with a man** |  |  |
| Yes | 369 | 25.80 |
| No | 1061 | 74.20 |
| **Marital status at the moment** |  |  |
| Currently Married | 16 | 4.34 |
| Separated | 130 | 35.23 |
| Divorced | 32 | 8.67 |
| Widowed | 19 | 5.15 |
| Cohabitating | 97 | 26.29 |
| Single | 75 | 20.33 |
| **Religion** |  |  |
| Christian | 566 | 39.58 |
| Moslem | 859 | 60.07 |
| Traditional | 2 | 0.14 |
| No Religion | 3 | 0.21 |
| **Regions** |  |  |
| Northern | 448 | 31.33 |
| Eastern | 228 | 15.94 |
| Southern | 204 | 14.27 |
| Western | 550 | 38.46 |
| **District** |  |  |
| Bombali | 223 | 15.59 |
| Port Loko | 225 | 15.73 |
| Kenema | 228 | 15.94 |
| Bo | 204 | 14.27 |
| Western Urban | 388 | 27.13 |
| Western Rural | 162 | 11.33 |
| **Town/City name** |  |  |
| Makeni | 223 | 15.59 |
| Lingui | 225 | 15.73 |
| Kenema | 228 | 15.94 |
| Bo | 204 | 14.27 |
| Freetown | 388 | 27.13 |
| Waterloo | 63 | 4.41 |
| Grafton | 99 | 6.92 |
| **Ethnic affiliation** |  |  |
| Mende | 495 | 34.62 |
| Temne | 453 | 31.68 |
| Fula | 46 | 3.22 |
| Limba | 137 | 9.58 |
| Mandingo | 85 | 5.94 |
| Kono | 30 | 2.10 |
| Krio | 45 | 3.15 |
| Kuranko | 13 | 0.91 |
| Other (Specify) | 126 | 8.81 |
| **Type of venue** |  |  |
| Bar | 483 | 33.78 |
| Brothels | 221 | 15.45 |
| Night Club | 34 | 2.38 |
| Drinking Joint | 111 | 7.76 |
| Hotel | 30 | 2.10 |
| Private Home Parties | 40 | 2.80 |
| Resthouse | 272 | 19.02 |
| Rest Stop | 2 | 0.14 |
| Street | 28 | 1.96 |
| Other | 209 | 14.62 |
| **Record type of respondent** |  |  |
| Roamer | 826 | 57.76 |
| Seater | 604 | 42.24 |

Note: Standard Deviation abbreviated as SD

**Sexual history and risk behaviour of FSWs in the six regional headquarter towns**

Table 12 shows the sexual history and risk behaviour of FSWs in Sierra Leone. The average age at first vaginal sex and the age at which FSWs first receive money for sex was 16 and 18 years respectively. The reasons for exchanging sex for money were varied but most (n=803/1430) indicated that they need money. About 44% (n=631) of the FSW did not have vaginal or anal sex with non-paying men in the last three months. For those that had anal or vaginal sex with non-paying men, approximately 41% indicated that the men used condoms.

**Table 12: Sexual history and risk behaviour of FSWs in Sierra Leone**

| **Sexual history and risk behaviour of FSW in Sierra Leone** | Freq | Percent |
| --- | --- | --- |
| **The average age in years at first vaginal sex (SD)** | 15.97 (2.65) |  |
| **Age in years at first vaginal sex** |  |  |
| <18 years | 996 | 69.65 |
| 18 or older | 430 | 30.07 |
| Missing | 4 | 0.28 |
| **Average age first receive money for sex (SD)** | 18.15 (3.71) |  |
| **Age first receive money for sex** |  |  |
| Below 18 | 628 | 43.92 |
| 18+ years | 799 | 55.87 |
| Missing | 3 | 0.21 |
| **Reasons for exchanging sex with money** |  |  |
| Needed money | 803 | 803/1430 |
| Don’t have any other job | 362 | 362/1430 |
| Grew up around people who did sex work | 39 | 39/1430 |
| Forced/pressured into it | 62 | 62/1430 |
| For pleasure | 23 | 23/1430 |
| Encouraged by friends/people I know | 133 | 133/1430 |
| Pays well/you can make a lot of money | 31 | 31/1430 |
| Abandoned by my parents/siblings | 149 | 149/1430 |
| Abandoned by my husband | 82 | 82/1430 |
| Extra money to buy material things I want | 480 | 480/1430 |
| Because I am an orphan/I don't have a mother or father | 358 | 358/1430 |
| Other (specify) | 270 | 270/1430 |
| Don't know | 2 | 2/1430 |
| Decline to answer | 2 | 2/1430 |
| **Number of non-paying men that the FSW had vaginal or anal sex in the last 3 months** |  |  |
| None | 631 | 44.13 |
| One | 246 | 17.20 |
| two | 121 | 8.46 |
| 3 or more | 378 | 26.43 |
| Don't know/Decline | 54 | 3.78 |
| **How many did not use condom in the last 3 months** |  |  |
| All of them used condom | 326 | 40.80 |
| One | 197 | 24.66 |
| 2 or more | 223 | 27.91 |
| Don’t know/decline | 53 | 6.63 |
| **Number men FSW received money in exchange for sex in the last 3 months** |  |  |
| Less than 10 | 520 | 36.36 |
| 10-19 | 287 | 20.07 |
| 20+ | 528 | 36.92 |
| Don't know/decline | 95 | 6.64 |
| **The number of men that FSW had sex without condom in exchanged of money in the last 3 months** |  |  |
| All used condom | 762 | 54.82 |
| Less than 10 | 363 | 26.12 |
| 10 or more | 171 | 12.30 |
| Don't know, Decline | 94 | 6.76 |
| **Median amount of money received after sex (LQ, UQ)** | 25000 (100, 60000) |  |
| Amount of money received after sex |  |  |
| Less than 25000 | 655 | 45.80 |
| 25000 or more | 775 | 54.20 |
| **Any other type of work to receive money beside sex work** |  |  |
| Yes | 358 | 25.03 |
| No | 1066 | 74.55 |
| Decline to answer | 6 | 0.42 |
| **Specific type of work beside sex work** |  |  |
| Street Vendor/Casual Laborer | 130 | 35.71 |
| Professional/Banker/Accountant | 1 | 0.27 |
| Teacher | 1 | 0.27 |
| Business Owner | 86 | 23.63 |
| Hairdresser/Beautician/Masseuse | 84 | 23.08 |
| Waitress/Bartender/Hotel Employee | 6 | 1.65 |
| Tourism/Travel Agent/Tour Guide | 1 | 0.27 |
| Government Worker | 2 | 0.55 |
| Security Guard | 1 | 0.27 |
| Farmer/Agriculture Worker | 1 | 0.27 |
| Other (Specify) | 50 | 13.74 |
| Decline To Answer | 1 | 0.27 |
| **Where FSW normally go for client** |  |  |
| Brothel | 145 | 10.14 |
| Bar, Cafe, Disco, Shebeen, Or Restaurant | 858 | 60.00 |
| Hotel | 51 | 3.57 |
| Street, Park Or Public Transport | 177 | 12.38 |
| Through Friends | 8 | 0.56 |
| Internet (E.G. Facebook), Chat, Or Sms | 8 | 0.56 |
| Party | 1 | 0.07 |
| Service Station | 55 | 3.85 |
| Through an intermediary (Pimp, Bartender, Taxi Driver) | 1 | 0.07 |
| Other | 126 | 8.81 |

Note: Standard Deviation abbreviated as SD

**Condom access and use among FSW in the six regional headquarter towns in Sierra Leone**

Table 13 shows condom access and use among FSW in the six regional headquarter towns in Sierra Leone. More than half (n=988, 69.1%) indicated that condoms are generally affordable and it was very easy to obtain male condoms (n=996, 69.7%). The condoms were mostly secured from a Government hospital/clinic/health center (846/1430).

**Table 13: Condom access and use among FSW in Sierra Leone**

| **Condom access and use among FSW in Sierra Leone** |  |  |
| --- | --- | --- |
| **Affordability of male condoms** | Freq. | Percent |
| Very Affordable | 988 | 69.09 |
| Somewhat Affordable | 335 | 23.43 |
| Not Affordable | 81 | 5.66 |
| Don’t Know | 12 | 0.84 |
| Decline To Answer | 2 | 0.14 |
| Missing | 12 | 0.84 |
| **The ease to obtain male condoms** |  |  |
| Very Easy | 996 | 69.65 |
| Somewhat Easy | 309 | 21.61 |
| Not Easy | 105 | 7.34 |
| Don’t Know | 6 | 0.42 |
| Decline To Answer | 2 | 0.14 |
| Missing | 12 | 0.84 |
| **Where FSW usually get male condoms** |  |  |
| Government hospital/clinic/health center | 846 | 846/1430 |
| Family planning clinic | 216 | 216/1430 |
| Mobile clinic or outreach | 207 | 207/1430 |
| HIV counseling and testing site (VCT site) | 259 | 259/1430 |
| Private hospital or clinic | 49 | 49/1430 |
| Shop/super market | 73 | 73/1430 |
| Pharmacy/chemist/drug store | 772 | 772/1430 |
| Peer educator or NGO | 303 | 303/1430 |
| Neighbourhood market/standard kiosk | 39 | 39/1430 |
| Friend | 659 | 659/1430 |
| Sex partner | 92 | 92/1430 |
| Shebeen or bar | 23 | 23/1430 |
| Service station | 54 | 54/1430 |
| Others |  |  |
| **In the last 6 months when you had sexual intercourse, did you or your partner ever put the condom on after you already started having sex** |  |  |
| Yes | 343 | 23.99 |
| No | 1069 | 74.76 |
| Don’t Know | 2 | 0.14 |
| Decline To Answer | 4 | 0.28 |
| Missing | 12 | 0.84 |
| **In the last 6 months when you had sexual intercourse, did you or your partner ever take the condom off before you were finished having sex** |  |  |
| Yes | 166 | 11.61 |
| No | 1242 | 86.85 |
| Don’t Know | 2 | 0.14 |
| Decline To Answer | 8 | 0.56 |
| Missing | 12 | 0.84 |
| **In the last 6 months when you had sexual intercourse, did the condom ever break/** |  |  |
| Yes | 177 | 12.38 |
| No | 1230 | 86.01 |
| Don’t Know | 5 | 0.35 |
| Decline To Answer | 6 | 0.42 |
| Missing | 12 | 0.84 |
| **In the last 6 months when you had sexual intercourse, did the condom ever slip o** |  |  |
| Yes | 147 | 10.28 |
| No | 1263 | 88.32 |
| Don’t Know | 1 | 0.07 |
| Decline To Answer | 7 | 0.49 |
| Missing | 12 | 0.84 |
| **Ever heard of a female condom** |  |  |
| Yes | 951 | 66.50 |
| No | 463 | 32.38 |
| Don’t Know | 1 | 0.07 |
| Decline To Answer | 3 | 0.21 |
| Missing | 12 | 0.84 |
| **Ever used a female condom** |  |  |
| **Yes** | 162 | 11.33 |
| No | 793 | 55.45 |
| **How often do you use female condoms** |  |  |
| Always | 11 | 6.79 |
| Usually | 23 | 14.20 |
| Sometimes | 83 | 51.23 |
| Rarely | 44 | 27.16 |
| Don't Know | 1 | 0.62 |
| **Where they get female condoms** |  |  |
| Government hospital/clinic/health center | 71 | 71/162 |
| Family planning clinic | 12 | 12/162 |
| Mobile clinic or outreach | 14 | 14/162 |
| HIV counseling and testing site (VCT site) | 25 | 25/162 |
| Private hospital or clinic | 4 | 4/162 |
| Shop/super market | 2 | 2/162 |
| Pharmacy/chemist/drug store | 68 | 68/162 |
| Peer educator or NGO | 34 | 34/162 |
| Neighbourhood market/standard kiosk | 1 | 1/162 |
| Friend | 45 | 45/162 |
| Others | 14 | 14/162 |

**HIV knowledge, opinion, attitude, and prevention of HIV/AIDS**

Table 14 shows estimates of knowledg**e**, opinion, attitude, and prevention of HIV/AIDS amongst FSWs in Sierra Leone. Approximately 70.0% of the FSWs provided correct responses to questions on knowledg**e**, opinion, attitude, and prevention of HIV/AIDS (Table 4). Approximately 36% of the FSWs indicated that a person can get HIV by sharing food with someone infected.

**Table 14: HIV knowledge, opinion, attitude, and prevention of HIV/AIDS**

|  | Freq | Percent |
| --- | --- | --- |
| **Can people reduce their chance of getting HIV by having just one uninfected sex** |  |  |
| Correct response | 1144 | 80.00 |
| Wrong response | 260 | 18.18 |
| Don't Know | 26 | 1.82 |
| **Can people get HIV from mosquito bites?** |  |  |
| Wrong response | 313 | 21.89 |
| Correct response | 1015 | 70.98 |
| Don't Know | 102 | 7.13 |
| **Can people reduce their chance of getting HIV by using a condom every time they have sex** |  |  |
| Correct response | 1157 | 80.91 |
| Wrong response | 256 | 17.90 |
| Don't Know | 17 | 1.19 |
| **Can a person get HIV by sharing food with someone who is infected?** |  |  |
| Wrong response | 512 | 35.80 |
| Correct response | 832 | 58.18 |
| Don't Know | 85 | 5.94 |
| No Response | 1 | 0.07 |
| **Can people get HIV because of supernatural means (juju, witchcraft, black magic)** |  |  |
| Wrong response | 169 | 11.82 |
| Correct response | 1144 | 80.00 |
| Don't Know | 117 | 8.18 |
| **Is it possible for a healthy-looking person to have HIV?** |  |  |
| Correct response | 1144 | 80.00 |
| Wrong response | 246 | 17.20 |
| Don't Know | 39 | 2.73 |
| No Response | 1 | 0.07 |
| **Can a person get HIV by getting injections with a needle that was already used by someone else** |  |  |
| Correct responses | 1274 | 89.09 |
| Wrong responses | 136 | 9.51 |
| Don't Know | 20 | 1.40 |
| **Can an infected mother transmit HIV to her unborn child during pregnancy?** |  |  |
| Correct response | 932 | 65.17 |
| Wrong response | 326 | 22.80 |
| Don't Know | 172 | 12.03 |
| **Can a woman with HIV transmit the virus to her newborn child during delivery?** |  |  |
| Correct response | 931 | 65.10 |
| Wrong response | 326 | 22.80 |
| Don't Know | 172 | 12.03 |
| No Response | 1 | 0.07 |
| **Can a woman with HIV transmit the virus to her newborn child through breastfeeding** |  |  |
| Correct response | 963 | 67.34 |
| Wrong response | 300 | 20.98 |
| Don't Know | 166 | 11.61 |
| No Response | 1 | 0.07 |
| **Are there any special drugs that a doctor or a nurse can give to a woman infected with HIV to reduce risk of transmission to the baby** |  |  |
| Correct response | 938 | 65.59 |
| Wrong response | 349 | 24.41 |
| Don't Know | 142 | 9.93 |
| No Response | 1 | 0.07 |
| **Have you heard about special antiretroviral drugs (e.g. ARV, nevirapine, zidovudine)** |  |  |
| Yes | 958 | 66.99 |
| No | 413 | 28.88 |
| Don't Know | 57 | 3.99 |
| No Response | 2 | 0.14 |
| **Do you know anyone who has died from AIDS?** |  |  |
| Yes | 371 | 25.94 |
| No | 1059 | 74.06 |
| **Who died from HIV** |  |  |
| Relative in my immediate family | 31 | 31/371 |
| Relative in my extended family | 35 | 35/371 |
| Neighbour | 123 | 123/371 |
| Colleague | 27 | 27/371 |
| Sexual partner | 10 | 10/371 |
| Other | 11 | 11/371 |
| **Did you help to care for him/her before be he/she died** |  |  |
| Yes | 134 | 36.12 |
| No | 236 | 63.88 |

**Coverage of prevention programs**

Approximately 69.4% (n=993) FSWs are aware of civil society or NGOs that deliver non-medical assistance or advice to FSW and more than half (n=809, 56.6%) of the FSWs attended meetings to discuss HIV/AIDS in the last 12 months.

**Table 15: Exposure to interventions**

| **Exposure to interventions** | Freq | Percent |
| --- | --- | --- |
| **Are you aware of any civil society or any organization(s) that deliver non-medical assistance or advice to FSW?** |  |  |
| Yes | 993 | 69.44 |
| No | 411 | 28.74 |
| Don't Know | 16 | 1.12 |
| No Response | 10 | 0.70 |
| **During the last 12 months, have you attended any meetings to discuss HIV and/or AIDs** |  |  |
| Yes | 809 | 56.57 |
| No | 609 | 42.59 |
| Don't Know | 4 | 0.28 |
| No Response | 8 | 0.56 |
| **In the last 18 months, have you been in contact with a health peer educator in the community** |  |  |
| Yes | 906 | 63.36 |
| No | 501 | 35.03 |
| Don't Know | 16 | 1.12 |
| No Response | 7 | 0.49 |
| **In the last 12 months, how many times have you been in contact with the peer educator** |  |  |
| Less than 3 times | 544 | 58.56 |
| 3-5 times | 304 | 32.72 |
| More than 5 times | 81 | 8.72 |
| **Services received from a peer educator** |  |  |
| General HIV/STI prevention/transmission information | 801 | 801/929 |
| Condoms & Lubricant | 556 | 556/929 |
| Referral for STI treatment | 174 | 174/929 |
| Drop in centres (DICs) | 47 | 47/929 |
| Legal aid in collaboration with NAS and legal Aid Board | 6 | 6/929 |
| Recreation space and safe havens | 3 | 3/929 |
| ART | 3 | 3/929 |
| Other | 12 | 12/929 |
| **Did the peer educator refer you for medical care at a health center?** |  |  |
| Yes | 629 | 67.71 |
| No | 281 | 30.25 |
| Don't Know | 9 | 0.97 |
| No Response | 10 | 1.08 |
| **Did you go to the referred site to receive medical care?** | Freq. | Percent |
| Yes | 524 | 80.86 |
| No | 107 | 16.51 |
| Don't Know | 7 | 1.08 |
| No Response | 10 | 1.54 |

**Stigma, discrimination, and violence**

Table 16 shows estimates for experiences of discrimination stigma and violence due to FSW status, as perceived and experienced by FSWs in the six regional headquarter towns in Sierra Leone. For experiences of stigma, discrimination, and violence, the FSWs were asked to report on any experiences of discrimination in the following sectors: healthcare, employment, education, church/religion, social settings such as restaurants, bar services, housing services and services provided by the police. About 30% of the FSWs reported that they had verbal insults directed at them because someone believed they sell sex to men and 8.4% reported sexual assault in the past 12 months. The majority of FSWs (over 90% of the sample) across the six regional headquarter towns reported to have not experienced any stigma, discrimination, and violence based on their FSW status in any of the abovementioned indicators.

**Table 16: Stigma, discrimination, and violence**

| **Stigma, discrimination, and violence** | Freq | Percent |
| --- | --- | --- |
| **In the past 12 months, have you been refused health care because someone believe you sell sex to men** |  |  |
| Yes | 29 | 2.03 |
| No | 1394 | 97.48 |
| Don't Know | 5 | 0.35 |
| Decline to Answer | 2 | 0.14 |
| **In the past 12 months, have you been refused employment because someone believed you sell sex to men** |  |  |
| Yes | 42 | 2.94 |
| No | 1385 | 96.85 |
| Don't Know | 2 | 0.14 |
| Decline to Answer | 1 | 0.07 |
| **In the past 12 months, have you been refused church/religious service because someone believed you sell sex to men** |  |  |
| Yes | 10 | 0.70 |
| No | 1418 | 99.16 |
| Don't Know | 2 | 0.14 |
| **In the past 12 months, have you been refused restaurant/bar service because someone believed you sell sex to men** |  |  |
| Yes | 8 | 0.56 |
| No | 1419 | 99.23 |
| Don't Know | 2 | 0.14 |
| Decline to Answer | 1 | 0.07 |
| **In the past 12 months, have you been refused housing because someone believed you sell sex to men** |  |  |
| Yes | 15 | 1.05 |
| No | 1415 | 98.95 |
| **In the past 12 months, have you been refused police assistance because someone believed you sell sex to men** |  |  |
| Yes | 92 | 6.43 |
| No | 1336 | 93.43 |
| Don't Know | 1 | 0.07 |
| Decline to Answer | 1 | 0.07 |
| **In the past 12 months, have you had verbal insults directed at you because someone believed you sell sex to men** |  |  |
| Yes | 429 | 30.00 |
| No | 999 | 69.86 |
| Decline to Answer | 2 | 0.14 |
| **Who was the person who last directed a verbal insult at you?** |  |  |
| Do not know the Person | 18 | 4.18 |
| Social Acquaintance | 100 | 23.20 |
| Family/Relative | 115 | 26.68 |
| Police | 8 | 1.86 |
| Client | 90 | 20.88 |
| Other Sex Worker | 50 | 11.60 |
| Non-Paying Partner Or Boyfriend | 8 | 1.86 |
| Other (Specify) | 39 | 9.05 |
| Don't Know | 2 | 0.46 |
| Decline to Answer | 1 | 0.23 |
| **In the past 12 months, have you been hit, kicked, or beaten because someone believed you sell sex to men** |  |  |
| Yes | 243 | 16.99 |
| No | 1182 | 82.66 |
| Don't Know | 1 | 0.07 |
| Decline to Answer | 4 | 0.28 |
| **Who was the person who last hit, kicked, or beat you?** |  |  |
| Do Not Know the Person | 14 | 5.65 |
| Social Acquaintance | 16 | 6.45 |
| Family/Relative | 40 | 16.13 |
| Police | 12 | 4.84 |
| Client | 121 | 48.79 |
| Other Sex Worker | 4 | 1.61 |
| Pimp | 5 | 2.02 |
| Non-Paying Partner Or Boyfriend | 22 | 8.87 |
| Other (Specify) | 10 | 4.03 |
| Don’t Know | 1 | 0.40 |
| Decline to Answer | 3 | 1.21 |
| **In the past 12 months, did anyone force you to have sex with them by sexually assaulting or raping you** |  |  |
| Yes | 120 | 8.39 |
| No | 1304 | 91.19 |
| Decline to Answer | 6 | 0.42 |
| **Who was the person who last forced you to have sex with them?** |  |  |
| Do Not Know The Person | 16 | 12.70 |
| Social Acquaintance | 22 | 17.46 |
| Family/Relative | 4 | 3.17 |
| Police | 6 | 4.76 |
| Client | 49 | 38.89 |
| Other Sex Worker | 1 | 0.79 |
| Pimp | 11 | 8.73 |
| Non-Paying Partner Or Boyfriend | 10 | 7.94 |
| Other (Specify) | 2 | 1.59 |
| Don’t Know | 1 | 0.79 |
| Decline to Answer | 4 | 3.17 |
| **Did you seek medical treatment after this happened** |  |  |
| Yes | 52 | 41.27 |
| No | 71 | 56.35 |
| Decline to Answer | 3 | 2.38 |
| **Did you report this incident to the police?** |  |  |
| Yes | 33 | 26.19 |
| No | 91 | 72.22 |
| Decline to Answer | 2 | 1.59 |

**Alcohol and drug use**

Table 17 presents data about alcohol and drug use among FSWs in Sierra Leone. Approximately 46% of the FSW had never taken alcohol in the last three months ( n=651) and 40% had not taken marijuana in the last six months.

**Table 17: Alcohol and drug use among FSW**

| **Alcohol and drug use** | Freq | Percent |
| --- | --- | --- |
| **During the past 3 months, how often have you had a drink containing alcohol?** |  |  |
| Never | 651 | 45.52 |
| Monthly Or less | 254 | 17.76 |
| 2-4 Times A Month | 171 | 11.96 |
| 2-3 Times A Week | 181 | 12.66 |
| 4 Or More Times A Week | 119 | 8.32 |
| Don'T Know | 35 | 2.45 |
| Decline To Answer | 19 | 1.33 |
| **During the past 3 months, how many drinks containing alcohol did you have on a typical day when you are drinking** |  |  |
| 1 Or 2 | 312 | 21.82 |
| 3 Or 4 | 284 | 19.86 |
| 5 Or 6 | 116 | 8.11 |
| 7 Or 9 | 28 | 1.96 |
| 10 Or More | 21 | 1.47 |
| Don't Know | 217 | 15.17 |
| Decline to Answer | 452 | 31.61 |
| **During the past 3 months, how often did you have six or more drinks on one occasion** |  |  |
| Never | 595 | 41.61 |
| Monthly Or Less | 184 | 12.87 |
| 2-4 Times A Month | 141 | 9.86 |
| 2-3 Times A Week | 110 | 7.69 |
| 4 Or More Times A Week | 74 | 5.17 |
| Don't Know | 112 | 7.83 |
| Decline to Answer | 214 | 14.97 |
| **In the past 6 months, have you taken Heroin?** |  |  |
| Did Not Use This Drug In The Last 6 Months | 783 | 54.76 |
| Monthly Or less | 8 | 0.56 |
| Several Times A Month | 18 | 1.26 |
| Two To Four Times A Month | 5 | 0.35 |
| Two To Three Times A Week | 5 | 0.35 |
| Four Or More Times A Week | 4 | 0.28 |
| Don’t know | 287 | 20.07 |
| Decline to answer | 320 | 22.38 |
| **In the past 6 months, have you taken cocaine** |  |  |
| Did not use this drug in the last 6 months | 797 | 55.73 |
| Monthly Or Less | 11 | 0.77 |
| Several Times A Month | 4 | 0.28 |
| Two To Four Times A Month | 5 | 0.35 |
| Two To Three Times A Week | 2 | 0.14 |
| Four Or More Times A Week | 1 | 0.07 |
| Don’t Know | 269 | 18.81 |
| Decline to Answer | 341 | 23.85 |
| **In the past 6 months, have you taken Crack** |  |  |
| Did Not Use This Drug In The Last 6 Months | 792 | 55.38 |
| Monthly Or Less | 9 | 0.63 |
| Several Times A Month | 31 | 2.17 |
| Two To Four Times A Month | 4 | 0.28 |
| Two To Three Times A Week | 3 | 0.21 |
| Four Or More Times A Week | 6 | 0.42 |
| Don't Know | 252 | 17.62 |
| Decline to Answer | 333 | 23.29 |
| **In the past 6 months, have you taken Methamphetamine** |  |  |
| Did Not Use This Drug In The Last 6 Months | 802 | 56.08 |
| Monthly Or Less | 1 | 0.07 |
| Several Times A Month | 1 | 0.07 |
| Four Or More Times A Week | 1 | 0.07 |
| Don't Know | 317 | 22.17 |
| Decline to Answer | 308 | 21.54 |
| **In the past 6 months, have you taken Speedball** |  |  |
| Did Not Use This Drug In The Last 6 Months | 805 | 56.29 |
| Monthly Or Less | 1 | 0.07 |
| Several Times A Month | 1 | 0.07 |
| Two To Four Times A Month | 1 | 0.07 |
| Four Or More Times A Week | 3 | 0.21 |
| Don’t Know | 319 | 22.31 |
| Decline to Answer | 300 | 20.98 |
| **In the past 6 months, have you taken Tramadol drugs** |  |  |
| Did Not Use This Drug In The Last 6 Months | 661 | 46.22 |
| Monthly Or Less | 65 | 4.55 |
| Several Times A Month | 58 | 4.06 |
| Two To Four Times A Month | 39 | 2.73 |
| Two To Three Times A Week | 45 | 3.15 |
| Four Or More Times A Week | 21 | 1.47 |
| Don’t Know | 221 | 15.45 |
| Decline to Answer | 320 | 22.38 |
| **In the past 6 months, have you taken solvent/glue** |  |  |
| Did Not Use This Drug In The Last 6 Months | 804 | 56.22 |
| Monthly Or Less | 2 | 0.14 |
| Several Times A Month | 3 | 0.21 |
| Two To Four Times A Month | 5 | 0.35 |
| Two To Three Times A Week | 1 | 0.07 |
| Don't Know | 291 | 20.35 |
| Decline to Answer | 324 | 22.66 |
| **In the past 6 months, have you taken Hallucinogens** |  |  |
| Did Not Use This Drug In The Last 6 Months | 804 | 56.22 |
| Monthly Or Less | 2 | 0.14 |
| Two To Three Times A Week | 1 | 0.07 |
| Four Or More Times A Week | 3 | 0.21 |
| Don't Know | 297 | 20.77 |
| Decline to Answer | 323 | 22.59 |
| **In the past 6 months, have you taken Marijuana** |  |  |
| Did Not Use This Drug In The Last 6 Months | 570 | 39.86 |
| Monthly Or Less | 36 | 2.52 |
| Several Times A Month | 104 | 7.27 |
| Two To Four Times A Month | 64 | 4.48 |
| Two To Three Times A Week | 95 | 6.64 |
| Four Or More Times A Week | 115 | 8.04 |
| Don't Know | 163 | 11.40 |
| Decline to Answer | 283 | 19.79 |
| **In the past 6 months, have you taken Pethidine drugs?** |  |  |
| Did Not Use This Drug In The Last 6 Months | 801 | 56.01 |
| Monthly Or Less | 3 | 0.21 |
| Several Times A Month | 1 | 0.07 |
| Two To Three Times A Week | 5 | 0.35 |
| Don't Know | 296 | 20.70 |
| Decline to Answer | 324 | 22.66 |
| **In the past 6 months, have you taken Pentazoncine drugs?** |  |  |
| Did Not Use This Drug In The Last 6 Months | 803 | 56.15 |
| Don't Know | 302 | 21.12 |
| Decline to Answer | 325 | 22.73 |
| **In the past 6 months, have you taken Chinese capsules drugs?** |  |  |
| Did Not Use This Drug In The Last 6 Months | 798 | 55.80 |
| Monthly Or Less | 3 | 0.21 |
| Several Times A Month | 9 | 0.63 |
| Two To Four Times A Month | 3 | 0.21 |
| Four Or More Times A Week | 3 | 0.21 |
| Don't Know | 303 | 21.19 |
| Decline to Answer | 311 | 21.75 |
| **In the past 6 months, have you taken Blue boat(Valium 10 mg) drugs?** |  |  |
| Did Not Use This Drug In The Last 6 Months | 802 | 56.08 |
| Monthly Or Less | 2 | 0.14 |
| Several Times A Month | 3 | 0.21 |
| Two To Four Times A Month | 4 | 0.28 |
| Two To Three Times A Week | 4 | 0.28 |
| Four Or More Times A Week | 4 | 0.28 |
| Don't Know | 287 | 20.07 |
| Decline to Answer | 324 | 22.66 |
| **In the past 6 months, have you taken Diazepan drugs?** |  |  |
| Did Not Use This Drug In The Last 6 Months | 800 | 55.94 |
| Monthly Or Less | 6 | 0.42 |
| Several Times A Month | 5 | 0.35 |
| Two To Four Times A Month | 3 | 0.21 |
| Two To Three Times A Week | 4 | 0.28 |
| Four Or More Times A Week | 1 | 0.07 |
| Don’t Know | 287 | 20.07 |
| Decline to Answer | 324 | 22.66 |
| **Some people have tried injecting drugs using a syringe or needle. In the past 6 month, have you injected drugs?** |  |  |
| Yes | 60 | 4.20 |
| No | 1242 | 86.85 |
| Don’t Know | 19 | 1.33 |
| Decline to Answer | 109 | 7.62 |
| **In the last 3 months, how frequently did you inject drugs?** |  |  |
| Monthly Or Less | 28 | 14.89 |
| Two To Four Times A Month | 13 | 6.91 |
| Two To Three Times A Week | 10 | 5.32 |
| Four Or More Times A Week | 6 | 3.19 |
| Don’t know | 19 | 10.11 |
| Decline to answer | 112 | 59.57 |

**Information on STIs**

Table 18 shows the information on previous HIV testing and perception of risk. The majority of FSWs sampled across the six regional headquarter towns reported to have ever tested for HIV (n=1012, 70.8%). A small percentage of the FSWs who have had HIV tests indicated that they tested positive for HIV (n=39, 3.9%). Among the 418 FSWs that had never had an HIV test, 177 indicated that they don’t just don’t have the time to test for HIV.

**Table 18: Previous HIV testing and perception of risk**

| **Previous HIV testing and perception of risk** | Freq | Percent |
| --- | --- | --- |
| **Have you ever been tested for HIV?** |  |  |
| Yes | 1012 | 70.77 |
| No | 418 | 29.23 |
| **If no, why** |  |  |
| Don't know where to go | 79 | 79/418 |
| Always use condoms | 74 | 74/418 |
| Not at risk of getting HIV | 54 | 54/418 |
| Didn't have time/too busy | 177 | 177/418 |
| Trust my partner | 11 | 11/418 |
| Afraid of knowing I may be HIV-Positive | 71 | 71/418 |
| Lack of confidentiality | 44 | 44/418 |
| Inconvenient testing location or hours | 6 | 6/418 |
| **Where was the last test done?** |  |  |
| Government Hospital/Clinic/Health Center | 466 | 46.05 |
| Private Facility Clinic | 37 | 3.66 |
| Mobile Counselling And Testing Outreach | 229 | 22.63 |
| School Or University | 1 | 0.10 |
| Work/Employer | 2 | 0.20 |
| An Ngo (Specify) | 148 | 14.62 |
| Other Specify | 125 | 12.35 |
| Don’t Know | 4 | 0.40 |
| **Reason for doing the test** |  |  |
| Wanted to know my HIV status | 924 | 924/1012 |
| My partner asked me to get tested | 11 | 11/1012 |
| Wanted to start sexual relations with a new partner | 5 | 5/1012 |
| Wanted to get married | 5 | 5/1012 |
| Need for loan/insurance coverage | 5 | 5/1012 |
| I felt sick | 72 | 72/1012 |
| Advised by a health worker | 80 | 80/1012 |
| Advised by a peer educator | 20 | 20/1012 |
| Other | 22 | 22/1012 |
| **What was the result of your last HIV test?** |  |  |
| HIV-Negative | 932 | 92.09 |
| HIV-Positive | 39 | 3.85 |
| Indeterminate | 14 | 1.38 |
| I didn’t get the results | 7 | 0.69 |
| Don’t know | 18 | 1.78 |
| No response | 2 | 0.20 |
| **What do you think your chances of transmitting HIV to a partner is?** |  |  |
| No Chance | 6 | 15.38 |
| Small Chance | 4 | 10.26 |
| Moderate Chance | 1 | 2.56 |
| Great Chance | 22 | 56.41 |
| Don't Know | 4 | 10.26 |
| No Response | 2 | 5.13 |
| **What do you think are your chances of getting HIV?** |  |  |
| No Chance | 818 | 58.81 |
| Small Chance | 161 | 11.57 |
| Moderate Chance | 121 | 8.70 |
| Great Chance | 138 | 9.92 |
| Don't Know | 145 | 10.42 |
| No Response | 8 | 0.58 |
| **Why do they think they have chances of getting HIV?** |  |  |
| Fidelity to partner/trust in partner | 137 | 137/818 |
| Use condoms | 870 | 870/818 |
| **Why they think that they have a moderate chance/great chance of getting HIV?** |  |  |
| Don't use condoms | 139 | 139/259 |
| Don't trust partner | 79 | 79/259 |
| Had injuries/cuts | 13 | 13/259 |
| Multiple partners | 161 | 161/259 |
| Injecting drugs | 3 | 3/259 |
| **What do you think your status is today?** |  |  |
| HIV-Negative | 1058 | 76.06 |
| HIV-Positive | 12 | 0.86 |
| Don’t Know | 314 | 22.57 |
| No Response | 7 | 0.50 |
| **How satisfied were you with the quality of services provided at the place where you got your last test** |  |  |
| Very Satisfied | 728 | 71.94 |
| Satisfied | 252 | 24.90 |
| A Little Satisfied | 5 | 0.49 |
| Not Satisfied | 2 | 0.20 |
| Don’t know | 22 | 2.17 |
| No Response | 3 | 0.30 |
| **At any time during your most recent counseling and testing experience, did you reveal to the counsellor or Healthcare provider that you exchange sex for money** |  |  |
| Yes | 609 | 60.18 |
| No | 368 | 36.36 |
| Don’t know | 26 | 2.57 |
| No response | 9 | 0.89 |
| **Why did you do that** |  |  |
| Afraid provider would tell police/legal authority | 44 | 44/368 |
| Did not feel it was necessary to discuss | 160 | 160/368 |
| Afraid provider would not keep my information confidential | 88 | 88/368 |
| Little or no contact/interaction with counselor or provider | 46 | 46/368 |
| Shy/embarrassed | 71 | 71/368 |
| Provider already knew | 35 | 35/368 |
| **Did you feel that a counselor or health care provider reacted to you in a negatively** |  |  |
| Yes | 24 | 3.33 |
| No | 663 | 92.08 |
| Don’t know | 24 | 3.33 |
| No response | 9 | 1.25 |

Prevalence and sociodemographic determinants of HIV among FSWs in Sierra Leone

This section presents the estimated prevalence of HIV and the corresponding 95% confidence interval estimate among FSW in the six regional headquarter towns in Sierra Leone.

The results from the logit-transformed confidence intervals with venue-based cluster robust standard error showed that the overall prevalence of HIV among FSWs in the six regional headquarter towns was estimated to be 11.8% (95% CI: 7.9-17.1). Table 8 shows the adjusted HIV prevalence estimates and sociodemographic determinants of HIV among FSW in Sierra Leone. The HIV prevalence estimates differed by average monthly income, marital status, geographic location of the FSW, level of education of the FSW. HIV prevalence

Estimates were high in each regional headquarter town, highest in Freetown with an estimated prevalence of 20.9% (95% CI:17.60-24.6) and lowest in Waterloo and Grafton with an estimated prevalence of 4.3% (95% CI:2.6-7.2). The HIV prevalence was higher among FSWs with primary education (17.2% 95% CI: 8.5-31.7) compared to those with tertiary or higher education (7.1%, 95% CI:0.9-39.3). The FSWs who were never married or co-habiting had a lower prevalence of HIV (9.9%, 95% CI:6.5-14.7) compared to those who were in some form of relationship (married, divorced, cohabiting, etc) (17.1% 95% CI:13.6-21.3). FSWs that earned more than 500000 Leone (50 USD equivalent) had a higher prevalence of HIV (16.6%, 95% CI: 10.9-24.4) compared to those that earned less than 500000 Leone (10.2%; 95% CI: 6.8-15.1). The prevalence of HIV among FSWs that live/worked in Western urban areas was approximately 4 times (unadjusted prevalence ratio; uPR=3.8, 95% CI: 2.2-6.9, p<0.05; Table 8) the prevalence of HIV among those that live/work in Bombali. The prevalence of HIV among FSWs who have ever or currently been in a relationship was approximately 2 times the prevalence of HIV among FSWs who have never been married or cohabiting ( uPR=1.7, 95% CI:1.4-2.2, p<0.05; p<0.05; Table 8). The prevalence among FSWs who earn on average 50 USD or higher a month had an increased prevalence of HIV compared to those who earn less than 50USD (uPR=1.62; 95% CI: 1.2-2.2; p<0.05; Table 19).

Table 19: HIV prevalence among female sex workers by sociodemographic characteristics and related risk factors

|  | HIV status | Unadjusted prevalence ratio from Modified Poisson Regression Model | Unadjusted Odds ratio from the binary logistic regression model |
| --- | --- | --- | --- |
| **Sociodemographic** | HIV+ (%) [95% CI] | uPR [95% CI] | uOR [95% CI] |
| **Age in years** |  |  |  |
| 18-24 years | 10.47 [6.60-16.21] | 1 | 1 |
| 25-29 years | 12.89 [8.82-18.48 | 1.23 [0.91-1.66] | 1.27 [0.90-1.77] |
| 30-39 years | 14.62 [8.90-23.09] | 1.40 [0.98-1.99] | 1.47 [0.97-2.21] |
| 40+ years | 4.35 [1.00-16.98] | 0.42 [0.09-1.84] | 0.39 [0.08-1.86] |
| **Highest level of education** |  |  |  |
| None | 11.25 [7.56-16.43] | 1 | 1 |
| Primary | 17.16 [8.47-31.67] | 1.52 [1.03-2.26]* | 1.63 [1.00-2.64]* |
| JSS | 11.73 [8.15-16.59] | 1.04 [0.88-1.23] | 1.05 [0.87-1.26] |
| SSS/ Technical Vocational | 9.49 [6.96-12.81] | 0.84 [0.64-1.10] | 0.83 [0.61-1.12] |
| Higher | 7.14 [0.91-39.31] | 0.63 [0.09-4.29] | 0.61 [0.07-5.41] |
| **Current employment status** |  |  |  |
| Employed | 13.34 [10.91-16.21] | 1 | 1 |
| Unemployed | 10.60 [8.63-12.96] | 0.79 [0.46-1.36] | 0.77 [0.42-1.42] |
| **Household income categorized** |  |  |  |
| < 50 USD | 10.22 [6.78-15.12] | 1 | 1 |
| 50 USD or more | 16.56 [10.86-24.42] | 1.62 [1.17-2.24]** | 1.74 [1.19-2.54]** |
| **Marital status at the moment** |  |  |  |
| Never married or cohabited | 9.90 [6.54-14.70] | 1 | 1 |
| Others | 17.07 [13.57-21.26] | 1.73 [1.36-2.19]*** | 1.87 [1.42-2.48]*** |
| **Religion** |  |  |  |
| Christian | 11.31 [6.89-18.02] | 1 | 1 |
| Moslem | 11.99 [8.37-16.88] | 1.06 [0.81-1.38] | 1.07 [0.79-1.44] |
| **Regions** |  |  |  |
| Northern | 8.26 [5.35-12.55] | 1 | 1 |
| Eastern | 8.33 [5.08-13.39] | 1.01 [0.56-1.83] | 1.01 [0.53-1.93] |
| Southern | 11.76 [5.51-23.37] | 1.42 [0.63-3.21] | 1.48 [0.60-3.68] |
| Western | 16.00 [10.13-24.35] | 1.94 [1.07-3.49]*** | 2.12 [1.08-4.15]*** |
| **District** |  |  |  |
| Bombali | 5.38 [2.99-9.50] | 1 | 1 |
| Port Loko | 11.11 [5.90-19.95] | 2.06 [0.93-4.60] | 2.20 [0.91-5.28] |
| Kenema | 8.33 [5.08-13.39] | 1.55 [0.75-3.21] | 1.60 [0.73-3.48] |
| Bo | 11.76 [5.51-23.37] | 2.19 [0.89-5.35] | 2.34 [0.87-6.30]* |
| Western Urban | 20.88 [17.60-24.58] | 3.88 [2.17-6.92]*** | 4.46 [2.49-8.64]*** |
| Western Rural | 4.32 [2.57-7.17] | 0.80 [00.38-1.69] | 0.79 [0.36-1.74] |
| **Town/City name** |  |  |  |
| Makeni | 5.38 [2.99-9.50] | 1 | 1 |
| Lingui | 11.11 [5.90-19.95] | 2.06[0.93-4.60] | 2.20[10.91-5.28] |
| Kenema | 8.33 [5.08-13.39] | 1.55 [0.75-3.21] | 1.60 [0.73-3.48] |
| Bo | 11.76 [5.51-23.37] | 2.19 [0.89-5.35] | 2.34 [0.87-6.30] |
| Freetown | 20.88 [17.60-24.58] | 3.88 [2.17-6.92]*** | 4.64 [2.49-8.64]*** |
| Waterloo and Grafton | 4.32 [2.57-7.17] | 0.80 [0.38-1.69] | 0.79 [0.36-1.74] |
| **Ethnic affiliation** |  |  |  |
| Mende | 11.52 [7.06-18.22] | 1 | 1 |
| Temne | 10.15 [6.70-15.11] | 0.88 [0.53-1.46] | 0.87 [0.49-1.53] |
| Limba | 10.95 [5.42-20.88] | 0.95 [0.50-1.82] | 0.94 [0.46-1.96] |
| Others (Mandingo, Kono, Krio, Kuranko, etc) | 14.49 [8.74-23.08] | 1.26 [0.87-1.82] | 1.30 [0.85-1.99] |
| **Type of FSW** |  |  |  |
| Roamer | 12.35 [8.12-18.34] | 1 | 1 |
| Seater | 10.93 [6.48-17.84] | 0.88 [0.56-1.41] | 0.87 [0.51-1.47] |

Abbrevaiation: FSW: Female Sex Workers, uPR: Unadjusted Prevalence Ratio, our: Unadjusted odds ratio; CI: Confidence interval. P-value notation: ***p<0.001, **p<0.01, *p<0.05.

Prevalence of HIV by sexual history and risk behavior among FSW in Sierra Leone

Table 9 presents the prevalence of HIV by sexual history and risk behaviour among FSW in Sierra Leone. HIV prevalence was higher (13.5%, 95% CI: 9.7-18.5) among FSWs who first received the money exchanged for sex at the age of 18 years or higher compared to those less than 18 years (9.2%, 95% CI: 4.9-16.6, Table 9). The FSWs that exchanged sex for money after 18 years had a higher prevalence of HIV (uPR=1.5; 95% CI: 1.0-2.2; p<0.05; Table 20).

Table 20: Prevalence of HIV by sexual history and risk behaviour among FSW in Sierra Leone

| **Sexual history and risk behaviour of FSW in Sierra Leone** | HIV status | Unadjusted prevalence ratio from Modified Poisson Regression Model | Unadjusted Odds ratio from binary logistic regression model |
| --- | --- | --- | --- |
|  | HIV+ (%) [95% CI] | uPR [95% CI] | uOR [95% CI] |
| **Age in years at first vaginal sex** |  |  |  |
| <18 years | 11.52 [7.21-17.91] | 1 | 1 |
| 18 or older | 11.86 [8.26-16.74] | 1.03 [0.71-1.50] | 1.03 [0.67-1.58] |
| **Age first receive money for sex** |  |  |  |
| Below 18 | 9.24 [4.94-16.61] | 1 | 1 |
| 18+ years | 13.52 [9.73-18.47] | 1.46 [0.99-2.16]* | 1.54 [1.01-22.34]* |
| **Number of non-paying men FSW had vaginal or anal sex with in the last 3 months** |  |  |  |
| All paid (Zero) | 12.36 [8.58-17.48] | 1 | 1 |
| Only one did not pay | 11.38 [6.13-20.16] | 0.92 [0.63-1.34] | 0.91 [0.60-1.39] |
| Two or more did not pay | 10.42 [7.17-14.91] | 0.84 [0.67-1.06] | 0.82 [0.63-1.07] |
| **Number of client paying partners in the last 3 months** |  |  |  |
| 1-5 | 12.09 [7.20-19.60] | 1 | 1 |
| 6-10 | 12.75 [8.21-19.28] | 1.05 [0.64-1.75] | 1.06 [0.60-1.89] |
| 11-19 | 6.92 [3.44-13.42] | 0.57 [0.36-0.92]* | 0.54 [0.32-0.90]* |
| 20 or more | 12.12 [7.65-18.68] | 1.00 [0.68-1.48] | 1.00 [0.64-1.57] |
| **Condom use last time FSW had sex** |  |  |  |
| Yes | 11.72 [7.28-18.33] | 1 | 1 |
| No | 12.18 [8.66-16.88] | 1.04 [0.72-1.49] | 1.05 [0.69-1.57] |
| **Use of lubricants** |  |  |  |
| Always | 17.61 [10.50-28.02] | 1 | 1 |
| Usually | 12.28 [7.59-19.26] | 0.70 [0.43-1.13] | 0.66 [0.37-1.15] |
| Sometimes | 11.59 [7.03-18.52] | 0.66 [0.38-1.14] | 0.61 [0.32-1.17] |
| Never | 10.84 [6.83-16.79] | 0.62 [0.34-1.12] | 0.57 [0.28-1.14] |
| **Ever tested for HIV** |  |  |  |
| Yes | 11.76 [7.53-17.90] | 1 | 1 |
| No | 11.72 [8.21-16.47] | 1.00 [0.74-1.34] | 1.00 [0.71-1.39] |

Abbrevaiation: FSW: Female Sex Workers, uPR: Unadjusted Prevalence Ratio, our: Unadjusted odds ratio; CI: Confidence interval. P-value notation: ***p<0.001, **p<0.01, *p<0.05.

Prevalence of HIV by knowledge, opinions, and attitudes among FSW in Sierra Leone

Table 10 presents the prevalence of HIV by knowledge, opinions, and attitudes among FSW in Sierra Leone. HIV prevalence was higher (15.0%, 95% CI: 9.9-22.3) among FSWs who provided wrong responses to the question “Can people reduce their chance of getting HIV by having just one uninfected sex partner who has no other sex partners?” compared to those who provided correct responses (10.9%, 95% CI: 7.4-15.8, table 11). The FSWs that provided correct responses had a lower prevalence of HIV (uPR=0.7; 95% CI: 0.6-1.0; p<0.05; Table 21).

Table 21: Prevalence of HIV by knowledge, opinions, and attitudes among FSW in Sierra Leone

| Knowledge, opinions, and attitudes | HIV status | Unadjusted prevalence ratio from Modified Poisson Regression Model | Unadjusted Odds ratio from the binary logistic regression model |
| --- | --- | --- | --- |
|  | HIV+ (%) [95% CI] | uPR [95% CI] | uOR [95% CI] |
| **Can people reduce their chance of getting HIV by having just one uninfected sex partner who has no other sex partners?** |  |  |  |
| Wrong response | 15.03 [9.85-22.27] | 1 | 1 |
| Correct response | 10.93 [7.40-15.84] | 0.73 [0.56-0.94]* | 0.69 [0.51-0.94]* |
| **Can people get HIV from mosquito bites?** |  |  |  |
| Wrong response | 14.46 [9.16-22.06] | 1 | 1 |
| Correct response | 10.64 [7.32-15.21] | 0.74 [0.57-0.95]* | 0.70 [0.52-0.95]* |
| **Can people reduce their chance of getting HIV by using a condom every time they have sex?** |  |  |  |
| Wrong response | 12.09 [6.51-21.34] | 1 | 1 |
| Correct response | 11.67 [8.16-16.42] | 0.97 [0.69-1.36] | 0.96 [0.65-1.42] |
| **Can a person get HIV by sharing food with someone who is infected?** |  |  |  |
| Wrong response | 11.67 [8.12-16.50] | 1 | 1 |
| Correct response | 12.11 [6.59-21.19] | 1.04 [0.75-1.44] | 1.04 [0.72-1.52] |
| **Can people get HIV because of supernatural means (juju, witchcraft, black magic etc)?** |  |  |  |
| Wrong response | 12.94 [7.31-21.88] | 1 | 1 |
| Correct response | 11.45 [7.87-16.37] | 0.89 [0.61-1.28] | 0.87 [0.57-1.33] |
| **Is it possible for a healthy-looking person to have HIV?** |  |  |  |
| Wrong response | 10.84 [6.67-17.13] | 1 | 1 |
| Correct response | 11.98 [7.81-17.92] | 1.10 [0.70-1.74] | 1.12 [0.67-1.86] |
| **Can a person get HIV by getting injections with a needle that was already used by someone else?** |  |  |  |
| Wrong response | 10.26 [6.81-15.17] | 1 | 1 |
| Correct response | 11.93 [7.74-17.94] | 1.16 [0.70-1.93] | 1.19 [0.67-2.10] |
| **Can an infected mother transmit HIV to her unborn child during pregnancy?** |  |  |  |
| Wrong response | 12.05 [7.72-18.31] | 1 | 1 |
| Correct response | 11.59 [7.84-16.81] | 0.96 [0.77-1.20] | 0.96 [0.74-1.23] |
| **Can a woman with HIV transmit the virus to her newborn child during delivery?** |  |  |  |
| Wrong response | 11.22 [7.08-17.33] | 1 | 1 |
| Correct response | 12.03 [8.10-17.50] | 1.07 [0.84-1.37] | 1.08 [0.82-1.43] |
| **Can a woman with HIV transmit the virus to her newborn child through breastfeeding?** |  |  |  |
| Wrong response | 11.78 [7.48-18.06] | 1 | 1 |
| Correct response | 11.73 [7.96-16.97] | 1.00 [0.80-1.24] | 1.00 [0.78-1.28] |
| **Are there any special drugs that a doctor or a nurse can give to a woman infected with HIV to reduce the risk of transmission to the baby?** |  |  |  |
| Wrong response | 11.79 [7.88-17.28] | 1 | 1 |
| Correct response | 11.73 [7.49-17.89] | 0.99 [0.71-1.40] | 0.99 [0.67-1.47] |
| **Have you heard about special antiretroviral drugs (e.g. ARV, nevirapine, zidovudine, lamivudine) that people infected with HIV can get from a doctor or a nurse to help them live longer?** |  |  |  |
| No | 11.65 [7.70-17.26] | 1 | 1 |
| Yes | 11.80 [7.65-17.75] | 1.01 [0.74-1.39] | 1.01 [0.71-1.45] |
| **Overall knowledge, opinion and attitudes towards HIV/AIDS** |  |  |  |
| Low (0-6/13) | 13.75 [7.64-23.50] | 1 | 1 |
| Moderate (7-10/13) | 11.67 [7.81-17.09] | 0.85 [0.52-1.39] | 0.83 [0.47-1.47] |
| High (11-13/13) | 11.21 [7.15-17.14] | 0.81 [0.52-1.28] | 0.79 [0.47-1.33] |

Abbrevaiation: FSW: Female Sex Workers, uPR: Unadjusted Prevalence Ratio, our: Unadjusted odds ratio; CI: Confidence interval. P-value notation: ***p<0.001, **p<0.01, *p<0.05.

Prevalence of HIV among FSWs by stigma, violence and risk behaviour in Sierra Leone

HIV prevalence was higher (16.7%, 95% CI: 9.7-27.1) among FSWs who inject drugs compared to non-injecting drug users (10.6%, 95% CI: 7.3-15.2). The prevalence of HIV increased by approximately 2 times among those who inject drugs compared to non-drug-injecting users (uPR=1.6, 95% CI: 1.1-2.2, p<0.05; Table 11). However, experiencing rape in the last 12 months, alcohol intake in the past 3 months, and general drug use were not found to be associated with HIV (Table 22).

Table 22: Prevalence of HIV among FSWs by stigma, violence and risk behaviour in Sierra Leone

| Stigma, discrimination, violence, and risk behaviour | HIV status | Unadjusted prevalence ratio from Modified Poisson Regression Model | Unadjusted Odds ratio from the binary logistic regression model |
| --- | --- | --- | --- |
|  | HIV+ (%) [95% CI] | uPR [95% CI] | uOR [95% CI] |
| **Rape in the past 12 months** |  |  |  |
| No | 11.66 [7.83-17.00] | 1 | 1 |
| Yes | 13.33 [8.05-21.29] | 1.14 [0.80-1.64] | 1.17 [0.77-1.76] |
| **Alcohol intake in the past 3 months** |  |  |  |
| No alcohol | 13.67 [8.64-20.97] | 1 | 1 |
| Alcohol | 10.39 [6.65-15.89] | 0.76 [0.48-1.21] | 0.73 [0.43-1.25] |
| **Drug use in the past 3 months** |  |  |  |
| No drug | 12.03 [8.57-16.64] | 1 | 1 |
| Drug | 11.26 [6.64-18.47] | 0.94 [0.72-1.22] | 0.93 [0.69-1.25] |
| **Inject drugs in the past 3 months** |  |  |  |
| No | 10.63 [7.29-15.24] | 1 | 1 |
| Yes | 16.67 [9.70-27.13] | 1.57 [1.10-2.23]* | 1.68 [1.10-2.57]* |

Abbrevaiation: FSW: Female Sex Workers, uPR: Unadjusted Prevalence Ratio, uOR: Unadjusted odds ratio; CI: Confidence interval. P-value notation: ***p<0.001, **p<0.01, *p<0.05.

Prevalence of HIV among FSWs by exposure to the intervention

The prevalence of HIV did not vary by the exposure to the various intervention. That is, attending meeting (s) to discuss HIV and /or AIDS in the last 12 months, being aware of any civil society or organizations that deliver non-medical assistance or advice to FSW, and having contact with health peer navigator in the last 12 months on HIV and AIDS-related services did not have a statistically significant effect on HIV prevalence (Table 23).

Table 23: Prevalence of HIV among FSWs by exposure to the intervention

| Exposure to intervention | HIV status | Unadjusted prevalence ratio from Modified Poisson Regression Model | Unadjusted Odds ratio from the binary logistic regression model |
| --- | --- | --- | --- |
|  | HIV+ (%) [95% CI] | uPR [95% CI] | uOR [95% CI] |
| **Aware of any civil society or organizations that deliver non-medical assistance or advice to FSW** |  |  |  |
| Yes | 12.39 [8.12-18.44] | 1 | 1 |
| No | 10.46 [6.87-15.61] | 0.84 [0.62-1.15] | 0.83 [0.58-1.17] |
| **Attended meeting (s) to discuss HIV and /or AIDS in the last 12 months** |  |  |  |
| Yes | 12.11 [7.94-18.06] | 1 | 1 |
| No | 11.33 [7.69-16.39] | 0.94 [0.76-1.15] | 0.93 [0.73-1.17] |
| **Contact with health peer navigator in the last 12 months on HIV and AIDS-related services** |  |  |  |
| Yes | 11.70 [7.45-17.90] | 1 | 1 |
| No | 12.18 [7.87-18.36] | 1.04 [0.70-1.54] | 1.05 [0.67-1.63] |

Abbrevaiation: FSW: Female Sex Workers, uPR: Unadjusted Prevalence Ratio, uOR: Unadjusted odds ratio; CI: Confidence interval. P-value notation: ***p<0.001, **p<0.01, *p<0.05.

Factors associated with HIV among FSW in Sierra Leone: a multivariable regression analysis

The results from the multivariable modified Poisson regression model showed that marital status, the district where the FSW live and operate, monthly income, HIV and AIDs related knowledge, and injecting drugs are associated with higher HIV prevalence among FSW in Sierra Leone. The prevalence of HIV among FSWs that live/worked in Western urban area was approximately 4 times (adjusted prevalence ratio; aPR=3.6, 95% CI: 1.7-7.5, p<0.05; Table 13) the prevalence of HIV among those that live/work in Bombali. The prevalence of HIV among FSWs who have ever or currently been in a relationship was approximately 2 times the prevalence of HIV among FSWs who have never been married or cohabiting ( aPR=1.8, 95% CI:1.4-2.3, p<0.05; Table 8). The prevalence among FSWs who earn on average 50 USD or higher a month was twice the prevalence of HIV among FSWs who earn less than 50USD (aPR=1.8; 95% CI: 1.2-2.7; p<0.05; Table 13). The HIV prevalence was lower among FSWs that provided correct responses to the following questions: Can people reduce their chance of getting HIV by having just one uninfected sex partner who has no other sex partners and whether people can get HIV from mosquito bites (Table 24).

Table 24: Factors associated with HIV among FSW in Sierra Leone: a multivariable regression analysis

| Factors | Adjusted prevalence ratio from Modified Poisson Regression Model | Adjusted Odds ratio from the binary logistic regression model |
| --- | --- | --- |
|  | aPR [95% CI] | aOR [95% CI] |
| **Highest level of education** |  |  |
| None | 1 |  |
| Primary | 1.51[0.95-2.39] | 1.70 [0.95-3.05]* |
| JSS | 0.85 [0.51-1.43] | 0.84[0.44-1.59] |
| SSS/ Technical Vocational | 0.72 [0.39-1.35] | 0.68[0.32-1.45] |
| Higher | 1.01 [0.12-8.37] | 0.99[0.08-12.60] |
| **Marital status** |  |  |
| Never married/cohabited | 1 |  |
| Others | 1.76 [1.36-2.28]*** | 2.05[1.50-2.78]*** |
| **District** |  |  |
| Bombali | 1 | 1 |
| Port Loko | 2.36 [0.55-10.12] | 2.54 [0.49-13.25] |
| Kenema | 1.93 [0.84-4.42] | 1.98 [0.80-4.88] |
| Bo | 2.45 [0.81-7.48] | 2.63 [0.73-9.46] |
| Western Urban | 3.57 [1.70-7.53]*** | 4.27 [1.90-9.58]*** |
| Western Rural | 0.10 [0.38-2.62] | 0.96 [0.34-2.72] |
| **Household income categorized** |  |  |
| < 50 USD | 1 | 1 |
| 50 USD or more | 1.83 [1.24-2.70]*** | 2.12 [1.28-3.51]*** |
| **Age first receive money for sex** |  |  |
| Below 18 | 1 | 1 |
| 18+ years | 1.23 [0.79-1.92] | 1.30 [0.77-2.18] |
| **Can people reduce their chance of getting HIV by having just one uninfected sex partner who has no other sex partners?** |  |  |
| Wrong response | 1 | 1 |
| Correct response | 0.64 [0.50-0.82]*** | 0.57 [0.41-0.79]*** |
| **Can people get HIV from mosquito bites?** |  |  |
| Wrong response | 1 | 1 |
| Correct response | 0.70 [0.51-0.96]** | 0.63 [0.43-0.93]** |
| **Number of client paying partners in the last 3 months** |  |  |
| 1-5 | 1 | 1 |
| 6-10 | 0.88 [0.47-1.62] | 0.84 [0.39-1.83] |
| 11-19 | 0.62 [0.35-1.10] | 0.54 [0.29-1.03] |
| 20 or more | 0.85 [0.56-1.30] | 0.80 [0.46-1.38] |
| **Inject drugs in the past 3 months** |  |  |
| No | 1 | 1 |
| Yes | 1.66 [0.98-2.83] | 2.12 [1.01-4.46]** |

Abbrevaiation: FSW: Female Sex Workers, aPR: adjusted Prevalence Ratio, aOR: adjusted odds ratio; CI: Confidence interval. P-value notation: ***p<0.001, **p<0.01, *p<0.05.

**Sexual history of MSM**

Table 25 shows the sexual history of MSMs in Sierra Leone covered by the study. The majority (62.4%; n=350) of the MSM have ever had a vaginal sex and relative minority made up of 41.0% (n=230) have also ever had anal sex. Of the MSMs who have had vaginal sex, majority (72.9%; n=369) reported having had sex in the last three months of the study with only one woman (41.5%) or two or more women (31.4%). Also, of the MSMs who had vaginal sex with one or more women, relative majority made up of 48.0 % (n=129) did not use any condom at all; the rest reported using condom on only one occasion (25.8%: n=139) or two or more occasions (15.3%; n=82). Of the MSMs who reported having had anal sex with a man, the majority (64.7%; n=558) first had anal sex with a man at 18 years or above. A high majority (83.5%: n=361) also reported having sexual intercourse with up to two men. Again, majority of the MSMs (58.9%; n=316) reported not using condoms. Two out of three (67.8%; n=374) reported having been tested for HIV.

**Table 25: Sexual history of MSM**

| **Sexual history of MSM** | **Frequency** | **Percent** |
| --- | --- | --- |
| **Ever had a vaginal sex (N=561)** |  |  |
| Yes | 350 | 62.39 |
| No | 211 | 37.61 |
| **Ever had anal sex (N=561)** |  |  |
| Yes | 230 | 41.00 |
| No | 331 | 59.00 |
| **Number of women you had vaginal sex with in the last three month (N=369)** |  |  |
| None | 100 | 27.10 |
| Only 1 | 153 | 41.46 |
| 2 or more | 116 | 31.44 |
| **Of these, how many did you not use condom (N=269)** |  |  |
| None | 129 | 47.96 |
| Only 1 | 103 | 38.29 |
| 2 or more | 37 | 13.75 |
| **Age at first anal sex with a man (N=558)** |  |  |
| 3-17 years | 197 | 35.30 |
| 18+ | 361 | 64.70 |
| **Number of men you have sexual intercourse with (N=558)** |  |  |
| 1-2 | 466 | 83.51 |
| 3 or more | 92 | 16.49 |
| **Of these, how many did you not use condom with (N=537)** |  |  |
| None | 316 | 58.85 |
| Only one | 139 | 25.88 |
| 2 or more | 82 | 15.27 |
| **Number of people you’ve had vaginal or anal sex in the last three months (N=561)** |  |  |
| Less than 2 | 276 | 49.20 |
| 2 or more | 285 | 50.80 |
| **Ever been tested for HIV (N=560)** |  |  |
| No | 186 | 33.21 |
| Yes | 374 | 66.79 |

**Sexual and behavioural risk factors and violence of MSM**

The Integrated Behavioural Surveillance Survey (IBBSS) data collected in Sierra Leone asked men who have sex with men a number of questions regarding their sexual and violence behaviour. These questions are used as a proxy for their sexual and behavioural risk to being infected with sexually transmitted diseases (STDs). The results are presented in Table 3.

Almost 8 in 10 (78.0%) men who have sex with men have never put the condom on after they have already started having sex in the past 6 months during sexual intercourse. A vast majority of MSMs (91.8%) have never experience condom break or leak during sexual intercourse in the past 6 months. Among the MSMs interviewed, 506 (92.2%) reported that they have never taken off the condom during sexual intercourse before they were finished having sex. Of the 536 interviewed, a little over one-third (36.9%) reported of using lubricant always during vaginal or anal sex and 48.5% reported that they have never used lubricant during vaginal or anal sex. Alcohol use and injection of drugs was reported by 206 men (37.1%) and 48 men (9.4%) respectively. Rape was reported by 3.9% (n=22) of MSM.

**Table 26: Sexual and behavioural risk factors and violence of MSM**

| **Sexual and behavioural risk factors and violence** | **Frequency** | **Percent** |
| --- | --- | --- |
| **In the last 6 months when you had sexual intercourse, did you or your partner ever put the condom on after you already started having sex (N=550)** |  |  |
| Yes | 121 | 22.00 |
| No | 429 | 78.00 |
| **In the last 6 months when you had sexual intercourse, did the condom ever break/leak during sex or while pulling out? (N=548)** |  |  |
| Yes | 45 | 8.21 |
| No | 503 | 91.79 |
| **In the last 6 months when you had sexual intercourse, did you or your partner ever take the condom off before you were finished having sex? (N=549)** |  |  |
| Yes | 43 | 7.83 |
| No | 506 | 92.17 |
| **In the last 6 months when you had sexual intercourse, did the condom ever slip off during sex or while pulling out? (N=548)** |  |  |
| Yes | 37 | 6.75 |
| No | 511 | 93.25 |
| **How often do you use lubricant during vaginal or anal sex? (N=536)** |  |  |
| Always | 198 | 36.94 |
| Usually | 78 | 14.55 |
| Rarely/never | 260 | 48.51 |
| **Alcohol use (N=555)** |  |  |
| Never drank alcohol | 349 | 62.88 |
| Use alcohol | 206 | 37.12 |
| **Inject drugs (N=511)** |  |  |
| Yes | 48 | 9.39 |
| No | 463 | 90.61 |
| **Rape (N=560)** |  |  |
| Yes | 22 | 3.93 |
| No | 538 | 96.07 |

**HIV-related knowledge among MSM**

HIV weakens the immune system and makes the body prone to other opportunistic diseases. A significant number of all new HIV infections occur among key populations including men who have sex with men (MSM). To address these problems, HIV interventions and results oriented programmes are needed to enable effective HIV service delivery. Men who have sex with men were asked series of questions regarding knowledge of HIV prevention methods, misconceptions about HIV/AIDs transmission and knowledge about mother-to-child transmission (MTCT).

Table 27 shows that approximately half (49. 8%) of MSM interviewed had moderate knowledge about HIV. Regarding knowledge of HIV prevention methods, 81.8% of MSM know that limiting sexual intercourse to one uninfected partner can reduce the chances of getting HIV and 84.5% of MSM know that the consistent use of condom prevents the chance of contracting HIV.

In terms of misconceptions about HIV transmission, 87.3% of MSM know that HIV is not contracted by supernatural means; 69.7% of MSM know that HIV is not contracted from mosquito bites and 69.2% know that HIV cannot be contracted by sharing food with an infected person. Concerning knowledge about mother-to-child transmission (MTCT), 65.4% of MSM are aware that HIV can be transmitted through breastfeeding; 61.7% know that the risk of mother-to-child transmission can be reduced by taking special medication and 67.9% of MSM know that antiretroviral drugs (ARV) prolong the lifespan of people living with HIV (PLHIV).

**Table 27: HIV-related knowledge among MSM**

| **HIV-related knowledge among MSM** | **Frequency** | **Percent** |
| --- | --- | --- |
| **Can people reduce their chance of getting HIV by having just one uninfected sex partner who has no other sex partners? (N=561)** |  |  |
| Wrong | 102 | 18.18 |
| Correct | 459 | 81.82 |
| **Can people get HIV from mosquito bites? (N=561)** |  |  |
| Wrong | 170 | 30.30 |
| Correct | 391 | 69.70 |
| **Can people reduce their chance of getting HIV by using a condom every time they have sex? (N=561)** |  |  |
| Wrong | 87 | 15.51 |
| Correct | 474 | 84.49 |
| **Can a person get HIV by sharing food with someone who is infected? (N=561)** |  |  |
| Wrong | 173 | 30.84 |
| Correct | 388 | 69.16 |
| **Can people get HIV because of supernatural means (juju, witchcraft, black magic etc)? (N=561)** |  |  |
| Wrong | 71 | 12.66 |
| Correct | 490 | 87.34 |
| **Is it possible for a healthy-looking person to have HIV? (N=561)** |  |  |
| Wrong | 109 | 19.43 |
| Correct | 452 | 80.57 |
| **Can a person get HIV by getting injections with a needle that was already used by someone else? (N=561)** |  |  |
| Wrong | 43 | 7.66 |
| Correct | 518 | 92.34 |
| **Can an infected mother transmit HIV to her unborn child during pregnancy? (N=561)** |  |  |
| Wrong | 208 | 37.08 |
| Correct | 353 | 62.92 |
| **Can a woman with HIV transmit the virus to her newborn child during delivery? (N=561)** |  |  |
| Wrong | 194 | 34.58 |
| Correct | 367 | 65.42 |
| **Can a woman with HIV transmit the virus to her newborn child through breastfeeding? (N=561)** |  |  |
| Wrong | **194** | **34.58** |
| Correct | **367** | **65.42** |
| **Are there any special drugs that a doctor or a nurse can give to a woman infected with HIV to reduce the risk of transmission to the baby? (N=561)** |  |  |
| Wrong | 215 | 38.32 |
| Correct | 346 | 61.68 |
| **Have you heard about special antiretroviral drugs (e.g. ARV, nevirapine, zidovudine, lamivudine) that people infected with HIV can get from a doctor or a nurse to help them live longer?** |  |  |
| Wrong | 180 | 32.09 |
| Correct | 381 | 67.91 |
| **Overall knowledge scores** |  |  |
| >=6 Low knowledge | 61 | 10.85 |
| 7-9 Moderate | 280 | 49.82 |
| 10-12 High knowledge | 221 | 39.32 |

**MSM exposure to interventions**

Civil society organizations (CSOs) are key community actors in the national effort to curb HIV and AIDS as public health epidemic. These CSOs provide of critical HIV related services like general HIV testing services (HTS), distribution of condom and lubricants, community delivery of ART (CDA), social and psychosocial support to people living with HIV (PLHIV) and other key populations (KPs). These activities ensure the continued mobilization of community members for service delivery and the promotion of the social and human rights within the society. In assessing the activities of CSO with the community among MSM, series of question were asked to all MSM who participated in the survey.

Table 5 shows the percentage distribution of MSM exposure to intervention. In all, 59.4% of MSM are aware of any civil society or any organization(s) that deliver non-medical assistance or advice to MSM. Majority of the MSM (61.6%, n=343) reported that in the last 6 months preceding the survey, they have been in contact with a health peer navigator in the community. However, relatively fewer MSM (53.8%, n=301) have attended any meeting to discuss HIV and AIDS in the last 6 months preceding the survey.

**Table 28: MSM exposure to interventions**

| **Exposure to HIV interventions** | **Frequency** | **Percent** |
| --- | --- | --- |
| **Are you aware of any civil society or any organization(s) that deliver non-medical assistance or advice to MSM? (N=557)** |  |  |
| Yes | 331 | 59.43 |
| No | 226 | 40.57 |
| **In the last 6 months, have you been in contact with a health peer navigator in the community? (N=557)** |  |  |
| Yes | 343 | 61.58 |
| No | 214 | 38.42 |
| **During the last 6 months, have you attended any meetings to discuss HIV and/or AIDS? (N=560)** |  |  |
| Yes | 301 | 53.75 |
| No | 259 | 46.25 |

**HIV services for HIV positive patients**

Access and utilization of HIV related services by people living with HIV (PLHIV) including differentiated service delivery (DSD), multi month dispensing (MMD) and other community delivery of ART (CDA) that aid in reduction in the rate of interruption in treatment, and retention on treatment. This do not only promote quality healthy life among PLHIV but also help in the attainment of the 3^rd^ 90-90-90 global goal which aim at attaining 90% of all people reeiving antiretroviral therapy have viral suppression.

Table 29 presents the percentage distribution of MSM living with HIV and access to HIV services. Among MSM living with HIV, 7 out of 10 representing 70% are on treatment with only 14.3% reported ever forgetting to take their ART. More than half (54.6%, n=6) of MSM know where people can get advice about HIV.

**Table 29: HIV services for HIV positive patients**

| **HIV services for HIV positive patients** | **Frequency** | **Percent** |
| --- | --- | --- |
| **On treatment (N=10)** |  |  |
| Yes | 7 | 70.00 |
| No | 3 | 30.00 |
| **Forget to take ART (N=7)** |  |  |
| Yes | 1 | 14.29 |
| No | 6 | 85.71 |
| **Know where people can get advice about HIV (N=11)** |  |  |
| Yes | 6 | 54.55 |
| No | **5** | 45.45 |

**HIV prevalence among MSM by sociodemographic/economic factors in the six regional headquarter towns in Sierra Leone**

Table 30 shows crude and adjusted estimates overall HIV prevalence amongst MSM in the six regional headquarter towns in Sierra Leone. Overall, the crude and adjusted HIV prevalence estimates among MSM was 3.2% [95% CI: 2.0-5.3%] and 3.4% [95% CI: 1.9-5.8] respectively. The prevalence was highest among those MSM in the Western Province (9.2%; 95% CI: 5.1-16.2) compared to those in the North (0.1%, 95% CI: 0.01-0.6). The prevalence of HIV among those that live in the Western province was approximately 13 times (95% CI: 1.7-94.5) as high as the prevalence of HIV among those that live in the Northern province. The HIV prevalence was higher (6.9%; 95% CI: 3.0-14.8) among high income earners (>500000 Leone per month: 50 USD equivalent) compared to those that earn less than 50 USD per month (1.5%; 95% CI: 0.58-3.81). Muslims who are MSM had a lower prevalence of HIV (1.4%; 95% CI: 0.48-3.81) compared to Christians (7.5%; 95%: 3.9-14.0).

**Table 30: HIV prevalence among MSM by sociodemographic characteristics**

|  | Crude/unadjusted estimate of HIV prevalence | RDS design-based weighted estimated | Unadjusted prevalence ratio from double selection Lasso Poisson Regression Model adjusting for sampling weight from RDS | Unadjusted Odds ratio from Firth Penalized maximum likelihood logistic regression model adjusting for sampling weight from RDS |
| --- | --- | --- | --- | --- |
| Sociodemographic characteristics | HIV+ (%) [95% CI] | HIV+ (%) [95% CI] | uPR [95% CI] | uOR [95% CI] |
| **Age in years** |  |  |  |  |
| 17-24 years | 1.15 [0.43-3.03] | 1.35 [0.42-4.26] | 1 | 1 |
| 25-29 years | 6.25 [3.15-12.02] | 5.94 [2.66-12.73] | 5.44 [1.66-17.77]** | 5.36 [1.68-17.10]** |
| 30+ | 7.06 [3.20-14.86] | 7.63 [2.77-19.35] | 6.14 [1.77-21.30]** | 6.23 [1.83-21.20]** |
| **Education level** |  |  |  |  |
| None/primary | 2.04 [0.29-13.16] | 0.45 [0.06-3.22] | 1 | 1 |
| JHS | 5.88 [2.22-14.68] | 6.89 [2.26-19.13] | 2.88 [0.33-25.05] | 2.24 [0.34-14.72] |
| SHS/Tech | 2.56 [1.38-4.69] | 2.51 [1.15-5.37] | 1.25 [0.16-9.60] | 0.89 [0.16-5.05] |
| Higher | 5.66 [1.83-16.17] | 7.74 [2.25-23.48] | 2.77 [0.30-25.83] | 2.23 [0.32-15.69] |
| **Income** |  |  |  |  |
| <500000 | 1.72 [0.82-3.57] | 1.50 [0.58-3.81] | 1 | 1 |
| 500000+ | 6.35 [3.20-12.20] | 6.86 [3.03-14.76] | 3.69 [1.36-9.99]** | 3.80 [1.40-10.36]** |
| **Employment status** |  |  |  |  |
| Employed | 5.52 [2.99-9.97] | 5.53 [2.63-11.23] | 1 | 1 |
| Unemployed | 2.11 [1.06-4.17] | 2.32 [0.99-5.36] | 0.38 [0.15-0.95]* | 0.38 [0.15-0.94]* |
| **Marital status** |  |  |  |  |
| Married/divorced etc | 7.22 [3.47-14.39] | 7.57 [2.91-18.31] | 1 | 1 |
| Single | 2.37 [1.32-4.23] | 2.54 [1.28-5.00] | 0.33 [0.13-0.83]* | 0.30 [0.12-0.78]* |
| **Religion** |  |  |  |  |
| Christian | 6.63 [3.89-11.10] | 7.54 [3.91-14.04] | 1 | 1 |
| Moslem | 1.37 [0.57-3.26] | 1.37 [0.48-3.81] | 0.21 [0.07-0.57]** | 0.20 [0.07-0.56]** |
| **Ethnicity** |  |  |  |  |
| Mende | 1.94 [0.62-5.84] | 0.93 [0.27-3.17] | 1 | 1 |
| Temne | 1.20 [0.30-4.70] | 1.52 [0.23-9.21] | 0.62 [0.11-3.68] | 0.67 [0.13-3.47] |
| Others | 5.42 [3.17-9.11] | 6.30 [3.38-11.46] | 2.80 [0.81-9.67] | 2.59 [0.79-8.58] |
| **District** |  |  |  |  |
| Bombali District | 1.12 [0.16-7.57] | 0.14 [0.02-1.04] | 1 | 1 |
| Port Loko | No observation | No observation | No observation | No observation |
| Kenema | 1.19 [0.17-8.00] | 0.80 [0.11-5.55] | 1.06 [0.07-16.68] | 1.07 [0.11-10.50] |
| Kono | No observation | No observation | No observation | 0.43 [0.02-10.73] |
| Bo | 1.33 [0.19-8.89] | 1.40 [0.20-9.38] | 1.19 [0.08-18.67] | 1.19 [0.12-11.73] |
| Western Area Urban | 11.36 [6.96-18.01] | 11.98 [6.63-20.68] | 10.11 [1.36-75.20]* | 7.77 [1.41-42.62]* |
| Wester Area Rural | No observation | No observation | No observation | 0.58 [0.02-14.54] |
| **Province** |  |  |  |  |
| Northern | 0.65 [0.09-4.51] | 0.08 [0.01-0.60] | 1 | 1 |
| Eastern | 0.66 [0.09-4.54] | 0.47 [0.07-3.29] | 1.01 [0.06-15.99] | 0.99 [0.10-9.63] |
| Southern | 1.33 [0.19-8.89] | 1.40 [0.20-9.38] | 2.04 [0.13-32.25] | 2.00 [0.20-19.65] |
| Western | 8.24 [5.02-13.23] | 9.24 [5.10-16.17] | 12.61 [1.68-94.54]** | 9.26 [1.71-50.22]** |

Abbreviation: MSM: Men who have sex with men, uPR: Unadjusted Prevalence Ratio, our: Unadjusted odds ratio; CI: Confidence interval. P-value notation: ***p<0.001, **p<0.01, *p<0.05.

**HIV prevalence among MSM by sexual history**

Table 8 shows the prevalence of HIV among MSM by sexual history. The prevalence estimate was higher among MSM who had ever have vaginal sex (4.6%; 95% CI: 2.4-8.01) compared to those who had never had vaginal sex (1.6%; 95% CI: 0.4-6.6). The prevalence of HIV reduced by 79% (aPR=0.21; 95% CI: 0.05-0.89) among MSM who had never had vaginal sex compared those who have had that experience.

**Table 31: HIV prevalence among MSM by sexual history**

|  | Crude/unadjusted estimate of HIV prevalence | **RDS design-based weighted estimated** | **Unadjusted prevalence ratio from double selection Lasso Poisson Regression Model adjusting for sampling weight from RDS** | **Unadjusted Odds ratio from Firth Penalized maximum likelihood logistic regression model adjusting for sampling weight from RDS** |
| --- | --- | --- | --- | --- |
| **Sexual history** | **HIV+ (%) [95% CI]** | **HIV+ (%) [95% CI]** | **uPR [95% CI]** | **uOR [95% CI]** |
| **Ever had a vaginal sex (N=561)** |  |  |  |  |
| Yes | 4.57[2.82- 7.34] | 4.46[2.44-8.01] | 1 | 1 |
| No | 0.95[0.24-3.72] | 1.59 [0.37-6.59] | 0.21[0.05 -0.89]* | 0.24[0.06- 0 .92]* |
| **Ever had anal sex (N=561)** |  |  |  |  |
| Yes | 2.17[0.91-5.12] | 2.13[0.74-5.93] | 1 | 1 |
| No | 3.93[2.29- 6.65] | 4.23[2.19-8.04] | 1.81 [0.65-5.00] | 1.74[0.64-4.75] |
| **Number of women you had vaginal sex with in the last three month (N=369)** |  |  |  |  |
| None | 7.00[3.37-13.99] | 4.31[1.83-9.80] | 1 | 1 |
| Only 1 | 3.92 [.77 - 8.47] | 6.47[2.77-14.36] | 0.56[0.19-1.62] | 0.55[0.19-1.62] |
| 2 or more | 2.59 [0.83 - 7.74] | 1.43[0.34-5.75] | 0.37[0.10-1.39] | 0.38[0.10-1.41] |
| **Age at first anal sex with a man (N=558)** |  |  |  |  |
| 3-17 years | 2.54[1.06-5.96] | 2.94[1.10-0.59] | 1 | 1 |
| 18+ | 3.60[2.10- 6.11] | 3.61[1.82-7.03] | 1.42 [0.51-3.93] | 1.36[0.50 -3.73] |
| **Number of men you have sexual intercourse with (N=558)** |  |  |  |  |
| 0-2 | 3.22[1.95-5.28] | 3.23[1.75-5.88] | 1 | 1 |
| 3 or more | 3.26[1.05- 9.65] | 4.13[1.03-15.07] | 1.01 [0.30-3.43] | 1.15[0.35- 3.74] |
| **Of these, how many did you not use condom with (N=537)** |  |  |  |  |
| None | 2.85[1.49-5.39] | 3.24[1.47-7.00] | 1 | 1 |
| Only one | 2.16[0.70- 6.49] | 1.55[0.42-5.62] | 0.76[0.21-2.76] | 0.83[0.24-2.87] |
| 2 or more | 2.44[0.61- 9.26] | 1.61[0.29-8.45] | 0.86[0.19-3.89] | 1.00 [0.24- 4.10] |
| **Number of people you’ve had vaginal or anal sex in the last three months (N=561)** |  |  |  |  |
| Less than 2 | 3.26[1.70 -6.16] | 2.67[1.21-5.78] | 1 | 1 |
| 2 or more | 3.16[1.65- 5.96] | 4.10[1.89-8.69] | 0.97[0.39-2.41] | 0.98[0.39- 2.45] |
| **Ever been tested for HIV (N=560)** |  |  |  |  |
| No | 2.15[0.81-5.60] | 3.06 [1.09 -8.31] | 1 | 1 |
| Yes | 3.74[2.23-6.23] | 3.53 [1.81-6.78] | 1.74[0.58 -5.22] | 1.64 [0.56- 4.78] |

Abbreviation: MSM: Men who have sex with men, uPR: Unadjusted Prevalence Ratio, our: Unadjusted odds ratio; CI: Confidence interval. P-value notation: ***p<0.001, **p<0.01, *p<0.05.

**HIV prevalence among MSM by sexual and behavioural risk factors**

Table 32 shows the prevalence of HIV among MSM and their sexual and behavioural risk factors. The HIV prevalence was higher (3.8%; 95% CI: 2.3-8.2) among MSM who drink alcohol compared to those who did not drink alcohol (3.2%; 95% CI: 1.5-6.4) although the difference was not statistically significant. The HIV prevalence was also higher among those who had been sexually abuse (4.8%; 95% CI: 0.1-5.4) compared to those who had never experience sexual violence (3.5%; 2.0-6.1) although the difference was not statistically significant. MSM who inject drugs had a higher HIV prevalence compared to non-injecting drug users.

**Table 32: HIV prevalence among MSM by sexual and behavioural risk factors**

|  | Crude/unadjusted estimate of HIV prevalence | RDS design-based weighted estimated | Unadjusted prevalence ratio from double selection Lasso Poisson Regression Model adjusting for sampling weight from RDS | Unadjusted Odds ratio from Firth Penalized maximum likelihood logistic regression model adjusting for sampling weight from RDS |
| --- | --- | --- | --- | --- |
| **Behavioural risk factors** | **HIV+ (%) [95% CI]** | **HIV+ (%) [95% CI]** | **uPR [95% CI]** | **uOR [95% CI]** |
| **In the last 6 months when you had sexual intercourse, did you or your partner ever put the condom on after you already started having sex (N=550)** |  |  |  |  |
| Yes | 1.65[0.41- 6.38] | 0.54[0.12-2.35] | 1 | 1 |
| No | 3.73[2.29- 6.01] | 4.27[2.40-7.49] | 2.26[0.53 -9.69] | 1.91[0.50 -7.34] |
| **In the last 6 months when you had sexual intercourse, did the condom ever break/leak during sex or while pulling out? (N=548)** |  |  |  |  |
| Yes | 2.22 [0.31- 14.21] | 2.06[0.28-13.45] | 1 | 1 |
| No | 3.38 [2.11-5.37] | 3.53 [1.98-6.22] | 1.52[0.21 -11.19] | 1.05 [0.19 -5.75] |
| **How often do you use lubricant during vaginal or anal sex? (N=536)** |  |  |  |  |
| Always | 3.54[1.69-7.24] | 3.18[1.27-7.69] | 1 | 1 |
| Usually | 3.85[1.24-11.28] | 4.27[1.04-15.98] | 1.09[0.29-4.11] | 1.19[0.33-4.36] |
| Rarely/never | 2.69[1.29-5.55] | 3.35[1.41-7.77] | 0.76[0.27-2.14] | 0.77[0.27 -2.14] |
| **Alcohol use (N=555)** |  |  |  |  |
| Never drank alcohol | 2.58[1.35- 4.89] | 3.16[1.53-6.42] | 1 | 1 |
| Use alcohol | 4.37[2.29- 8.19] | 3.79[1.57-8.87] | 1.69 [0.68-4.20] | 1.72[0.69-4.29] |
| **Inject drugs (N=511)** |  |  |  |  |
| Yes | 4.17 [1.04 -15.23] | 5.86[1.25 -23.46] | 1 | 1 |
| No | 2.59[1.48-4.51] | 2.38[1.18-4.77] | 0.62[0.14 -2.70] | 0.52[0.13- 2.07] |
| **Rape (N=560)** |  |  |  |  |
| Yes | 4.55[0.63-26.23] | 4.75[0.10-5.43] | 1 | 1 |
| No | 3.16[1.97- 5.03] | 3.49[1.99-6.08] | 0.70[0.10 -5.00] | 0.49[0.09-2.74] |

Abbreviation: MSM: Men who have sex with men, uPR: Unadjusted Prevalence Ratio, our: Unadjusted odds ratio; CI: Confidence interval. P-value notation: ***p<0.001, **p<0.01, *p<0.05.

**HIV-related knowledge among MSM**

Table 33 shows the HIV prevalence and HIV-related knowledge among MSM in the six regional headquarter towns. MSMs who provided correct responses to the following questions had a lower HIV prevalence compared to those who provided wrong responses: “Can people reduce their chance of getting HIV by having just one uninfected sex partner who has no other sex partners?”, “can people reduce their chance of getting HIV by using a condom every time they have sex?”, “can a person get HIV by getting injections with a needle that was already used by someone else?”, “ can a woman with HIV transmit the virus to her newborn child through breastfeeding?” etc.

**Table 33: HIV-related knowledge among MSM**

|  | Crude/unadjusted estimate of HIV prevalence | **RDS design-based weighted estimated** | **Unadjusted prevalence ratio from double selection Lasso Poisson Regression Model adjusting for sampling weight from RDS** | **Unadjusted Odds ratio from Firth Penalized maximum likelihood logistic regression model adjusting for sampling weight from RDS** |
| --- | --- | --- | --- | --- |
| **HIV-related knowledge among MSM** | **HIV+ (%) [95% CI]** | **HIV+ (%) [95% CI]** | **uPR [95% CI]** | **uOR [95% CI]** |
| **Can people reduce their chance of getting HIV by having just one uninfected sex partner who has no other sex partners? (N=561)** |  |  |  |  |
| Wrong | 4.90[2.05- 11.26] | 4.93[1.74-13.23] | 1 | 1 |
| Correct | 2.83[1.65- 4.82] | 2.99[1.54-5.73] | 0.58[0.21 - 1.59] | 0.54[0.19- 1.47] |
| **Can people get HIV from mosquito bites? (N=561)** |  |  |  |  |
| Wrong | 2.94[1.23-6.88] | 3.21[1.23-8.16 | 1 | 1 |
| Correct | 3.32[1.94- 5.65] | 3.44[1.73-6.73] | 1.13 [0.41 -3.12] | 1.10[0.40- 3.02] |
| **Can people reduce their chance of getting HIV by using a condom every time they have sex? (N=561)** |  |  |  |  |
| Wrong | 4.60[1.73- 11.64] | 7.36 [2.53 -19.56] | 1 | 1 |
| Correct | 2.95[1.75-4.93] | 2.65[1.39 -5.01] | 0.64 [0.22 - 1.91] | 0.58[0.20- 1.72] |
| **Can a person get HIV by sharing food with someone who is infected? (N=561)** |  |  |  |  |
| Wrong | 1.73[0.56 - 5.25] | 2.46[0.74-7.85] | 1 | 1 |
| Correct | 3.87[2.34-6.32] | 3.81[2.02-7.05] | 2.23[0.65 - 7.61] | 2.05[0.63- 6.63] |
| **Can people get HIV because of supernatural means (juju, witchcraft, black magic etc)? (N=561)** |  |  |  |  |
| Wrong | 4.23[1.37- 12.32] | 3.54[1.10-10.80] | 1 | 1 |
| Correct | 3.06[1.85- 5.02] | 3.33[1.79-6.14] | 0.72[0.21 -2.44] | 0.64[0.20-2.11] |
| **Is it possible for a healthy-looking person to have HIV? (N=561)** |  |  |  |  |
| Wrong | 0.92[0.13-6.25] | 0.52[ 0.07 -3.62] | 1 | 1 |
| Correct | 3.76[2.35- 5.97] | 4.06 [2.29 -7.09] | 4.10 [0.55 -30.53] | 2.92[0.54-15.65] |
| **Can a person get HIV by getting injections with a needle that was already used by someone else? (N=561)** |  |  |  |  |
| Wrong | 4.65 [1.16 - 16.83] | 6.29[1.26-26.11] | 1 | 1 |
| Correct | 3.09[1.90 -4.99] | 3.11[1.71-5.57] | 0.66[0.16-2.80] | 0.55[0.14- 2.16] |
| **Can an infected mother transmit HIV to her unborn child during pregnancy? (N=561)** |  |  |  |  |
| Wrong | 3.85[1.93-7.51] | 4.64[2.04-10.19] | 1 | 1 |
| Correct | 2.83[1.53-5.19] | 2.54[1.20-5.27] | 0.74 [0.30 - 1.84] | 0.73[0.289-1.83] |
| **Can a woman with HIV transmit the virus to her newborn child during delivery? (N=561)** |  |  |  |  |
| Wrong | 3.74[1.88-7.31] | 4.60[2.02-10.17] | 1 | 1 |
| Correct | 2.88[1.56-5.28] | 2.56[1.22-5.30] | 0.77[0.31 - 1.92] | 0.76[0.30-1.91] |
| **Can a woman with HIV transmit the virus to her newborn child through breastfeeding? (N=561)** |  |  |  |  |
| Wrong | 4.12[2.07-8.04] | 5.46[2.46-11.65] | 1 | 1 |
| Correct | 2.72[1.47-5.00] | 2.26[1.05- 4.81] | 0.66[0.26 - 1.65] | 0.64[0.26-1.62] |
| **Are there any special drugs that a doctor or a nurse can give to a woman infected with HIV to reduce the risk of transmission to the baby? (N=561)** |  |  |  |  |
| Wrong | 3.72[1.87-7.27] | 5.00[2.29-10.58] | 1 | 1 |
| Correct | 2.89[1.56-5.29] | 2.32[1.06-5.02] | 0.78[0.31-1.94] | 0.76[.30-1.92] |
| **Have you heard about special antiretroviral drugs (e.g. ARV, nevirapine, zidovudine, lamivudine) that people infected with HIV can get from a doctor or a nurse to help them live longer?** |  |  |  |  |
| Wrong | 1.11[0.28-4.34] | 1.28[0.32-5.00] | 1 | 1 |
| Correct | 4.20[2.59-6.75] | 4.33[2.36-7.81] | 3.78[0.88- 16.28] | 3.21[0.84-12.30] |
| **Overall knowledge scores** |  |  |  |  |
| >=6 Low knowledge | 3.28[0.82-12.22] | 6.78[1.72-23.21] | 1 | 1 |
| 7-9 Moderate | 3.21[1.68-6.07] | 3.17[1.52 -6.48] | 0.98[0.22- 4.43] | 0.84[0.20-3.47] |
| 10-12 High knowledge | 3.17[1.51- 6.50] | 2.51[0.89-6.82] | 0.97[0.21-4.54] | 0.85[0.10-3.66] |

Abbreviation: MSM: Men who have sex with men, uPR: Unadjusted Prevalence Ratio, our: Unadjusted odds ratio; CI: Confidence interval. P-value notation: ***p<0.001, **p<0.01, *p<0.05.

**Multivariable analysis of factors associated with HIV among MSM**

Table 34 shows the multivariable analysis of factors associated with higher HIV prevalence among MSM in the six regional headquarter towns. The age of the MSM was the only variable that was found to be statistically significant after fitting the multivariable model. The results from the multivariable regression analysis showed that MSM aged 30 years and above had a higher HIV prevalence compared to MSM who were relatively younger. The prevalence of HIV among the MSM aged 30 years was approximately 5 times as high as the prevalence of HIV among MSM aged 17-24 years (aPR=4.6, 95% CI: 1.4-15.2; p<0.05)

**Table 34: Multivariable analysis of factors associated with HIV among MSM**

| **Sociodemographic characteristics** | **Adjusted prevalence ratio from double selection Lasso Poisson Regression Model adjusting for sampling weight from RDS** | **Adjusted Odds ratio from Firth Penalized maximum likelihood logistic regression model adjusting for sampling weight from RDS** |
| --- | --- | --- |
|  | **aPR [95% CI]** | **aOR [95% CI]** |
| **Age in years (N=561)** |  |  |
| 17-24 years | 1 | 1 |
| 25-29 years | 3.61 [0.99-13.11]* | 3.48 [0.90-13.52] |
| 30+ | 4.57 [1.37-15.24]* | 4.62 [1.04-20.38]* |
| **Income (N=533)** |  |  |
| <500000 | 1 | 1 |
| 500000+ | 1.41 [0.53-3.76] | 1.42 [0.48-4.22] |
| **Employment status** |  |  |
| Employed | 1 | 1 |
| Unemployed | 0.56 [0.22-1.45] | 0.57 [0.18-1.76] |
| **Marital status (N=561)** |  |  |
| Married/divorced etc | 1 | 1 |
| Single | 0.57 [0.23-1.39] | 0.53 [0.17-1.61] |
| **Religion (N=560)** |  |  |
| Christian | 1 | 1 |
| Moslem | 0.46 [0.15-1.44] | 0.46 [0.15-1.39] |
| **Province (N=562)** |  |  |
| Northern | 1 | 1 |
| Eastern | 0.92 [0.06-15.00] | 0.94 [0.09-9.48] |
| Southern | 2.71 [0.16-45.85] | 2.72 [0.25-29.45] |
| Western | 6.91 [0.91-52.40] | 5.44 [0.92-32.18] |
| **Ever had vaginal sex** |  |  |
| Yes | 1 | 1 |
| No | 0.41 [0.09-1.86] | 0.46 [0.11-1.98] |

Abbreviation: MSM: Men who have sex with men, aPR: Adjusted Prevalence Ratio, aOR: Adjusted odds ratio; CI: Confidence interval. P-value notation: ***p<0.001, **p<0.01, *p<0.05.

PRISONERS

Sociodemographic Characteristics of inmates in Sierra Leone

This section presents the sociodemograhic characteristics of inmates in the six regional headquarter towns in Sierra Leone. In all, 456 prisoners were enrolled from eight correction centers with an acceptance rate of 100% for bio-behavioural survey interviews, HIV testing, and the provision of blood samples. The average age of the prisoners was 32.4 years with the youngest and oldest prisoners being 17 and 74 years old respectively. About nine in every ten inmates (n=390, 85.5%) interviewed were males and seven out of every ten (n=334, 73.3%) participants had ever attended school. The proportion of prisoners who had ever married/cohabited was 64.3% (n=293) however only 37.9% (n=293) of them were either still married (n=96, 32.8%) or cohabiting (n=15, 5.1%). Muslims formed the predominant religion among the prisoners (n=275, 60.3%). Most of the inmates were recruited from the western area (n=325, 71.3%) with Freetown Male Correctional Center (n=234, 51.3%) contributing to approximately half of the prisoners. Most of the inmates were convicted prisoners (n=358, 78.5%). The detailed distribution of the characteristics of inmates are detailed in Table 1

Table 35: Characteristics of Prisoners in Sierra Leone

|  | **Frequency** | **Percent** |
| --- | --- | --- |
| **Nationality** |  |  |
| Sierra Leone | 441 | 96.71 |
| Liberia | 4 | 0.88 |
| Guinea | 9 | 1.97 |
| Other countries | 2 | 0.44 |
| **Age in years** |  |  |
| **Mean (SD)** | 32.39 (10.72) |  |
| 17-24years | 108 | 23.68 |
| 25-29years | 126 | 27.63 |
| 30-39years | 127 | 27.85 |
| 40+years | 95 | 20.83 |
| **Sex at birth** |  |  |
| Male | 390 | 85.53 |
| Female | 66 | 14.47 |
| **Current gender** |  |  |
| Male | 391 | 85.75 |
| Female | 65 | 14.25 |
| **Ever attended school** |  |  |
| Yes | 334 | 73.25 |
| No | 122 | 26.75 |
| **Highest level of education** |  |  |
| Primary | 91 | 27.25 |
| JSS | 115 | 34.43 |
| SSS/ Technical Vocational | 109 | 32.63 |
| Higher | 19 | 5.69 |
| **Ever married/ Cohabited** |  |  |
| Yes | 293 | 64.25 |
| No | 163 | 35.75 |
| **What is your marital status at the moment?** |  |  |
| Currently Married | 96 | 32.76 |
| Separated | 110 | 37.54 |
| Divorced | 26 | 8.87 |
| Widowed | 17 | 5.8 |
| Cohabitating | 15 | 5.12 |
| Single | 25 | 8.53 |
| No Response | 4 | 1.37 |
| **Religion** |  |  |
| Christian | 180 | 39.47 |
| Moslem | 275 | 60.31 |
| No Religion | 1 | 0.22 |
| **Province** |  |  |
| Northern Province | 40 | 8.77 |
| Eastern Province | 68 | 14.91 |
| Southern Province | 23 | 5.04 |
| Western Area | 325 | 71.27 |
| **District** |  |  |
| Bombali District | 22 | 4.82 |
| Port Loko | 18 | 3.95 |
| Kenema | 37 | 8.11 |
| Kono | 31 | 6.8 |
| Bo | 23 | 5.04 |
| Western Area Urban | 294 | 64.47 |
| Western Area Rural | 31 | 6.8 |
| **Town name** |  |  |
| Makeni | 22 | 4.82 |
| Port Loko | 18 | 3.95 |
| Kenema | 37 | 8.11 |
| Koidu | 31 | 6.8 |
| Bo | 23 | 5.04 |
| Freetown | 294 | 64.47 |
| Waterloo | 31 | 6.8 |
| **Venue** |  |  |
| Makeni Male Correctional Center | 22 | 4.82 |
| Port Loko Correctional Center | 18 | 3.95 |
| Kenema Male Correctional Center | 37 | 8.11 |
| Sefadu Correctional Center | 31 | 6.8 |
| Bo Correctional Center | 23 | 5.04 |
| Freetown Male Correctional Center | 234 | 51.32 |
| Waterloo Simulation Correctional Center | 31 | 6.8 |
| Freetown Female Correctional Center | 60 | 13.16 |
| **Type of respondent** |  |  |
| Remand Prisoners | 60 | 13.16 |
| Convicted Prisoners | 358 | 78.51 |
| Other | 38 | 8.33 |

Abbreviation: SD: Standard deviation

**Sexual history and risk behaviour of prisoners in the six regional headquarter towns**

Table 36 shows the sexual history and risk behaviour of prisoners in Sierra Leone. Approximately 11% (n=49) of the sampled prisoners had previous prison experience with most of them having been incarcerated at least once. The average number of prisoners sleeping together in a cell was 6. Approximately 10% (n=37) of the prisoners had ever had vaginal or anal sex with women while less than three percent (n=9, 2.2%) of them had ever had vaginal/anal sex with men since being detained/incarcerated.

**Table 36: Sexual history and risk behaviour of prisoners in the six regional headquarter towns**

|  | **Frequency** | **Percent** |
| --- | --- | --- |
| **Ever been detained or imprisoned before this time** |  |  |
| Yes | 49 | 10.75 |
| No | 406 | 89.04 |
| Don’t know | 1 | 0.22 |
| **Number of times have you been incarcerated: Median (LQ, UQ)** | 1 (1, 2) |  |
| **Number of other people sharing sleeping quarters with respondent: Median (LQ, UQ)** | 6 (4, 14) |  |
| **Ever had vaginal or anal sex with women since being detained/incarcerated this time** |  |  |
| Yes | 37 | 9.46 |
| No | 354 | 90.54 |
| **Number of female partners have you had vaginal or anal sex during the past six months since you were detained/incarcerated this time: Median (LQ, UQ)** | 3 (1, 5) |  |
| **Have you had vaginal sex or anal sex with men since being detained/incarcerated** |  |  |
| Yes | 9 | 2.15 |
| No | 410 | 97.85 |
| **Number of male partners have you had vaginal or anal sex during the past six months since you were detained/incarcerated this time** |  |  |
| Zero | 2 | 22.22 |
| One | 7 | 77.78 |
| **Have you had anal sex during the past six months since you were incarcerated this time** |  |  |
| Yes | 5 | 10.87 |
| No | 41 | 89.13 |
| **How many male partners have you had anal sex during the past six months since incarceration** |  |  |
| Zero | 2 | 40 |
| One | 2 | 40 |
| Two | 1 | 20 |
| **While incarcerated during the past six months, have you been forced or coerced to have sex** |  |  |
| Yes | 5 | 1.1 |
| No | 451 | 98.9 |
| While incarcerated in the past six months, have you been forced or coerced to have sex by another prisoner |  |  |
| Yes | 3 | 0.66 |
| No | 453 | 99.34 |
| While incarcerated during the past six months, have you had sex with prison staff/authority figure |  |  |
| Yes | 3 | 0.66 |
| No | 452 | 99.12 |
| Don’t know | 1 | 0.22 |
| While incarcerated in the past six months, have you had an object inserted into your anus by another prisoner or staff again your will |  |  |
| Yes | 5 | 1.1 |
| No | 451 | 98.9 |
| While incarcerated during the past six months, have you had sex with someone for money, goods, access, protection or services? |  |  |
| Yes | 1 | 0.22 |
| No | 455 | 99.78 |
| Are you able to obtain condoms in this prison? |  |  |
| No | 453 | 99.34 |
| Don't know | 2 | 0.44 |
| No Response | 1 | 0.22 |
| **Have you used drugs to get high while in prison this time?** |  |  |
| Yes | 62 | 13.6 |
| No | 393 | 86.18 |
| Don't know | 1 | 0.22 |
| **Have you injected drugs while in prison this time?** |  |  |
| Yes | 13 | 2.85 |
| No | 443 | 97.15 |
| **In the last six months since you were incarcerated this time, have you ever shared blades to shave or cut hair** |  |  |
| Yes | 180 | 39.47 |
| No | 273 | 59.87 |
| Don't know | 3 | 0.66 |
| **In the last six months while incarcerated, have you ever been tattooed?** |  |  |
| Yes | 13 | 2.85 |
| No | 442 | 96.93 |
| Don't know | 1 | 0.22 |
| **In the last six months while incarcerated, have you ever made a piercing or put in an earring?** |  |  |
| Yes | 40 | 8.77 |
| No | 416 | 91.23 |

**HIV knowledge, opinion, attitude, and prevention of HIV/AIDS**

Table 37 shows estimates of knowledg**e**, opinion, attitude, and prevention of HIV/AIDS amongst prisoners in Sierra Leone. The average knowledge score of the prisoners on HIV/AIDs related indicators was 6.8 based on the minimum and maximum attainable scores of 0 and 13 respectively. Three-fourth (75%) of the sampled inmates correctly stated the possibility of a healthy-looking person contracting HIV. Details of prisoners' knowledge, opinion, attitude, and prevention of HIV/AIDS are presented in Table 3.

**Table 37: HIV knowledge, opinion, attitude, and prevention of HIV/AIDS**

|  | **Frequency** | **Percent** |
| --- | --- | --- |
| **Can people reduce their chance of getting HIV by having just one uninfected sex** |  |  |
| Correct response | 100 | 21.93 |
| Wrong response | 303 | 66.45 |
| Don’t know | 53 | 11.62 |
| **Can people get HIV from mosquito bites?** |  |  |
| Wrong response | 186 | 40.79 |
| Correct response | 179 | 39.25 |
| Don’t know | 91 | 19.96 |
| **Can people reduce their chance of getting HIV by using a condom every time they have sex** |  |  |
| Correct response | 312 | 68.42 |
| Wrong response | 78 | 17.11 |
| Don’t know | 65 | 14.25 |
| No Response | 1 | 0.22 |
| **Can a person get HIV by sharing food with someone who is infected?** |  |  |
| Correct response | 244 | 53.51 |
| Wrong response | 157 | 34.43 |
| Don’t know | 55 | 12.06 |
| **Can people get HIV because of supernatural means (juju, witchcraft, black magic )** |  |  |
| Correct response | 323 | 70.83 |
| Wrong response | 65 | 14.25 |
| Don’t know | 68 | 14.91 |
| **Is it possible for a healthy-looking person to have HIV?** |  |  |
| Correct response | 342 | 75.00 |
| Wrong response | 63 | 13.82 |
| Don'T Know | 51 | 11.18 |
| **Can a person get HIV by getting injections with a needle that was already used by someone else** |  |  |
| Correct response | 411 | 90.13 |
| Wrong response | 21 | 4.61 |
| Don’t know | 24 | 5.26 |
| **Can an infected mother transmit HIV to her unborn child during pregnancy?** |  |  |
| Correct response | 257 | 56.36 |
| Wrong response | 108 | 23.68 |
| Don’t know | 91 | 19.96 |
| **Can a woman with HIV transmit the virus to her newborn child during delivery?** |  |  |
| Correct response | 246 | 53.95 |
| Wrong response | 121 | 26.54 |
| Don’t know | 89 | 19.52 |
| **Can a woman with HIV transmit the virus to her newborn child through breastfeeding** |  |  |
| Correct response | 296 | 64.91 |
| Wrong response | 91 | 19.96 |
| Don’t know | 68 | 14.91 |
| No Response | 1 | 0.22 |
| **Are there any special drugs that a doctor or a nurse can give to a woman infected with HIV to reduce risk of transmission to the baby** |  |  |
| Correct response | 192 | 42.11 |
| Wrong response | 156 | 34.21 |
| Don’t know | 108 | 23.68 |
| **Have you heard about special antiretroviral drugs (e.g. ARV, nevirapine, zidovudine)** |  |  |
| Yes | 205 | 44.96 |
| No | 192 | 42.11 |
| Don’t know | 59 | 12.94 |
| **Do you know anyone who has died from AIDS?** |  |  |
| Yes | 123 | 26.97 |
| No | 333 | 73.03 |
| **Who died from HIV** |  |  |
| Friend | 35 | 28.46 |
| Neighbor | 48 | 39.02 |
| Colleague | 10 | 8.13 |
| Sexual partner | 3 | 2.44 |
| Other | 7 | 5.69 |
| **Did you help to care for him/her before be he/she died** |  |  |
| Yes | 35 | 28.46 |
| No | 87 | 70.73 |
| No response | 1 | 0.81 |
| **Source of information on STDs, AIDS, or sexual risk** |  |  |
| Health facility | 122 | 26.75 |
| On the radio | 149 | 32.68 |
| On the tv | 125 | 27.41 |
| Newspapers | 3 | 0.66 |
| Other (Specify) | 165 | 36.18 |
| **whom have you talked about STDs, AIDS, or sexual risk** |  |  |
| Health worker | 124 | 27.19 |
| Friend/neighbor/colleague | 170 | 37.28 |
| Sexual partner | 3 | 0.66 |
| Other (Specify) | 192 | 42.11 |
| **Overall Knowledge level on HIV/AIDS** |  |  |
| Low(0-6/13) | 190 | 41.67 |
| Moderate(7-10/13) | 244 | 53.51 |
| High(11-13/13) | 22 | 4.82 |

**Coverage of prevention programs**

Approximately 63.2% (n=288) prisoners were aware of civil society or NGOs that deliver non-medical assistance or advice but less than one-fifth (n=67, 14.7%) of the prisoners attended meetings to discuss HIV and/or AIDs within the last 18 months. About a quarter (n=122, 26.8%) of the prisoners have been in contact with a health peer educator in the community in the last 18 months. Some of the services received from the peer educators included general HIV/STI prevention/transmission information (n=85, 69.7%), referral for STI treatment (n=15, 12.3%), and condoms (n=7, 5.7%). Details of the coverage of prevention programs are shown in Table 38.

**Table 38: Exposure to interventions**

|  | **Frequency** | **Percent** |
| --- | --- | --- |
| **Are you aware of any civil society or any organization(s) that deliver nonmedical assistance?** |  |  |
| Yes | 288 | 63.16 |
| No | 148 | 32.46 |
| Don’t know | 19 | 4.17 |
| No Response | 1 | 0.22 |
| **During the last 18 months, have you attended any meetings to discuss HIV and/or AIDs** |  |  |
| Yes | 67 | 14.69 |
| No | 384 | 84.21 |
| Don’t know | 5 | 1.1 |
| **In the last 18 months, have you been in contact with a health peer educator in the community?** |  |  |
| Yes | 122 | 26.75 |
| No | 324 | 71.05 |
| Don’t know | 10 | 2.19 |
| **In the last 18 months, how many times have you been in contact with the peer educator** |  |  |
| Median (LQ, UQ) | 2 (1, 5) |  |
| **Services received from a peer educator** |  |  |
| General HIV/STI prevention/transmission information | 85 | 69.67 |
| Condoms | 7 | 5.74 |
| Referral for STI treatment | 15 | 12.3 |
| **Did the peer educator refer you for medical care at a health center?** |  |  |
| Yes | 42 | 34.43 |
| No | 78 | 63.93 |
| Don’t know | 2 | 1.64 |
| **Did you go to the referred site to receive medical care?** |  |  |
| Yes | 26 | 57.78 |
| No | 18 | 40 |
| Don’t know | 1 | 2.22 |
| **Information heard or seen any messages about HIV, AIDS, sexually transmitted diseases, or condoms** |  |  |
| Use of condoms and lubricants | 237 | 63.03 |
| Importance of HIV testing | 124 | 32.98 |
| Importance of reducing the number of sexual partners | 84 | 22.34 |
| Importance of STI screening and management | 27 | 7.18 |
| Reporting on sexual and gender-based violence | 16 | 4.26 |
| Other (Specify) | 102 | 27.13 |
| **Sources of information on HIV/AIDS** |  |  |
| Radio | 146 | 38.83 |
| TV | 156 | 41.49 |
| Newspaper/booklets | 5 | 1.33 |
| Religious leaders | 7 | 1.86 |
| NGOS/CSOS | 19 | 5.05 |
| Teachers | 3 | 0.8 |
| Health workers | 91 | 24.2 |
| Friends/peers | 60 | 15.96 |
| Other (Specify) | 35 | 9.31 |

**Information on STIs**

Table 39 shows the information on previous HIV testing and perception of risk. The majority of prisoners reported to have ever tested for HIV (n=64, 61.5%) and the tests were mostly (n=41, 64.1%) done at a Government Hospital/Clinic/Health Center. Of those prisoners who have had HIV tests indicated that they tested positive for HIV (n=4, 6.3%). Among the 39 prisoners who have not had the test, the common reasons were because they were afraid of knowing they may be HIV-Positive, feel they are not at risk of getting HIV and some also didn't have time/too busy. Most Prisoners who had been tested indicated to be very satisfied with the quality of services provided at the place where they had the HIV test (n=52, 78.8%). Details of Previous HIV testing and perception of risk can be found in Table 39.

**Table 39:** **Previous HIV testing and perception of risk**

|  | **Frequency** | **Percent** |
| --- | --- | --- |
| **Places you know where one can get tested for HIV** |  |  |
| Government Hospital/Clinic/Health Center | 48 | 10.53 |
| Private Facility/Clinic | 6 | 1.32 |
| Work/Employer | 5 | 1.1 |
| Don't know | 30 | 6.58 |
| **Ever been tested for HIV** |  |  |
| Yes | 64 | 61.54 |
| No | 39 | 37.5 |
| Don’t know | 1 | 0.96 |
| **Why not have an HIV test** |  |  |
| I always use condoms | 1 | 2.5 |
| Not at risk of getting HIV | 3 | 7.5 |
| Didn't have time/too busy | 3 | 7.5 |
| Afraid of knowing I may be HIV-Positive | 4 | 10 |
| Lack of confidentiality | 1 | 2.5 |
| Other (specify) | 7 | 17.5 |
| **Where had the last test done** |  |  |
| Government Hospital/Clinic/Health Center | 41 | 64.06 |
| Private Facility Clinic | 5 | 7.81 |
| Work/Employer | 1 | 1.56 |
| Other Specify | 16 | 25 |
| Don'T Know | 1 | 1.56 |
| **Why got this last test** |  |  |
| Wanted to know my HIV status | 44 | 68.75 |
| Employer requested the test | 1 | 1.56 |
| I felt sick | 4 | 6.25 |
| Advised by a health worker | 14 | 21.88 |
| Advised by a peer educator | 4 | 6.25 |
| Other (specify) | 8 | 12.12 |
| **What was the result of your last HIV test?** |  |  |
| Hiv-Negative | 58 | 90.63 |
| Hiv-Positive | 4 | 6.25 |
| I Didn'T Get The Result | 2 | 3.13 |
| **If you didn't get your result, why not?** |  |  |
| Don'T Know | 2 | 100 |
| **What do you think your chances of transmitting HIV to a partner is?** |  |  |
| No Chance | 1 | 16.67 |
| Small Chance | 2 | 33.33 |
| Great Chance | 1 | 16.67 |
| Don'T Know | 2 | 33.33 |
| **What do you think are your chances of getting HIV?** |  |  |
| No Chance | 58 | 59.18 |
| Small Chance | 12 | 12.24 |
| Moderate Chance | 2 | 2.04 |
| Great Chance | 3 | 3.06 |
| Don'T Know | 22 | 22.45 |
| No Response | 1 | 1.02 |
| **Don't use condoms** |  |  |
| Yes | 1 | 100 |
| **Had injuries/cuts** |  |  |
| Yes | 3 | 100 |
| Don't know |  |  |
| Yes | 1 | 100 |
| **What do you think your HIV status is today?** |  |  |
| Hiv-Negative | 64 | 65.31 |
| Hiv-Positiive | 1 | 1.02 |
| Don'T Know | 32 | 32.65 |
| No Response | 1 | 1.02 |
| **How satisfied were you with the quality of services provided at the place where** |  |  |
| Very Satisfied | 52 | 78.79 |
| Satisfied | 10 | 15.15 |
| A Little Satisfied | 2 | 3.03 |
| Don'T Know | 1 | 1.52 |
| No Response | 1 | 1.52 |
| **At anytime during your most recent counseling and testing experience, did you re** |  |  |
| Yes | 5 | 71.43 |
| No | 1 | 14.29 |
| No Response | 1 | 14.29 |
| Other (specify) | 1 | 100 |
| **Did you feel that a counselor or health care provider reacted to you in a negatively** |  |  |
| No | 5 | 100 |

Prevalence and sociodemographic determinants of HIV among Prisoners in Sierra Leone

This section presents the estimated prevalence of HIV and the corresponding 95% confidence interval estimate among prisoners in the six regional headquarter towns in Sierra Leone.

The results from the logit-transformed confidence intervals with venue-based cluster robust standard error showed that the overall prevalence of HIV among prisoners in the six regional headquarter towns was estimated to be 3.7% (95%CI:1.4–9.6). Among all the sociodemographic characteristics studied, the HIV prevalence estimates differed significantly by only the sex of the prisoner. HIV prevalence was about six times higher in females than males (Females - 12.1% vs Males - 2.3%). All HIV cases were found among only convicted prisoners. HIV was highly prevalent in Freetown Female Correctional Center (11.7%; 95% CI: 5.7 - 22.6), Makeni Male Correctional Center (4.6%, 95% CI: 0.6 - 26.3), Kenema Male Correctional Center 8.1%[95%CI: 2.6 - 22.4], Bo Correctional Center (4.4%, 95% CI: 0.6 - 25.3) and Freetown Male Correctional Center (2.1%, 95% CI: 0.9 - 5.0) only. Table 40 shows the HIV prevalence estimates, unadjusted prevalence ratio, and odds ratios by sociodemographic characteristics of prisoners in Sierra Leone.

Table 40:Prevalence and sociodemographic determinants of HIV among Prisoners in Sierra Leone

|  | HIV status | Unadjusted prevalence ratio from Modified Poisson Regression Model | Unadjusted Odds ratio from the binary logistic regression model |
| --- | --- | --- | --- |
|  | HIV+ (%) [95% CI] | uPR [95% CI] | uOR [95% CI] |
| **Overall** | 3.73[1.40 – 9.57] |  |  |
| Age |  |  |  |
| 18-24years | 6.48[3.12 - 13] | 1 | 1 |
| 25-29years | 0.79[0.11 - 5.44] | 0.12[0.02 - 1] | 0.12[0.01 - 0.95] |
| 30-39years | 3.15[1.18 - 8.11] | 0.49[0.14 - 1.66] | 0.47[0.13 - 1.65] |
| 40+years | 5.26[2.2 - 12.05] | 0.81[0.26 - 2.56] | 0.8[0.25 - 2.61] |
| **Marital status** |  |  |  |
| Never married/cohabited | 4.49[2.72 - 7.32] | 1 | 1 |
| Others | 1.64[0.41 - 6.34] | 0.37[0.08 - 1.6] | 0.35[0.08 - 1.57] |
| **Highest level of education** |  |  |  |
| None | 1.64[0.41 - 6.34] | 1 | 1 |
| Primary | 4.4[1.66 - 11.16] | 2.68[0.49 - 14.64] | 2.76[0.49 - 15.4] |
| JSS | 3.48[1.31 - 8.92] | 2.12[0.39 - 11.58] | 2.16[0.39 - 12.04] |
| SSS/ Technical Vocational/ higher | 5.47[2.62 - 11.05] | 3.34[0.69 - 16.06] | 3.47[0.71 - 17.05] |
| **Marital status** |  |  |  |
| Never married/cohabited | 3.07[1.6 - 5.81] | 1 | 1 |
| Others | 4.91[2.47 - 9.52] | 1.6[0.62 - 4.14] | 1.63[0.62 - 4.31] |
| Sex at birth |  |  |  |
| Male | 2.31[1.2 - 4.38] | 1 | 1 |
| Female | 12.12[6.17 - 22.45] | 5.25[2.03 - 13.61]* | 5.84[2.17 - 15.74]* |
| **Religion** |  |  |  |
| Christian | 4.44[2.23 - 8.65] | 1 | 1 |
| Moslem | 3.27[1.71 - 6.18] | 0.74[0.28 - 1.91] | 0.73[0.28 - 1.92] |
| **Regions** |  |  |  |
| Northern | 2.5[0.35 - 15.8] | 1 | 1 |
| Eastern | 4.41[1.43 - 12.84] | 1.76[0.18 - 16.97] | 1.8[0.18 - 17.91] |
| Southern | 4.35[0.61 - 25.32] | 1.74[0.11 - 27.8] | 1.77[0.11 - 29.76] |
| Western | 3.69[2.11 - 6.4] | 1.48[0.19 - 11.36] | 1.5[0.19 - 11.81] |
| **District** |  |  |  |
| Bombali | 4.55[0.63 - 26.25] | 1 | 1 |
| Port Loko | 0.00 | _ | _ |
| Kenema | 8.11[2.63 - 22.37] | 1.78[0.19 - 17.15] | 1.85[0.18 - 19] |
| Kono | 0.00 | _ | _ |
| Bo | 4.35[0.61 - 25.32] | 0.96[0.06 - 15.29] | 0.95[0.06 - 16.27] |
| Western Urban | 4.08[2.33 - 7.06] | 0.9[0.12 - 6.91] | 0.89[0.11 - 7.21] |
| Western Rural | 0.00 | _ | _ |
| **Town** |  |  |  |
| Makeni | 4.55[0.63 - 26.25] | 1 | 1 |
| Port Loko | 0.00 | _ | _ |
| Kenema | 8.11[2.63 - 22.37] | 1.78[0.19 - 17.15] | 1.85[0.18 - 19] |
| Koidu | 0.00 | _ | _ |
| Bo | 4.35[0.61 - 25.32] | 0.96[0.06 - 15.29] | 0.95[0.06 - 16.27] |
| Freetown | 4.08[2.33 - 7.06] | 0.9[0.12 - 6.91] | 0.89[0.11 - 7.21] |
| Waterloo and Grafton | 0.00 | _ | _ |
|  |  |  |  |
| **Prison centre** |  |  |  |
| Makeni Male Correctional Center | 4.55[0.63 - 26.25] | 1[0 - 0] | 1 |
| Port Loko Correctional Center | 0.00 | _ | _ |
| Kenema Male Correctional Center | 8.11[2.63 - 22.37] | 1.78[0.19 - 17.14] | 1.85[0.18 - 19] |
| Sefadu Correctional Center | 0.00 | _ | _ |
| Bo Correctional Center | 4.35[0.61 - 25.32] | 0.96[0.06 - 15.29] | 0.95[0.06 - 16.27] |
| Freetown Male Correctional Center | 2.14[0.89 - 5.04] | 0.47[0.05 - 4.02] | 0.46[0.05 - 4.11] |
| Waterloo Simulation Correctional Center | 0.00 | _ | _ |
| Freetown Female Correctional Center | 11.67[5.65 - 22.55] | 2.57[0.32 - 20.85] | 2.77[0.32 - 23.94] |

- Estimation not possible because the prevalence of HIV was estimated to be 0.0%

**Prevalence of HIV by knowledge, opinions, and attitudes among prisoners in Sierra Leone**

Table 41 presents the prevalence of HIV by knowledge, opinions, and attitudes among prisoners in Sierra Leone. In most cases, HIV prevalence was higher among prisoners who provided correct responses compared to those who provided wrong responses to HIV-related questions. Generally, the prevalence of HIV among prisoners with higher scores in HIV-related knowledge was 22.7%, (95% CI:5.02-62.09) compared to those inmates who had low knowledge scores on HIV-related questions (1.6, 95% CI: 0.4- 6.4). Table 41 provides details of the prevalence of HIV by knowledge, opinions, and attitudes among prisoners in Sierra Leone.

Table 41: Prevalence of HIV by knowledge, opinions, and attitudes among prisoners in Sierra Leone

| **Knowledge, opinions, and attitudes** | HIV status | Unadjusted prevalence ratio from Modified Poisson Regression Model | Unadjusted Odds ratio from the binary logistic regression model |
| --- | --- | --- | --- |
|  | HIV+ (%) [95% CI] | uPR [95% CI] | uOR [95% CI] |
| **Can people reduce their chance of getting HIV by having just one uninfected sex partner who has no other sex partners?** |  |  |  |
| Wrong response | 3.93[2.34 - 6.54] | 1 | 1 |
| Correct response | 3.00[0.97 - 8.91] | 0.76[0.28 - 2.1] | 0.76[0.27 - 2.14] |
| **Can people get HIV from mosquito bites?** |  |  |  |
| Wrong response | 2.96[1.49 - 5.82] | 1 | 1 |
| Correct response | 4.84[2.53 - 9.05] | 1.63[0.84 - 3.18]* | 1.67[0.84 - 3.29]* |
| **Can people reduce their chance of getting HIV by using a condom every time they have sex?** |  |  |  |
| Wrong response | 0.69[0.1 - 4.78] | 1 | 1 |
| Correct response | 5.13[3.16 - 8.22] | 7.38[1.64 - 33.35]* | 7.73[1.74 - 34.36]* |
| **Can a person get HIV by sharing food with someone who is infected?** |  |  |  |
| Wrong response | 2.36[0.98 - 5.55] | 1 | 1 |
| Correct response | 4.92[2.81 - 8.47] | 2.09[0.52 - 8.31] | 2.14[0.51 - 8.92] |
| **Can people get HIV because of supernatural means (juju, witchcraft, black magic etc)?** |  |  |  |
| Wrong response | 3.76[1.57 - 8.73] | 1 | 1 |
| Correct response | 3.72[2.12 - 6.44] | 0.99[0.44 - 2.23] | 0.99[0.42 - 2.3] |
| **Is it possible for a healthy-looking person to have HIV?** |  |  |  |
| Wrong response | 1.75[0.44 - 6.76] | 1 | 1 |
| Correct response | 4.39[2.66 - 7.16] | 2.5[0.6 - 10.41] | 2.57[0.59 - 11.18] |
| **Can an infected mother transmit HIV to her unborn child during pregnancy?** |  |  |  |
| Wrong response | 2.01[0.75 - 5.24] | 1 | 1 |
| Correct response | 5.06[2.96 - 8.53] | 2.52[0.89 - 7.09] | 2.6[0.88 - 7.69] |
| **Can a woman with HIV transmit the virus to her newborn child during delivery?** |  |  |  |
| Wrong response | 2.38[0.99 - 5.6] | 1 | 1 |
| Correct response | 4.88[2.79 - 8.4] | 2.05[0.69 - 6.06] | 2.1[0.68 - 6.55] |
| **Can a woman with HIV transmit the virus to her newborn child through breastfeeding?** |  |  |  |
| Wrong response | 1.25[0.31 - 4.87] | 1 | 1 |
| Correct response | 5.07[3.07 - 8.24] | 4.05[0.53 - 31.21] | 4.22[0.51 - 34.67] |
| **Are there any special drugs that a doctor or a nurse can give to a woman infected with HIV to reduce the risk of transmission to the baby?** |  |  |  |
| Wrong response | 3.41[1.78 - 6.43] | 1 | 1 |
| Correct response | 4.17[2.09 - 8.12] | 1.22[0.46 - 3.25] | 1.23[0.45 - 3.38] |
| **Are there any special drugs that a doctor or a nurse can give to a woman infected with HIV to reduce the risk of transmission to the baby?** |  |  |  |
| Wrong response | 2.39[1.08 - 5.23] | 1 | 1 |
| Correct response | 5.37[2.99 - 9.44] | 2.24[1.47 - 3.43]* | 2.32[1.51 - 3.54]* |
| **Have you heard about special antiretroviral drugs (e.g. ARV, nevirapine, zidovudine, lamivudine) that people infected with HIV can get from a doctor or a nurse to help them live longer?** |  |  |  |
| No | 2.7[1.41 - 5.12] | 1 | 1 |
| Yes | 6.5[3.28 - 12.49] | 2.41[1.4 - 4.13]* | 2.5[1.45 - 4.32]* |
| **Overall knowledge, opinion and attitudes towards HIV/AIDS** |  |  |  |
| Low (0-6/13) | 1.58[0.37 - 6.40] | 1 | 1 |
| Moderate (7-10/13) | 3.69[1.57 - 8.43] | 2.34[0.83 - 6.54] | 2.39[0.84 - 6.81] |
| High(11-13/13) | 22.73[5.02 - 62.09] | 14.39[7.43 - 27.85] | 18.33[8.38 - 40.11] |

**Prevalence of HIV among Prisoners by exposure to the intervention**

The prevalence of HIV varied between prisoners who had contact with health peer navigators in the last 18 months on HIV and AIDS-related services. That is, the prevalence of HIV among prisoners who had contact with health peer navigators in the last 18 months on HIV and AIDS-related services was twice as high as the prevalence of HIV among those who did not have any of such contact (had contact: 5.7, 95% CI: 2.4 - 13.1) versus no contact: 2.8, 95% CI: 0.98 - 7.62). However, interventions, like attending meeting (s) to discuss HIV and /or AIDS in the last 18 months and being aware of any civil society or organizations that deliver non-medical assistance or advice, did not have a statistically significant effect on HIV prevalence (Table 42).

Table 42: Prevalence of HIV among Prisoners by exposure to the intervention

| Exposure to intervention | HIV status | Unadjusted prevalence ratio from Modified Poisson Regression Model | Unadjusted odds ratio from the binary logistic regression model |
| --- | --- | --- | --- |
|  | HIV+ (%) [95% CI] | uPR [95% CI] | uOR [95% CI] |
| **Are you aware of any civil society or any organization(s) that deliver non-medical HIV services** |  |  |  |
| Yes | 3.82[0.78 - 16.69] | 1 | 1 |
| No | 3.38[1.7 - 6.62] | 0.88[0.21 - 3.65] | 0.88[0.2 - 3.84] |
| **During the last 18 months, have you attended any meetings to discuss HIV and/or AIDS** |  |  |  |
| Yes | 2.99[0.71 - 11.68] | 1 | 1 |
| No | 3.91[1.2 - 12.01] | 1.31[0.22 - 7.62] | 1.32[0.21 - 8.18] |
| **Contact with health peer navigator in the last 18 months on HIV and AIDS-related services** |  |  |  |
| Yes | 5.74[2.4 - 13.11] | 1 | 1 |
| No | 2.78[0.98 - 7.62] | 0.48[0.33 - 0.71]* | 0.47[0.32 - 0.69]* |

**Factors associated with HIV among Prisoners in Sierra Leone: a multivariable regression analysis**

The results from the multivariable modified Poisson regression model showed that sex of prisoners, educational level, marital status, age, and HIV and AIDs related knowledge, were associated with higher HIV prevalence among prisoners in Sierra Leone. The prevalence of HIV among female prisoners was approximately 6 times as high as the prevalence of HIV among male prisoners (adjusted prevalence ratio; aPR=5.51, 95% CI: 4.2 - 7.22, p<0.05; Table 9). The prevalence of HIV among prisoners who have ever been married or currently been in a relationship was approximately 3 times the prevalence of HIV among prisoners who have never been married or cohabiting ( aPR=2.9, 95% CI:2.1 – 4.0, p<0.05; Table 9). HIV prevalence among prisoners with high HIV-related knowledge was 10 times as high as the prevalence of HIV among prisoners with low HIV-related knowledge (aPR=9.8, 95% CI:7.2 - 13.4, Table 9). Prisoners aged 40 years and above had about 3 times higher prevalence of HIV compared to those aged 17-24years old (aPR=2.8, 95%CI:1.0- 7.3). Prisoners with SSS/ Technical Vocational/ higher had about 2 times higher prevalence of HIV compared to those without any formal education (aPR=2.1, 95%CI: 1.4 - 2.9; Table 43)

Table 43: Factors associated with HIV among Prisoners in Sierra Leone: a multivariable regression analysis

| Factors | Adjusted prevalence ratio from Modified Poisson Regression Model | Adjusted Odds ratio from the binary logistic regression model |
| --- | --- | --- |
|  | aPR [95% CI] | aOR [95% CI] |
| **Sex at birth** |  |  |
| Male | 1 | 1 |
| Female | 5.51[4.2 - 7.22]* | 7.85[5.62 - 10.98]* |
| **Educational level** |  |  |
| None | 1 | 1 |
| Primary | 2.15[0.65 - 7.1] | 2.31[0.57 - 9.26] |
| JSS | 2.07[0.75 - 5.67] | 2.24[0.76 - 6.62] |
| SSS/ Technical Vocational/ higher | 2.05[1.43 - 2.95]* | 2.54[1.64 - 3.94]* |
| **Ever married/Cohabited** |  |  |
| Yes | 1 | 1 |
| No | 2.88[2.08 – 4.00]* | 3.52[2.26 - 5.49]* |
| **Age in years** |  |  |
| 17-24years | 1 | 1 |
| 25-29years | 0.18[0.05 - 0.63] | 0.15[0.05 - 0.49] |
| 30-39years | 1[0.23 - 4.31] | 0.98[0.17 - 5.7] |
| 40+years | 2.75[1.04 - 7.27]* | 3.48[1.07 - 11.32]* |
| **HIV Knowledge Level** |  |  |
| Low (0-6/13) | 1 | 1 |
| Moderate (7-10/13) | 2.73[1.13 - 6.62]* | 3.08[1.23 - 7.72]* |
| High(11-13/13) | 9.79[7.17 - 13.37]* | 16.77[8.48 - 33.16]* |

**PEOPLE WHO INJECT DRUGS (PWID)**

**Sociodemographic characteristics of PWID in the six regional headquarter towns**

A total of 1155 PWID were interviewed. The average age of PWIDs in the six regional headquarter towns is 26 years (youngest=17 years, oldest=71 years). Almost half (48.5%, n=560) of the PWIDs were aged between 16-24 years and vast majority of the PWIDs were males at birth (92.7%, n=1070). More than half 54.3% (n=626) of the PWIDs had attained Senior High School certificate and a high majority of them representing 72.8% (n=385) reported a monthly income of five hundred thousand Sierra Leonean Leones (SLL500,000) or lower. A little over half (52.9%, n=379) of the PWIDs reported being unemployed and most of them also reported being never married (62.6%, n=722). About two out of three (68.3% n=788) were affiliated to the Moslem faith. In terms of ethnic composition, the relative majority were either Temne (34.5%, n=398) or belonged to the other ethnic groups (38.9%, n=448) with the rest being made up of all Mende ethnic group. Nearly, one out of four (23.6%, n=273) PWIDs reside Western Area Urban district with the least of the PWIDs (9.5%, n=110) interviewed residing in the Western Area Rural. Expectedly, the data also shows that one-third (33.2%, n=383) of the PWIDs reside in the Western province followed by 28.7% residing in the Northern province, 24.5% residing in Eastern province and 13.7% residing in the Southern province.

**Table 44: Sociodemographic characteristics of PWID in the six regional headquarter towns**

| **Sociodemographic characteristics** | **Frequency** | **Percent** |
| --- | --- | --- |
| **Age in years (N=1154)** |  |  |
| 18-24 years | 560 | 48.53 |
| 25-29 years | 337 | 29.20 |
| 30+ | 257 | 22.27 |
| **Sex at birth (N=1154)** |  |  |
| Male | 1070 | 92.72 |
| Female | 84 | 7.28 |
| **Education level (N=1153)** |  |  |
| None/primary | 193 | 16.74 |
| JHS | 254 | 22.03 |
| SHS/Tech | 626 | 54.29 |
| Higher | 80 | 6.94 |
| **Income (N=529)** |  |  |
| <500000 | 385 | 72.78 |
| 500000+ | 144 | 27.22 |
| **Employment status (N=1152)** |  |  |
| Employed | 543 | 47.14 |
| Unemployed | 609 | 52.86 |
| **Marital status (N=1154)** |  |  |
| Married/divorced etc | 432 | 37.44 |
| Single | 722 | 62.56 |
| **Religion (N=1154)** |  |  |
| Christian | 366 | 31.72 |
| Moslem | 788 | 68.28 |
| **Ethnicity (N=1153)** |  |  |
| Mende | 307 | 26.63 |
| Temne | 398 | 34.52 |
| Others | 448 | 38.86 |
| **District (N=1155)** |  |  |
| Bombali District | 159 | 13.77 |
| Port Loko | 172 | 14.89 |
| Kenema | 150 | 12.99 |
| Kono | 133 | 11.52 |
| Bo | 158 | 13.68 |
| Western Area Urban | 273 | 23.64 |
| Wester Area Rural | 110 | 9.52 |
| **Province (N=1155)** |  |  |
| Northern | 331 | 28.66 |
| Eastern | 283 | 24.50 |
| Southern | 158 | 13.68 |
| Western | 383 | 33.16 |

**Sexual history of PWID**

Table 45 shows the sexual history of PWIDs in Sierra Leone covered by the study. Almost all (98.8%, n=1057) of the PWIDs have ever had a vaginal sex and relative minority made up of 30.0% (n=321) have also ever had anal sex. Of the PWIDs who have had vaginal sex, 44.9% (n=480) reported of not having had sex in the last three months of the study, 27.2% and 27.9% have had sex with only one and two or more women in the last three months preceding the survey respectively. Among the 1025 PWIDs who had vaginal sex with one or more women majority of them, made up of 41.0% (n=423) did not use any condom at all; the rest reported using condom on only one occasion (34.5%: n=354) or two or more occasions (24.2%: n=248). Of the PWIDs who reported having had anal sex, the vast majority (95.9%: n=1106) have never had anal sex with a man. Nearly 77.8% of the PWIDs experienced first anal sex with a man when they were less than 18 years. About 41.1% have never tested for HIV. Nearly all the PWIDs (99.0%) who have ever tested for HIV know their HIV status with about 97.3% tested as HIV negative and 1.8% tested as HIV positive.

**Table 46: Sexual history of PWID**

| **Sexual history of PWID** | **Frequency** | **Percent** |
| --- | --- | --- |
| **Ever had a vaginal sex (N=1070)** |  |  |
| Yes | 1057 | 98.79 |
| No | 13 | 1.21 |
| **Ever had anal sex with a woman (N=1070)** |  |  |
| Yes | 321 | 30.00 |
| No | 749 | 70.00 |
| **Number of women you had vaginal sex with in the last three month (N=1070)** |  |  |
| None | 480 | 44.86 |
| Only 1 | 291 | 27.20 |
| 2 or more | 299 | 27.94 |
| **Of these, how many did you not use condom (N=1025)** |  |  |
| None | 423 | 41.27 |
| Only 1 | 354 | 34.54 |
| 2 or more | 248 | 24.20 |
| **Ever had anal sex with a man (N=1153)** |  |  |
| Yes | 47 | 4.08 |
| No | 1106 | 95.92 |
| **Age at first anal sex with a man (N=81)** |  |  |
| Less than 18 years | 63 | 77.78 |
| 18+ | 18 | 22.22 |
| **Ever been tested for HIV (N=1150)** |  |  |
| No | 473 | 41.13 |
| Yes | 677 | 58.87 |
| **Do you know the results of your last HIV test (N=671)** |  |  |
| HIV Negative | 653 | 97.32 |
| HIV Positive | 12 | 1.79 |
| Indeterminate | 4 | 0.60 |
| I did not get the results | 2 | 0.30 |

**Sexual and behavioural risk factors and violence against PWID**

The use of condoms is known to be among the efficacious means to only preventing the transmission of HIV and other STIs but also unwanted pregnancy. The effectiveness of condom as preventive tool is dependent on regular, effective and correct usage. Table 47 presents the sexual and behavioural risk factors of PWID in the six regional headquarter towns in Sierra Leone. Approximately 91% (n=978) PWID have never put the condom on after they have already started having sex in the past 6 months during sexual intercourse. About 5% (n=49) of PWID experience condom break or leak during sexual intercourse in the past 6 months. Approximately 17% (n=184) reported that they have taken off the condom during sexual intercourse before they were finished having sex. Among PWID respondents, 17.1%(n=184) indicated that they or their partners ever take the condom off before finishing having sex in the last 6 months preceding the survey. Condom ever slipping off during sex or while pulling out happened among 3.8% of PWID respondents. There is low level utilization of lubricant during vaginal or anal sex among PWID respondents. The results indicate that 10.6% of PWID respondents used lubricant always/usually during sex (vaginal or anal) with 25.6% using lubricant sometimes/rarely. Majority (64.8%) of PWID respondents never use lubricant during vaginal or anal sex. Alcohol consumption was among 62.9% of PWID respondents with 32.2% injecting drugs at least once in a month or less. About 27.6% inject drug 2-4 times a month, 28.3% inject drugs 2-3 times a week and 11.9% inject drug 4 or more times a week. Sharing of needles or syringes is a risk factors for infection. The results indicate that sharing of needle or syringes is low (5.2%) among PWID respondent of the 2021 Sierra Leone IBBSS and 4.5% of PWID respondent reported ever been raped.

**Table 47: Sexual and behavioural risk factors and violence of PWID**

| **Sexual and behavioural risk factors and violence** | **Frequency** | **Percent** |
| --- | --- | --- |
| **In the last 6 months when you had sexual intercourse, did you or your partner ever put the condom on after you already started having sex (N=1072)** |  |  |
| Yes | 94 | 8.77 |
| No | 978 | 91.23 |
| **In the last 6 months when you had sexual intercourse, did the condom ever break/leak during sex or while pulling out? (N=1065)** |  |  |
| Yes | 49 | 4.60 |
| No | 1016 | 95.40 |
| **In the last 6 months when you had sexual intercourse, did you or your partner ever take the condom off before you were finished having sex? (N=1078)** |  |  |
| Yes | 184 | 17.07 |
| No | 894 | 82.93 |
| **In the last 6 months when you had sexual intercourse, did the condom ever slip off during sex or while pulling out? (N=1063)** |  |  |
| Yes | 40 | 3.76 |
| No | 1023 | 96.24 |
| **How often do you use lubricant during vaginal or anal sex? (N=1070)** |  |  |
| Always/Usually | 113 | 10.56 |
| Sometimes/Rarely | 274 | 25.61 |
| Never | 683 | 63.83 |
| **Alcohol use (N=1149)** |  |  |
| Never drank alcohol | 426 | 37.08 |
| Use alcohol | 723 | 62.92 |
| **How often do you Inject drugs (N=1132)** |  |  |
| Monthly or less | 365 | 32.24 |
| 2-4 times a month | 312 | 27.56 |
| 2-3 times a week | 320 | 28.27 |
| 4 or more time a week | 135 | 11.93 |
| **Ever shared needles or syringes (N=971)** |  |  |
| Yes | 50 | 5.15 |
| No | 921 | 94.85 |
| **Rape (N=1148)** |  |  |
| Yes | 52 | 4.53 |
| No | 1096 | 95.47 |

**HIV-related knowledge among PWID**

Table 48 present the HIV-related knowledge among PWID. Knowledge drive behavioural change and HIV-related knowledge among PWID respondents was assessed using series of questions. These questions are to establish the level of knowledge of participants about HIV on the myth and misconception that individuals and community hold about HIV and AIDS. The results show that more than half (52.5%, n=605) of PWID interviewed had moderate knowledge about HIV. Regarding knowledge of HIV prevention methods, 79.8% (n=921) of PWID know that limiting sexual intercourse to one uninfected partner can reduce the chances of getting HIV and 78.6%(n=907) of PWID know that it is possible for a healthy-looking person to have HIV. Approximately 64% of the PWID indicated correctly that people cannot get HIV by sharing food with someone who is infected and 88.1% answered correctly if people can get HIV because of supernatural means (juju, witchcraft, black magic etc). Almost all the PWID respondents (94.5%) responded that people can get HIV by getting injection with a needle that was already used by someone else. Prevention of mother-to-child transmission (PMTCT) of HIV is critical to break the chain of new infection. All respondents were asked if an infected mother can transmit the virus to her unborn child during pregnancy, 55.5% answered correctly. Respectively, 54.7% and 62.0% of PWID respondents in indicated that a woman with HIV transmit the virus to her new-born child during pregnancy and through breastfeeding. A little over half of the PWID respondents reported correctly that there are special drugs that a doctor or nurse can give to a woman infected with HIV to reduce the risk of transmission to the baby. Almost six out of ten (59.6%) of PWID respondents answered correctly that hey have heard about a special antiretroviral drug that people infected with HIV can get from a doctor or nurse to help live longer. Overall knowledge about HIV-related risk among PWID respondents to the 2021 Sierra Leone IBBSS moderate to high. A total of 16.6% of PWID respondents have overall low knowledge, 52.5% have moderate knowledge and 30.9% have high knowledge.

**Table 48: HIV-related knowledge among PWID**

| **HIV-related knowledge among PWID** | **Frequency** | **Percent** |
| --- | --- | --- |
| **Can people reduce their chance of getting HIV by having just one uninfected sex partner who has no other sex partners? (N=1154)** |  |  |
| Wrong | 233 | 20.19 |
| Correct | 921 | 79.81 |
| **Can people get HIV from mosquito bites? (N=1152)** |  |  |
| Wrong | 439 | 38.11 |
| Correct | 713 | 61.89 |
| **Can people reduce their chance of getting HIV by using a condom every time they have sex? (N=1154)** |  |  |
| Wrong | 204 | 17.68 |
| Correct | 950 | 82.32 |
| **Can a person get HIV by sharing food with someone who is infected? (N=1154)** |  |  |
| Wrong | 416 | 36.05 |
| Correct | 738 | 63.95 |
| **Can people get HIV because of supernatural means (juju, witchcraft, black magic etc)? (N=1154)** |  |  |
| Wrong | 137 | 11.87 |
| Correct | 1017 | 88.13 |
| **Is it possible for a healthy-looking person to have HIV? (N=1154)** |  |  |
| Wrong | 247 | 21.40 |
| Correct | 907 | 78.60 |
| **Can a person get HIV by getting injections with a needle that was already used by someone else? (N=1154)** |  |  |
| Wrong | 63 | 5.46 |
| Correct | 1091 | 94.54 |
| **Can an infected mother transmit HIV to her unborn child during pregnancy? (N=1154)** |  |  |
| Wrong | 514 | 44.54 |
| Correct | 640 | 55.46 |
| **Can a woman with HIV transmit the virus to her newborn child during delivery? (N=1154)** |  |  |
| Wrong | 523 | 45.32 |
| Correct | 631 | 54.68 |
| **Can a woman with HIV transmit the virus to her newborn child through breastfeeding? (N=1154)** |  |  |
| Wrong | 439 | 38.04 |
| Correct | 715 | 61.96 |
| **Are there any special drugs that a doctor or a nurse can give to a woman infected with HIV to reduce the risk of transmission to the baby? (N=1154)** |  |  |
| Wrong | 520 | 45.06 |
| Correct | 634 | 54.94 |
| **Have you heard about special antiretroviral drugs (e.g. ARV, nevirapine, zidovudine, lamivudine) that people infected with HIV can get from a doctor or a nurse to help them live longer? (N=1154)** |  |  |
| Wrong | 466 | 40.38 |
| Correct | 688 | 59.62 |
| **Overall knowledge scores (N=1152)** |  |  |
| >=6 Low knowledge | 191 | 16.58 |
| 7-9 Moderate | 605 | 52.52 |
| 10-12 High knowledge | 356 | 30.90 |

**PWID exposure to interventions**

Table 49 provides information on the PWIDs exposure to HIV interventions. A little over half of the PWIDs (51.3%: n=585) indicated that they are aware of civil society organization(s) that deliver non-medical assistance or advice to PWID. Nearly a third of the PWID (31.7%: n=143) reported been in contact with a health peer navigator in the community in the last six months preceding the study. Minority of the PWIDs (31.88%: n=143) reported of attending meetings to discuss HIV and/or AIDS in the last six months preceding the survey.

**Table 49: PWID exposure to interventions**

| **Exposure to HIV interventions** | **Frequency** | **Percent** |
| --- | --- | --- |
| **Are you aware of any civil society or any organization(s) that deliver non-medical assistance or advice to PWID? (N=1141)** |  |  |
| Yes | 585 | 51.27 |
| No | 556 | 48.73 |
| **In the last 6 months, have you been in contact with a health peer navigator in the community? (N=451)** |  |  |
| Yes | 143 | 31.71 |
| No | 308 | 68.29 |
| **During the last 6 months, have you attended any meetings to discuss HIV and/or AIDS? (N=1148)** |  |  |
| Yes | 366 | 31.88 |
| No | 782 | 68.12 |

**HIV services for HIV positive patients**

Knowledge and adherence to care and support is important for HIV positive patients to improve their health conditions and to prevent infecting other people with HIV by enhancing prevention and management of HIV-related infections. From Table 50, 25.0% (n=3) HIV positive PWID patient are on treatment with a patient (33.3%, n=1) who sometimes forget to take his/her medication (ART). Only 25% (n=3) of the HIV positive patient are aware of where to get HIV advice. All the 3 (100%) HIV positive PWID patient on treatment have never stopped taking ART or reduced the dose without informing their prescriber because of feeling worse when they took the medication.

**Table 50: HIV services for HIV positive patients**

| **HIV services for HIV positive patients** | **Frequency** | **Percent** |
| --- | --- | --- |
| **Are you currently on treatment (N=12)** |  |  |
| **Yes** | 3 | 25.00 |
| **No** | 9 | 75.00 |
| **Do you sometimes forget to take your ART (N=3)** |  |  |
| Yes | 1 | 33.33 |
| No | 2 | 66.67 |
| **DO you know where people can get advice about HIV (N=12)** |  |  |
| Yes | 3 | 25.00 |
| No | 9 | 75.00 |
| **Have you ever stopped taking your ARTS or decreased the dose without**  **informing your prescriber because you felt worse when you took them (N=3)** |  |  |
| Yes | **0** | **0.00** |
| No | 3 | 100.00 |

**HIV prevalence among PWID by sociodemographic/economic factors in the six regional headquarter towns in Sierra Leone**

This section presents both the crude and the adjusted HIV prevalence estimate and the corresponding 95% confidence interval among PWIDs in the six regional headquarter towns in Sierra Leone. The crude estimate from the logit-transformed confidence intervals showed that the overall prevalence of HIV among PWIDs in the six regional headquarter towns was 3.2% (95% CI: 2.3-4.4). The RDS weighted design-based adjusted HIV prevalence among the TGs was 4.2% (95% CI: 2.7-6.4). Table 51 shows both the adjusted and the unadjusted HIV prevalence estimates and sociodemographic determinants of HIV among PWIDs in Sierra Leone. The HIV prevalence estimates differed by sex at birth, province and educational level of the PWIDs. The estimates of HIV prevalence were high among females who inject drugs (16.9%; 95% CI: 8.1-32.0%) compared to males (2.9%; 95% CI: 1.7-4.9). The prevalence of HIV infection was approximately 8 times higher among female injecting drug users compared to males (uPR=7.4; 95% CI:3.9- 14.1; p<0.001). PWIDs who had a senior high school (SHS) certificate had a lower prevalence of HIV (1.6%; 95% CI:0.5-4.5) compared to those with no or primary level education (7.1%, 95% CI: 3.3-14.5). The prevalence of HIV reduced by 74% (uPR=0.26; 95% CI: 0.11-0.62; p<0.05) among PWIDs with SHS certificate compared to those with no formal or those with only primary level education.

**Table 51: HIV prevalence among PWID by sociodemographic characteristics**

|  | Crude/unadjusted estimate of HIV prevalence | RDS design-based weighted estimated | Unadjusted prevalence ratio from double selection Lasso Poisson Regression Model adjusting for sampling weight from RDS | Unadjusted Odds ratio from Firth Penalized maximum likelihood logistic regression model adjusting for sampling weight from RDS |
| --- | --- | --- | --- | --- |
| **Sociodemographic characteristics** | HIV+ (%) [95% CI] | HIV+ (%) [95% CI] | uPR [95% CI] | uOR [95% CI] |
| **Age in years** |  |  |  |  |
| 18-24 years | 3.39[2.17- 5.26] | 4.72[2.51- 8.70] | 1.00 | 1.00 |
| 25-29 years | 1.19[0.45-3.12] | 1.76[0.56 -5.41] | 0.35[0.12-1.02] | 0.40[0.14- 1.12] |
| 30+ | 5.45[3.25- 8.99] | 5.76[3.06- 10.59] | 1.61 [0.82- 3.15] | 1.74[ 0.86-3.51] |
| **Sex at birth** |  |  |  |  |
| Male | 2.15[1.43 -3.22] | 2.89[1.68- 4.90] | 1.00 | 1.00 |
| Female | 16.67[10.12- 26.21] | 16.91[8.10-31.99] | 7.40[3.89 - 14.08]*** | 8.71[4.32- 17.56]*** |
| **Education level** |  |  |  |  |
| None/primary | 5.70[3.18 - 10.00] | 7.06[3.28-14.53] | 1.00 | 1.00 |
| JHS | 5.12[2.99-8.62] | 6.04[2.86- 12.30] | 0.88[0.40- 1.93] | 0.87[0.39-1.97] |
| SHS/Tech | 1.44 [0.75- 2.74] | 1.56[0.53- 4.49] | 0.26[0.11- 0.62]* | 0.25[0.11-0.61]* |
| Higher | 5.00[1.89 -12.59] | 9.55[3.25- 24.93] | 0.93[0.30 - 2.87] | 0.10[0.32- 3.08] |
| **Income** |  |  |  |  |
| <500000 | 3.64[2.16 - 6.05] | 5.98[3.07- 11.34] | 1.00 | 1.00 |
| 500000+ | 3.47[1.45 - 8.09] | 3.31[1.04 -10.02] | 0.95[0.35-2.61] | 1.12[0.41-3.07] |
| **Employment status** |  |  |  |  |
| Employed | 3.50[2.24- 5.42] | 4.92[2.77- 8.57] | 1.00 |  |
| Unemployed | 2.96[1.87 -4.64] | 3.47[1.74- 6.78] | 0.84[0.45 -1.59] | 0.85[0.44- 1.62] |
| **Marital status** |  |  |  |  |
| Married/divorced etc | 4.40[2.82 -6.79] | 4.85[2.58- 8.93] | 1.00 | 1.00 |
| Single | 2.49 [ 1.58- 3.92] | 3.79[2.07- 6.83] | 0.57[0.30-1.07] | 0.54[0.28- 1.03] |
| **Religion** |  |  |  |  |
| Christian | 3.83[2.28-6.36] | 4.28[2.07- 8.65] | 1.00 | 1.00 |
| Moslem | 2.92[1.95-4.36] | 4.09[2.36-7.01] | 0.76[0.40- 1.47] | 0.74[0.38- 1.44] |
| **Ethnicity** |  |  |  |  |
| Mende | 2.93 [1.53- 5.54] | 2.36[1.03- 5.33] | 1.00 | 1.00 |
| Temne | 2.26[1.18-4.29] | 3.54[1.57- 7.80] | 0.77[0.31- 1.92] | 0 .76[0.30-1.89] |
| Others | 4.24[2.72- 6.56] | 5.90[3.16- 10.74 | 1.45[0.66- 3.16] | 1.42[0.65- 3.13] |
| **District** |  |  |  |  |
| Bombali District | 7.55[4.33-12.83] | 9.61[4.74- 18.51] | 1.00 | 1.00 |
| Port Loko | 1.74[0.56 - 5.27] | 1.49 [0.31-6.89] | 0.30[0.08- 1.09] | 0 .33[0.09-1.14] |
| Kenema | 4.67[2.24 - 9.47] | 5.03[2.00 -12.09] | 0.72[0.29-1.81] | 0.73[0.28-1.90] |
| Bo | 0.63 [0.09- 4.36] | 1.15[0.16-7.73] | 0.09[0.01- 0.68]* | 0.12[0.02-0.66]* |
| Western Area Urban | 4.76[2.78 -8.03] | 5.50 [2.53-11.56] | 0.72[0.33-1.57] | 0.71[0.31- 1.61] |
| Wester Area Rural | 0.91[0.13- 6.18] | 2.81[0.40- 17.33] | 0.15[0.02- 1.18] | 0.20[0.04-1.13] |
| **Province** |  |  |  |  |
| Northern | 4.53[2.75 -7.38] | 7.66[3.88- 14.57] | 1.00 |  |
| Eastern | 2.47[1.18 - 5.10] | 1.91 [0.76- 4.74] | 0.50[0.20- 1.25] | 0 .51[0.21-1.23] |
| Southern | 0.63[0.09-4.36] | 1.15[0.16-7.73] | 0.13[0.02-0.96]* | 0.178[0.03- 0.96]* |
| Western | 3.66[2.18 -6.08] | 4.88[2.37-9.77] | 0.83[0.41- 1.69] | 0.83[0.40-1.72] |

Abbreviation: PWID: People who inject drugs, uPR: Unadjusted Prevalence Ratio, our: Unadjusted odds ratio; CI: Confidence interval. P-value notation: ***p<0.001, **p<0.01, *p<0.05.

**HIV prevalence among PWID by sexual history**

Table 52 shows the prevalence of HIV among PWIDs by sexual history. Among those PWIDs who have ever anal sex, the HIV prevalence was higher among those aged less than 18 years (22.6%; 95% CI:10.7-41.6) compared to those 18 years and above (2.5%; 95% CI:0.3-17.9). The prevalence of HIV was also higher among PWIDs who had ever had anal sex with a woman (4.7%; 95% CI: 2.2-9.6) compared to those who with no such experience (2.0%; 95% CI: 0.9-4.3). The detailed distribution of HIV prevalence by sexual history can be found in Table 8.

**Table 52: HIV prevalence among PWID by sexual history**

|  | Crude/unadjusted estimate of HIV prevalence | **RDS design-based weighted estimated** | **Unadjusted prevalence ratio from double selection Lasso Poisson Regression Model adjusting for sampling weight from RDS** | **Unadjusted Odds ratio from Firth Penalized maximum likelihood logistic regression model adjusting for sampling weight from RDS** |
| --- | --- | --- | --- | --- |
| **Sexual history** | **HIV+ (%) [95% CI]** | **HIV+ (%) [95% CI]** | **uPR [95% CI]** | **uOR [95% CI]** |
| **Ever had a vaginal sex** |  |  |  |  |
| Yes | 2.18 [1.45-3.25] | 2.94 [1.72-5.00] | NA | NA |
| **Ever had anal sex with a woman** |  |  |  |  |
| Yes | 2.80 [1.46-5.30] | 4.67 [2.20-9.62] | 1 | 1 |
| No | 1.87 [1.11-3.13] | 2.02 [0.93-4.33] | 0.67 [0.29-1.53] | 0.67 [0.29-1.55] |
| **Number of women you had vaginal sex with in the last three month** |  |  |  |  |
| None | 2.08 [1.12-3.83] | 1.16 [0.51-2.61] | 1 | 1 |
| Only 1 | 2.75 [1.38-5.40] | 5.56 [2.54-11.74] | 1.32 [0.53-3.3] | 1.39 [0.55-3.48] |
| 2 or more | 1.67 [0.70-3.96] | 3.33 [1.13-9.42] | 0.80 [0.28-2.33] | 0.88 [0.31-2.50] |
| **Of these, how many did you not use condom** |  |  |  |  |
| None | 2.13 [1.11-4.04] | 2.24 [0.98-5.04] | 1 | 1 |
| Only 1 | 2.26 [1.13-4.46] | 2.76 [1.04-7.13] | 1.03 [0.40-2.64] | 1.04 [0.41-2.66] |
| 2 or more | 2.02 [0.84-4.76] | 4.49 [1.53-12.49] | 1.00 [033-3.03] | 1.05 [0.35-3.03] |
| **Age at first anal sex with a man** |  |  |  |  |
| Less than 18 years | 20.63 [12.28-32.57] | 22.63 [10.73-41.57] | 1 | 1 |
| 18+ | 5.56 [0.75-31.31] | 2.53 [0.31-17.87] | 0.27 [0.04-1.95] | 0.33 [0.57-1.92] |
| **Ever been tested for HIV** |  |  |  |  |
| No | 3.38 [2.08-5.45] | 4.71 [2.48-8.76] | 1 | 1 |
| Yes | 3.10 [2.03-4.71] | 3.66 [2.02-6.54] | 0.99 [0.52-1.87] | 0.98 [0.51-1.90] |

Abbreviation: PWID: People who inject drugs, uPR: Unadjusted Prevalence Ratio, our: Unadjusted odds ratio; CI: Confidence interval. P-value notation: ***p<0.001, **p<0.01, *p<0.05. NA: No HIV recorded in other category of the indicator variable

**HIV prevalence among PWID by sexual and behavioural risk factors**

Table 53 presents the HIV prevalence among PWIDs by sexual and behavioural risk factors. The prevalence of HIV was higher among PWIDs who have ever shared needles or syringes (6.4%; 95% CI: 1.6-22.1) compared to those PWIDs who do not share needles or syringes (4.2%; 95% CI: 2.6-6.8). The prevalence of HIV reduced by approximately 61% among the non-needle or syringe sharing PWIDs compared to those that shared these objects (uPR=0.39; 95% CI: 0.14-0.91, p<0.05). The prevalence was also higher among PWIDs that use alcohol (4.8%, 95% CI: 2.8-8.0) compare those that do not drink alcohol (3.1%; 95% CI: 1.4-6.9). The prevalence of HIV was also higher among PWIDs who have ever experience sexual violence including rape (8.9%; 95% CI: 2.2-29.3) compared to those PWIDs who had not experience sexual violence (4.0%; 95% CI: 2.5-6.2).

**Table 53: HIV prevalence among PWID by sexual and behavioural risk factors**

|  | Crude/unadjusted estimate of HIV prevalence | RDS design-based weighted estimated | Unadjusted prevalence ratio from double selection Lasso Poisson Regression Model adjusting for sampling weight from RDS | Unadjusted Odds ratio from Firth Penalized maximum likelihood logistic regression model adjusting for sampling weight from RDS |
| --- | --- | --- | --- | --- |
| **Behavioural risk factors** | **HIV+ (%) [95% CI]** | **HIV+ (%) [95% CI]** | **uPR [95% CI]** | **uOR [95% CI]** |
| **In the last 6 months when you had sexual intercourse, did you or your partner ever put the condom on after you already started having sex** |  |  |  |  |
| Yes | 1.06 [0.15-7.18] | 1.40 [0.20-9.36] | 1 | 1 |
| No | 3.48 [2.49-4.83] | 4.26 [2.7-6.66] | 3.27 [0.45-23.63] | 2.26 [0.43-11.75] |
| **In the last 6 months when you had sexual intercourse, did the condom ever break/leak during sex or while pulling out?** |  |  |  |  |
| Yes | 6.12 [1.99-17.35] | 13.36 [3.32-40.89] | 1 | 1 |
| No | 3.15 [2.23-4.42] | 3.58 [2.25-5.64] | 0.51 [0.16-1.62] | 0.44 [0.14-1.37] |
| **In the last 6 months when you had sexual intercourse, did you or your partner ever take the condom off before you were finished having sex?** |  |  |  |  |
| Yes | 3.26 [1.47-7.07] | 2.93 [1.02-8.14] | 1 | 1 |
| No | 3.13 02.17-4.50] | 4.14 [2.52-6.73] | 0.96 [0.40-2.29] | 0.89 [0.38-2.13] |
| **In the last 6 months when you had sexual intercourse, did the condom ever slip off during sex or while pulling out?** |  |  |  |  |
| Yes | 5.0 [1.25-17.93] | 1.84 [0.31-10.21] | **1** | **1** |
| No | 3.23 [2.30-4.50] | 4.10 [2.61-6.39] | 0.65 [0.16-2.60] | 0.51 [0.14-1.93] |
| **How often do you use lubricant during vaginal or anal sex?** |  |  |  |  |
| Always/Usually | 0.88 [0.12-6.02] | 3.20 [0.45 -19.34] | 1 | 1 |
| Sometimes/Rarely | 5.47 [3.33-8.89] | 6.92 [3.31-13.88] | 6.19 [0.83-46.32] | 4.95 [0.91-27.05] |
| Never | 2.78 [1.78-4.32] | 3.66 [2.05- 6.47] | 3.14 [0.42-23.27] | 2.44 [0.45-13.07] |
| **Alcohol use** |  |  |  |  |
| Never drank alcohol | 2.82 [1.61-4.90] | 3.14 [1.39-6.93] | 1 | 1 |
| Use alcohol | 3.46 [2.35-5.07] | 4.79 [2.83-7.97] | 1.23 [0.62-2.42] | 1.22 [0.61-2.42] |
| **How often do you Inject drugs** |  |  |  |  |
| Monthly or less | 1.64 [0.74-3.61] | 2.78 [1.01-7.41] |  |  |
| 2-4 times a month | 5.45 [3.41-8.59] | 6.84 [3.67-12.40] | 3.31 [1.32-8.31] | 3.33 [1.34-8.30] |
| 2-3 times a week | 3.13 [1.69-5.71] | 4.30 [1.76-10.16] | 1.90 [0.70-5.17] | 1.82 [0.67-4.90] |
| 4 or more time a week | 2.22 0.72-6.67] | 1.24 [0.33-4.57] | 1.35 [0.34-5.33] | 1.45 [0.39-5.41] |
| **Ever shared needles or syringes** |  |  |  |  |
| Yes | 8.00 [3.03-19.48] | 6.37 [1.61 -22.09] | 1 | 1 |
| No | 3.15 [2.20-4.50] | 4.23 [2.60-6.81] | 0.39 [0.14-0.91]* | 0.34[0.12-0.97]* |
| **Rape** |  |  |  |  |
| Yes | 3.85 [0.96-14.14] | 8.86 [2.23-29.28] | 1 | 1 |
| No | 3.19 [2.30-4.42] | 3.96 [2.49-6.24] | 0.83 [0.21-3.36] | 0.66 [0.18-2.46] |

Abbreviation: PWID: People who inject drugs, uPR: Unadjusted Prevalence Ratio, our: Unadjusted odds ratio; CI: Confidence interval. P-value notation: ***p<0.001, **p<0.01, *p<0.05.

**HIV-related knowledge among PWID**

Table 54 shows the HIV prevalence and HIV-related knowledge among PWIDs in the six regional headquarter towns. PWIDs who provided correct responses to the following questions had a lower HIV prevalence compared to those who provided wrong responses: “Can people reduce their chance of getting HIV by having just one uninfected sex partner who has no other sex partners?”, “can people get HIV from mosquito bites?”, “can people reduce their chance of getting HIV by using a condom every time they have sex?”, “can a person get HIV by getting injections with a needle that was already used by someone else?”,etc. In conclusion, increasing level of HIV-related knowledge correlates with a lower HIV-related prevalence.

**Table 54: HIV-related knowledge among PWID**

|  | Crude/unadjusted estimate of HIV prevalence | **RDS design-based weighted estimated** | **Unadjusted prevalence ratio from double selection Lasso Poisson Regression Model adjusting for sampling weight from RDS** | **Unadjusted Odds ratio from Firth Penalized maximum likelihood logistic regression model adjusting for sampling weight from RDS** |
| --- | --- | --- | --- | --- |
| **HIV-related knowledge among PWID** | **HIV+ (%) [95% CI]** | **HIV+ (%) [95% CI]** | **uPR [95% CI]** | **uOR [95% CI]** |
| **Can people reduce their chance of getting HIV by having just one uninfected sex partner who has no other sex partners?** |  |  |  |  |
| Wrong | 4.72 [2.63-8.33] | 6.25 [2.96-12.70] | 1 | 1 |
| Correct | 2.82 [1.93-4.12] | 3.56 [2.06-6.08] | 0.60 [0.30-1.19] | 0.59 [0.29-1.19] |
| **Can people get HIV from mosquito bites?** |  |  |  |  |
| Wrong | 4.56 [2.96-6.96] | 6.15 [3.43-10.79] | 1 | 1 |
| Correct | 2.38 [1.49-3.80] | 2.78 [1.44-5.33] | 0.52 [0.28-0.99]* | 0.53 [0.28-1.12] |
| **Can people reduce their chance of getting HIV by using a condom every time they have sex?** |  |  |  |  |
| Wrong | 3.43 [1.64-7.03] | 6.89 [2.92-15.43] | 1 | 1 |
| Correct | 3.16 [2.22-4.48] | 3.49 [2.11-5.72] | 0.92 [0.41-2.07] | 0.90 [0.40-2.14] |
| **Can a person get HIV by sharing food with someone who is infected?** |  |  |  |  |
| Wrong | 3.61 [2.18-5.90] | 4.04 [2.01-7.92] | 1 | 1 |
| Correct | 2.98 [1.97-4.49] | 4.21 [2.40-7.31] | 0.83 [0.43-1.58] | 0.81 [0.42-1.56] |
| **Can people get HIV because of supernatural means (juju, witchcraft, black magic etc)?** |  |  |  |  |
| Wrong | 5.84 [2.94-11.25] | 8.12 [3.01-20.12] | 1 | 1 |
| Correct | 2.85 [1.99-4.07] | 3.54 [2.19-5.67] | 0.49 [0.23-1.05] | 0.47 [0.22- 1.04] |
| **Is it possible for a healthy-looking person to have HIV?** |  |  |  |  |
| Wrong | 3.64 [1.91-6.86] | 7.20 [3.38-14.68] | 1 | 1 |
| Correct | 3.09 [2.14-4.44] | 3.13 [1.87-5.20] | 0.90 [0.44-1.83] | 0.86 [0.41-1.84] |
| **Can a person get HIV by getting injections with a needle that was already used by someone else?** |  |  |  |  |
| Wrong | 1.59 [0.22-10.44] | 6.26 [0.90-33.03] | 1 | 1 |
| Correct | 3.30 [2.39-4.54] | 4.02 [2.57-6.22] | 2.08 [0.29-14.93] | 1.50 [0.29-7.83] |
| **Can an infected mother transmit HIV to her unborn child during pregnancy?** |  |  |  |  |
| Wrong | 2.53 [1.47-4.31] | 3.56 [1.83-6.83] | 1 | 1 |
| Correct | 3.75 [2.52-5.54] | 4.67 [2.60-8.23] | 1.48 [0.76-2.88] | 1.52 [0.77-2.98] |
| **Can a woman with HIV transmit the virus to her newborn child during delivery?** |  |  |  |  |
| Wrong | 3.06 [1.88-4.94] | 4.79 [2.67-8.44 | 1 | 1 |
| Correct | 3.33 [2.18-5.05] | 3.53 [1.80-6.81] | 1.09 [0.57-2.06] | 1.14 [0.59-2.19] |
| **Can a woman with HIV transmit the virus to her newborn child through breastfeeding?** |  |  |  |  |
| Wrong | 2.73 [1.56-4.75] | 4.46 [2.24-8.69] | 1 | 1 |
| Correct | 3.50 [2.37-5.13] | 3.93 [2.22-6.86] | 1.28 [0.65-2.52] | 1.33 [0.66-2.66] |
| **Are there any special drugs that a doctor or a nurse can give to a woman infected with HIV to reduce the risk of transmission to the baby?** |  |  |  |  |
| Wrong | 2.69 [1.60-4.50] | 3.67 [1.77-7.45] | 1 | 1 |
| Correct | 3.63 [2.42- 5.40] | 4.64 [2.71-7.84] | 1.44 [0.75-2.78] | 1.44 [0.74-2.92] |
| **Have you heard about special antiretroviral drugs (e.g. ARV, nevirapine, zidovudine, lamivudine) that people infected with HIV can get from a doctor or a nurse to help them live longer?** |  |  |  |  |
| Wrong | 2.36 [1.31-4.21] | 3.34 [1.62-6.77] | 1 | 1 |
| Correct | 3.78 [2.58-5.49] | 4.80 [2.76-8.24] | 1.60 [0.80-3.21] | 1.68 [0.83-3.39] |
| **Overall knowledge scores** |  |  |  |  |
| Scores less than 6: Low knowledge | 4.19 [2.11-8.16] | 7.79 [3.55-16.22] | 1 | 1 |
| Scores between 7-9: Moderate | **3.14 [2.01-4.87]** | **4.05 [2.25-7.20]** | **0.78 [0.35-1.72]** | **0.74 [0.33-1.70]** |
| Scores between: 10-12 High knowledge | **2.81 [1.52-5.14]** | **1.40 [0.65-2.99]** | **0.75 [0.32-1.79]** | **0.73 [0.29-1.88]** |

Abbreviation: PWID: People who inject drugs, uPR: Unadjusted Prevalence Ratio, our: Unadjusted odds ratio; CI: Confidence interval. P-value notation: ***p<0.001, **p<0.01, *p<0.05.

**Multivariable analysis of factors associated with HIV among PWID**

The results from the multivariable Poisson double selection least absolute shrinkage and selection and the binary logistic firth penalized maximum likelihood regression showed that HIV prevalence was higher among females compared to male PWIDs. The prevalence of HIV among female PWIDs was approximately 5 times as high as the prevalence of HIV among males (aPR= 5.4; 95% CI:2.9-9.8). The detailed multivariable analysis of factors associated with HIV among PWIDs can be found in Table 55

**Table 55: Multivariable analysis of factors associated with HIV among PWID**

| **Sociodemographic characteristics** | **Adjusted prevalence ratio from double selection Lasso Poisson Regression Model adjusting for sampling weight from RDS**  **aPR [95% CI]** | **Adjusted Odds ratio from Firth Penalized maximum likelihood logistic regression model adjusting for sampling weight from RDS**  **aOR [95% CI]** |
| --- | --- | --- |
| **Sex at birth** |  |  |
| Male | 1 | 1 |
| Female | 5.35 [2.92-9.79]*** | 6.30 [3.04-13.03]*** |
| **Education level** |  |  |
| None/primary | 1 | 1 |
| JHS | 0.92 [0.45-1.88] | 0.91 [0.40-2.12] |
| SHS/Tech | 0.40 [0.18-0.89]* | 0.38 [0.15-0.97]* |
| Higher | 1.15 [0.38-3.53] | 1.22 [0.36-4.20] |
| **Province** |  |  |
| Northern | 1 | 1 |
| Eastern | 0.57 [0.23-1.38] | 0.55 [0.21-1.43] |
| Southern | 0.16 [0.02-1.30] | 0.22 [0.04-1.20] |
| Western | 0.67 [0.34-1.32] | 0.65 [0.30-1.44] |
| **Can people get HIV from mosquito bites?** |  |  |
| Wrong | 1 | 1 |
| Correct | 0.59 [0.33-1.05] | 0.56 [0.28-1.13] |

Abbreviation: PWID: People who inject drugs, aPR: Adjusted Prevalence Ratio, aOR: Adjusted odds ratio; CI: Confidence interval. P-value notation: ***p<0.001, **p<0.01, *p<0.05.
